# Sequential lipidomic, metabolomic, and proteomic analyses of serum, liver, and heart tissue specimens from peroxisomal biogenesis factor 11α knockout mice

**DOI:** 10.1007/s00216-021-03860-0

**Published:** 2022-01-27

**Authors:** Vannuruswamy Garikapati, Claudia Colasante, Eveline Baumgart-Vogt, Bernhard Spengler

**Affiliations:** 1 Institute of Inorganic and Analytical Chemistry, Justus Liebig University Giessen, 35392 Giessen, Germany; 2 Institute for Anatomy and Cell Biology II, Division of Medical Cell Biology, Justus Liebig University Giessen, 35392 Giessen, Germany

**Keywords:** Lipidomics, Metabolomics, Proteomics, Mass spectrometry, Peroxisomes, Peroxisomal biogenesis disorders

## Abstract

Peroxisomes are versatile single membrane-enclosed cytoplasmic organelles, involved in reactive oxygen species (ROS) and lipid metabolism and diverse other metabolic processes. Peroxisomal disorders result from mutations in *Pex* genes-encoded proteins named peroxins (PEX proteins) and single peroxisomal enzyme deficiencies. The PEX11 protein family (α, β, and γ isoforms) plays an important role in peroxisomal proliferation and fission. However, their specific functions and the metabolic impact caused by their deficiencies have not been precisely characterized. To understand the systemic molecular alterations caused by peroxisomal defects, here we utilized untreated peroxisomal biogenesis factor 11α knockout (*Pex11α* KO) mouse model and performed serial relative-quantitative lipidomic, metabolomic, and proteomic analyses of serum, liver, and heart tissue homogenates. We demonstrated significant specific changes in the abundances of multiple lipid species, polar metabolites, and proteins and dysregulated metabolic pathways in distinct biological specimens of the *Pex11α* KO adult mice in comparison to the wild type (WT) controls. Overall, the present study reports comprehensive semi-quantitative molecular omics information of the *Pex11α* KO mice, which might serve in the future as a reference for a better understanding of the roles of *Pex11α* and underlying pathophysiological mechanisms of peroxisomal biogenesis disorders.

**Graphical abstract:**

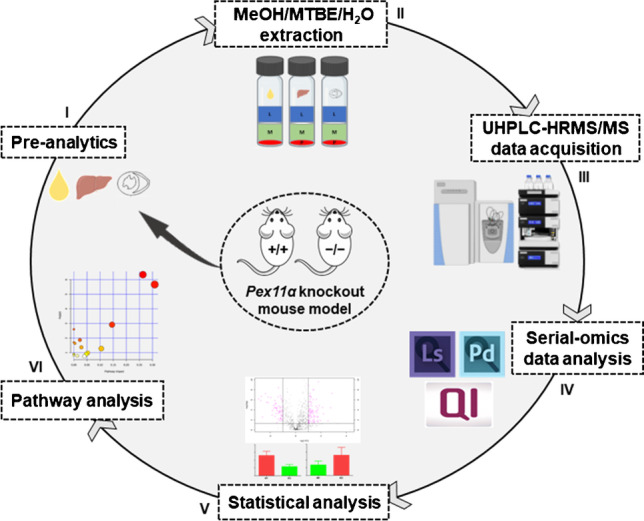

**Supplementary Information:**

The online version contains supplementary material available at 10.1007/s00216-021-03860-0.

## Introduction

Peroxisomes are small (0.1–0.5 µm in diameter), single membrane bound, subcellular organelles present in almost every eukaryotic cell. They play a central role in a wide variety of vital metabolic functions, namely (i) β-oxidation of very long-chain fatty acids (e.g., VLCFAs, ≥ C22) and of bioactive secondary lipid mediators (such as prostaglandins and leukotrienes), (ii) α- (e.g., phytanic acid) and β-oxidation (e.g., pristanic acid) of branched-chain fatty acids, (iii) biosynthesis of ether-phospholipids (e.g., plasmalogens), cholesterol, dolichol, and conjugated bile acids, (iv) glyoxylate detoxification, and (v) breakdown of polyamines and purines [1]. The granular matrix of peroxisomes contains a large variety of oxidases, producing not only hydrogen peroxide (H_2_O_2_) during the conversion of their substrates but also the antioxidative marker enzyme catalase that cleaves H_2_O_2_ into water and oxygen, and protects the cell from excessive ROS production [2, 3].

The abundance, size, shape, protein/enzyme composition, and functions of peroxisomes can differ based on the physiological condition and metabolic needs of the cell type, tissue, organ, and/or organism [3, 4]. Regulators of malleability and biogenesis of peroxisomes are a set of heterogeneous proteins referred to as peroxins (PEX proteins), located in the cytoplasm, the peroxisomal membrane as well as their matrix. They mediate all steps in peroxisomal biogenesis, such as (i) the formation of the peroxisomal membrane, (ii) the import of their membrane and matrix proteins, and (iii) the proliferation of the organelles. Hitherto, more than 32 distinct PEX proteins have been recognized (named with reference to their date of discovery), out of which at least 14 are conserved in mammalians [5, 6].

The functional importance of peroxisomal metabolism for health in humans is emphasized by the existence of numerous peroxisomal disorders. These devastating genetic human diseases are either resulting from mutations in *Pex* genes (peroxisomal biogenesis disorders, PBDs, or Zellweger spectrum disorders, ZSDs) or genes encoding single peroxisomal enzymes (peroxisomal enzyme deficiencies, PEDs) [1]. In both cases, the specific metabolic functions of multiple organs (such as brain, liver, kidney, adrenal gland, testis, bone, and many others) are severely disturbed, affecting the organism at a systemic level and often resulting in premature death. The processes that are usually affected are the fatty acid β- and α-oxidation, the plasmalogens biosynthesis, the glyoxylate metabolism, the bile acid synthesis, and the H_2_O_2_ metabolism [7, 8].

To study PBDs, Li and Baumgart et al. previously generated and bred *Pex11* (α and β) knockout mouse models and performed their basal characterization [9, 10]. Thereby, they also identified a third *Pex11* gene (*Pex11γ*). The three PEX11 protein isoforms (α, β, and γ) are responsible for peroxisome proliferation and fission by mediating elongation, tubulation, and constriction of pre-existing peroxisomes [11–13]. *Pex11β* is expressed constitutively throughout the tissues, whereas α and γ isoforms are tissue specific and pronounced considerably in the heart, liver, and kidney as well as testis [10, 11]. In contrast to the knockout of *Pex11β*, which induces a phenotype similar to Zellweger syndrome and neonatal lethality, mice with a *Pex11α* KO display a mild phenotype and are viable after birth [9, 10], enabling investigations of the impact of peroxisomal defects on the lipid, metabolite, and protein composition of various organs (e.g., liver, heart, kidney, lung, and brain) and biological fluids (e.g., serum) in adult mice. Indeed, adult *Pex11α* knockout mice when treated with a high-fat diet develop nonalcoholic fatty liver disease [14].

In systems biology, concurrent extraction and investigation of various biomolecules (including proteins, peptides, lipids, and polar metabolites) and integration of mass spectrometry-guided multi-level molecular omics data (such as lipidomics, metabolomics, peptidomics, and proteomics) of the same sample has become a promising strategy to improve the understanding of complex biological cascades [15]. A series of recent studies has demonstrated one-step extraction protocols for serial-omics analyses from a single piece of sample and has been applied in cancer [16], cardiovascular [17], pulmonary [18], plant [19], and toxicological research [20].

In order to characterize the metabolic alterations occurring due to *Pex11*alpha deficiency, we investigated changes in the lipidome, metabolome, and proteome of different biological specimens (serum, liver, and heart) from *Pex11α* KO adult mice using a liquid–liquid extraction method combined with untargeted sequential omics approaches by ultra-high-performance liquid chromatography equipped with high-resolution tandem mass spectrometry (UHPLC-HRMS/MS). Our study provides extensive semi-quantitative molecular information on the metabolic alterations in *Pex11α* KO mice, which will complement the understanding of the molecular functions of *Pex11α* and underlying pathophysiological mechanisms of peroxisomal biogenesis disorders.

## Materials and methods

### Materials

Ammonium bicarbonate, ammonium formate, bovine serum albumin, dithiothreitol (DTT), iodoacetamide (IAA), methyl tert-butyl ether (MTBE), thiourea, and urea were purchased from Sigma-Aldrich (Steinheim, Germany). LC–MS grade solvents acetonitrile (ACN), methanol (MeOH), water (H_2_O), 2-propanol/isopropyl alcohol (IPA), and formic acid were procured from Honeywell Riedel-de Haën (Seelze, Germany). The synthetic lipid internal standards were procured from Avanti Polar Lipids (Alabaster, AL, USA). Sequencing grade modified trypsin was obtained from Promega Corporation (Mannheim, Germany) and RapiGest SF surfactant from Waters Corporation (Milford, MA, USA). Bradford reagent was purchased from Bio-Rad Laboratories (CA, USA) and ZipTip_C18_ from Merck Millipore (MA, USA).

### Pre-analytics

Pathogen-free C57BL/6 J mice were obtained from the central animal facility of the Justus Liebig University Giessen, Germany. They were kept on a normal laboratory diet and water and maintained under standard environmental conditions. The generation, breeding, and basal characterization of *Pex11α* KO animals were described in details earlier [9]. Adult 3-month-old male mice (WT control and homozygous *Pex11α* KO) were narcotized using 3% isofluran and sacrificed by cervical dislocation. For the isolation of heart and liver, the animals were perfused with phosphate buffer saline through the left ventricle to remove the blood. Liver and heart tissues were dissected, snap-frozen immediately in liquid nitrogen, and stored at − 80 °C until further processing. Blood was extracted by cardiac puncture immediately following the cervical dislocation and left standing for 30 min at 37 °C to separate the serum from the red blood cells. Then, the samples were spun for 10 min at 1500 × *g* at 4 °C, and serum was collected and stored at − 80 °C until further use.

The animal experiments were performed in accordance with the German Animal Welfare Law and recommendations of institutional animal welfare officers. All experiments were approved by the German Government Commission of Animal Care (Justus Liebig University internal classification: JLU-Nr.: 616_M, Project ID: 1016 Peroxisomen).

### Liquid–liquid extraction

Ten milligrams of the snap-frozen liver and heart tissues from three biological replicates of WT control (*Pex11α*^+/+^) and homozygous *Pex11α* KO (*Pex11α*^−/−^) mice were homogenized in phosphate buffer saline solution (200 µl) using a tissue homogenizer (Retsch GmbH, Germany) with zirconia beads for 1 min at 20 Hz at 4 °C. 200 µl of liver or heart tissue homogenates and/or 80 µl of serum were transferred into respective glass vials and subjected to liquid–liquid extraction as reported earlier [16]. Briefly, 750 µl of ice-cold methanol (LC–MS grade) containing the following lipid standard mix: 56 pmol phosphatidylethanolamine (PE 17:0/14:1); 52.5 pmol phosphatidylglycerol (PG 17:0/14:1); 82.5 pmol phosphatidylinositol (PI 17:0/14:1); 90 pmol phosphatidylserine (PS 17:0/14:1); 56 pmol phosphatidylcholine (PC 17:0/14:1); 47.5 pmol lysophosphatidylcholine (LPC 17:1); 16.6 pmol lysophosphatidylinositol (LPI 17:1); 84.6 pmol lysophosphatidylserine (LPS 17:1); 50 pmol lysophosphatidylglycerol (LPG 17:1); 42.8 pmol lysophosphatidylethanolamine (LPE 17:1); 69.6 pmol sphingomyelin (SM d18:1/17:0); 50 pmol cholesteryl ester (ChE 19:0); 90.4 pmol ceramide (Cer d18:1/17:0); 77.6 pmol hexosylceramide (HexCer d18:1/12:0); 30 pmol triglyceride (TG 17:0/17:0/17:0) were added to the homogenate samples and vortexed vigorously for 1 min. After that, 2.5 ml of ice-cold MTBE (anhydrous, 99.8%) were added to each vial and vortexed for 1 h at room temperature. Later, 625 µl of ice-cold water (LC–MS grade) were added for phase separation, vortexed for 1 min, and centrifuged at 4,000 g for 15 min at 4 °C. After centrifugation, the upper non-polar (lipids) and lower polar (metabolites) phases were separately collected, transferred to fresh vials, dried out in vacufuge concentrator (ambient temperature), and stored at − 80 °C until further analysis. The lower sediment (protein) pellets were resuspended in 250 µl of lysis buffer containing 0.1% RapiGest, 1 M urea, 0.2 M thiourea, and 70 mM dithiothreitol in 50 mM of ammonium bicarbonate buffer solution. The resuspended solution was vortexed vigorously followed by centrifugation for 10 min at 14,000 g at 4 °C and the supernatants were collected and stored at − 80 °C until further processing.

### Lipidomics (upper organic phase)

Dried lipid extracts were resuspended in 100 µl of ACN:IPA:H_2_O buffer (65:30:5 v/v) and analyzed using a hybrid quadrupole orbital trapping mass spectrometer (Q Exactive; Thermo Fisher Scientific, Bremen, Germany). In brief, 10 µl of resuspended lipid extract were loaded on a reversed-phase ACQUITY UPLC HSS T3 (1.8 µm, 100 × 2.1 mm, Waters Corporation) column and separated using a Dionex UltiMate 3000 UHPLC system (Thermo Fisher Scientific), with a flow rate of 250 µl/min. The solvent system consisted of eluent A (H_2_O:ACN, 40:60 v/v) and eluent B (IPA:ACN, 90:10 v/v) both with 10 mM ammonium formate and 0.1% formic acid. Lipids were separated with a 28 min multi-step linear gradient of 30 to 100% eluent B (electronic supplementary material, ESM Table S1). The column temperature was set to 50 °C and autosampler to 15 °C. Lipidomic datasets were acquired separately in positive- and negative-ion mode in a data-dependent manner using the top-15 method (Full MS/ddMS^2^, Top15), modified from a previous study [21]. The optimized heated electrospray ionization (HESI-II) source and data-dependent acquisition (DDA) method parameters for lipidomics experiments in both ionization modes are provided in the ESM (Tables S2 and S3).

High-resolution and accurate mass lipidomic raw datasets were analyzed with LipidSearch (v4.2.23) software (Thermo Fisher Scientific) for identification and alignment [21, 22]. The precursor (MS) and product (MS/MS) mass tolerance was set to 5 ppm. The fragment match score (m-score) was set to 5 and the identification level (fragmentation grade) quality filters A, B, and/or C were considered. The optimized parameters used for lipid identification (independently in positive- and negative-ion mode) and alignment processes (to combine positive- and negative-ion mode search results) are indicated in the ESM (Table S4). Further, all lipid species identified/aligned using the LipidSearch software were filtered (filtering criteria described in the ESM Table S4 and Data file S1) to remove/minimize false positives, and inspected manually (accept, reject, or reassign) after computational analysis.

### Metabolomics (lower aqueous phase)

Dried polar metabolite extracts were resuspended in 100 µl of 15% aqueous methanol and a 10 µl injection volume was used. The separation was performed using a reversed-phase ACQUITY UPLC HSS T3 (1.8 µm, 100 × 2.1 mm, Waters Corporation) column at a flow rate of 300 µl/min of a solvent system consisted of eluent A (100% water) and eluent B (100% acetonitrile) both with 0.1% formic acid. Polar metabolites were separated with a 20 min multi-step linear gradient of 1 to 99% eluent B (ESM Table S5). In the case of serum metabolite analysis, eluent B was replaced by 100% methanol with 0.1% formic acid and a multi-step linear gradient of 1 to 95% in 14 min (ESM Table S6). The autosampler and column compartments were maintained at 4 °C and 40 °C, respectively. Metabolomic datasets were acquired using a hybrid quadrupole orbital trapping mass spectrometer (Q Exactive; Thermo Fisher Scientific, Bremen, Germany) individually in positive- and negative-ion mode in a data-dependent manner using the top-10 method (Full MS/ddMS^2^, Top10), adopted from a previous study [23]. The optimized HESI-II ion source and DDA method parameters for metabolomics experiments in both ionization modes are provided in the ESM (Tables S7 and S8).

Positive- and negative-ion mode polar metabolomic raw datasets were processed independently using the Progenesis QI (v2.4) software (Waters Corporation) with default parameters. The untargeted workflow used for data processing and analysis includes retention time alignment, peak picking, deconvolution, compound annotation, normalization, and relative-quantification [23]. The ion adducts of each feature were deconvoluted and annotated primarily using an in-house developed metabolite database with MetaScope search plug-in of Progenesis QI and further verified with MassBank of North America (MoNA), mzCloud, and EMBL-MCF mass spectral libraries using precursor ion accurate mass (MS) and fragmentation (MS/MS) patterns.

### Proteomics (lower solid phase)

Total protein concentrations in supernatants of resuspended pellets were measured with Bradford’s reagent and subjected to in-solution tryptic digestion followed by label-free quantitative bottom-up proteomics experiments as described elsewhere [24, 25]. Briefly, an equal amount of protein (50 μg) from WT control and *Pex11α* KO mouse liver and/or heart tissues were solubilized in 50 mM ammonium bicarbonate buffer (50 μl) containing 0.1% RapiGest and denatured at 80 °C for 15 min. Then, the proteins were reduced and alkylated with 100 mM DTT at 56 °C for 30 min, 200 mM IAA at ambient temperature in the dark for 30 min, respectively. After that, the proteins were digested with trypsin (1:20 protease-to-protein ratio) at 37 °C for overnight. The tryptic digestion reaction was stopped by incubating with concentrated formic acid (2 µl) for 10 min at 37 °C. The peptide digests were desalted by ZipTip_C18_, dried out using vacufuge concentrator, and reconstituted in 3% aqueous ACN with 0.1% formic acid for further MS analysis.

Peptide digests (2.5 μg) were loaded on a Kinetex C18 reversed-phase (2.6 µm, 100 × 2.1 mm, 100 A°, Phenomenex) column and separated using a Dionex UltiMate 3000 UHPLC system (Thermo Fisher Scientific), with a flow rate of 250 μl/min. The solvent system consisted of eluent A (100% water) and eluent B (100% acetonitrile) both with 0.1% formic acid. Peptides were separated with a 120 min multi-step linear gradient of 3 to 50% eluent B (ESM Table S9). The autosampler temperature was set to 4 °C and column at 40 °C. Proteomic datasets were acquired using a hybrid quadrupole orbital trapping mass spectrometer (Q Exactive; Thermo Fisher Scientific, Bremen, Germany) in positive-ion mode in a data-dependent manner using the top-10 method (Full MS/ddMS^2^, Top10), with slight modifications from our previous study [25]. The optimized parameters of HESI-II ion source and DDA method for proteomics experiments are provided in the ESM (Tables S10 and S11).

Proteomic raw datasets were processed using untargeted label-free processing and consensus quantitative workflow (ESM Fig. S1) of Proteome Discoverer (v2.2.0.388) software (Thermo Fisher Scientific). MS data files were searched against UniProt *Mus musculus* protein database (dated 15th September 2019, containing 17,422 target sequences and 9,908,993 residues) using Sequest HT search algorithm. The search was performed with peptide precursor (MS) and fragment (MS/MS) mass tolerance of 10 ppm, 0.02 Da respectively and trypsin as a protease with two missed cleavages and a strict target false discovery rate value of 0.01 (1% FDR). Search criteria also comprised carbamidomethylation (cysteine) as static modification, oxidation (methionine) and acetylation (N-terminal) as dynamic modifications. Unique and razor peptides were considered, precursor abundance values were normalized with total peptide amount, and replicate-based resampling imputed missing values. Abundance ratios were calculated using the pairwise ratio method and significant values were obtained from ANOVA hypothesis test (based on the background population of proteins or peptides). The common external protein contaminant list (MaxQuant database) was used to mark contaminants in the result file. As regulated, only proteins with (i) master group, (ii) high confidence, (iii) at least two peptides, (iv) no contaminants, (v) *p*-value of ≤ 0.05, and (vi) an abundance ratio of ≤ 0.5 and/or ≥ 2 (equivalent to twofold regulation) were considered.

### Bioinformatics analyses

#### Statistical analysis

Integrated chromatographic peak area values of the individual lipids and polar metabolites were exported as a matrix (samples in columns and features in rows) after manual curation, and statistical analyses were performed using freely accessible MetaboAnalyst (v4.0) [26]. Lipidomic datasets were normalized to the total lipid signal, as no true internal standards were added for all identified lipid species and/or lipid classes and some of the lipid classes were noted with very few lipid species identifications [27, 28]. Prior to the statistical and pathway analyses, both lipidomic and polar metabolomic datasets were checked for data integrity, outliers removed, normalized (total sum), transformed (generalized logarithmic transformation), scaled (Pareto and/or Range), and imputed (features with ˃ 50% missing values were removed, and remaining missing values were estimated with k-nearest neighbor algorithm). Univariate descriptive statistical methods including fold-change analysis (threshold less than or equal to 0.5 and/or greater than or equal to 2), *t*-test (FDR-adjusted *p*-value less than or equal to 0.05), and volcano plots (log2 fold changes versus -log10 FDR-adjusted *p*-values) were employed to select statistically significant lipids and/or polar metabolites. Lipid class levels (sum of the normalized relative abundance values of all measured lipid species in a particular lipid class) represented as mean ± standard deviation and two-tailed Student’s *t*-test (GraphPad Prism v6.01) were used for statistical comparison between the groups.

#### Pathway analysis

Differentially abundant lipids and polar metabolites (HMDB v4.0, Human Metabolome Database IDs as input) were combined and explored by metabolic pathway analysis for the identification of altered pathways. The analyses were performed using MetPA module of the MetaboAnalyst (v4.0) [29]. “*Mus musculus*” pathway (KEGG, Kyoto Encyclopedia of Genes and Genomes) library, “hypergeometric test” for “over-representation analysis,” and “relative-betweenness centrality” for “pathway topology analysis” were used to identify altered specific metabolic pathways.

#### PANTHER

Protein analysis through evolutionary relationships (PANTHER, v14.1) was used to sort proteins with differential abundance into diverse cellular components, molecular functions, biological processes, signalling pathways, and protein classes [30].

#### BiNGO

Gene ontology (GO) clustering analysis for the proteins displaying differential abundance was carried out using STRING (Search Tool for the Retrieval of Interacting Genes, v11.0) and later visualized in Cytoscape (v3.7.2) BiNGO (Biological Network Gene Ontology, v3.0.3) [31]. The significantly enriched functional categories (such as biological processes, molecular functions, and cellular components) from GO Slim_*Mus musculus* were uncovered by employing a hypergeometric test and multiple test correction to attain *p*-value ≤ 0.05 using the Benjamini–Hochberg FDR correction method inbuilt within the BiNGO plug-in [32].

## Results and discussion

### Overview of the study design

Peroxisomes are multifunctional dynamic organelles with heterogeneous protein/enzyme composition and abundance in distinct cell types and organs. PEX11 proteins (α, β, and γ isoforms) are involved in peroxisomal proliferation and division. However, their specific function in these processes and the underlying molecular mechanisms of pathological alterations due to their deficiency have not been completely clarified. To investigate the diverse molecular changes that occur due to *Pex11α* deficiency, we performed comprehensive semi-quantitative sequential omics analyses of serum, liver, and heart tissue specimens from WT control (*Pex11α*^+/+^) and untreated homozygous *Pex11α* KO (*Pex11α*^−/−^) adult mice (genotypes of animals were determined by polymerase chain reaction and results are shown in ESM Fig. S2).

**Fig. 1:**
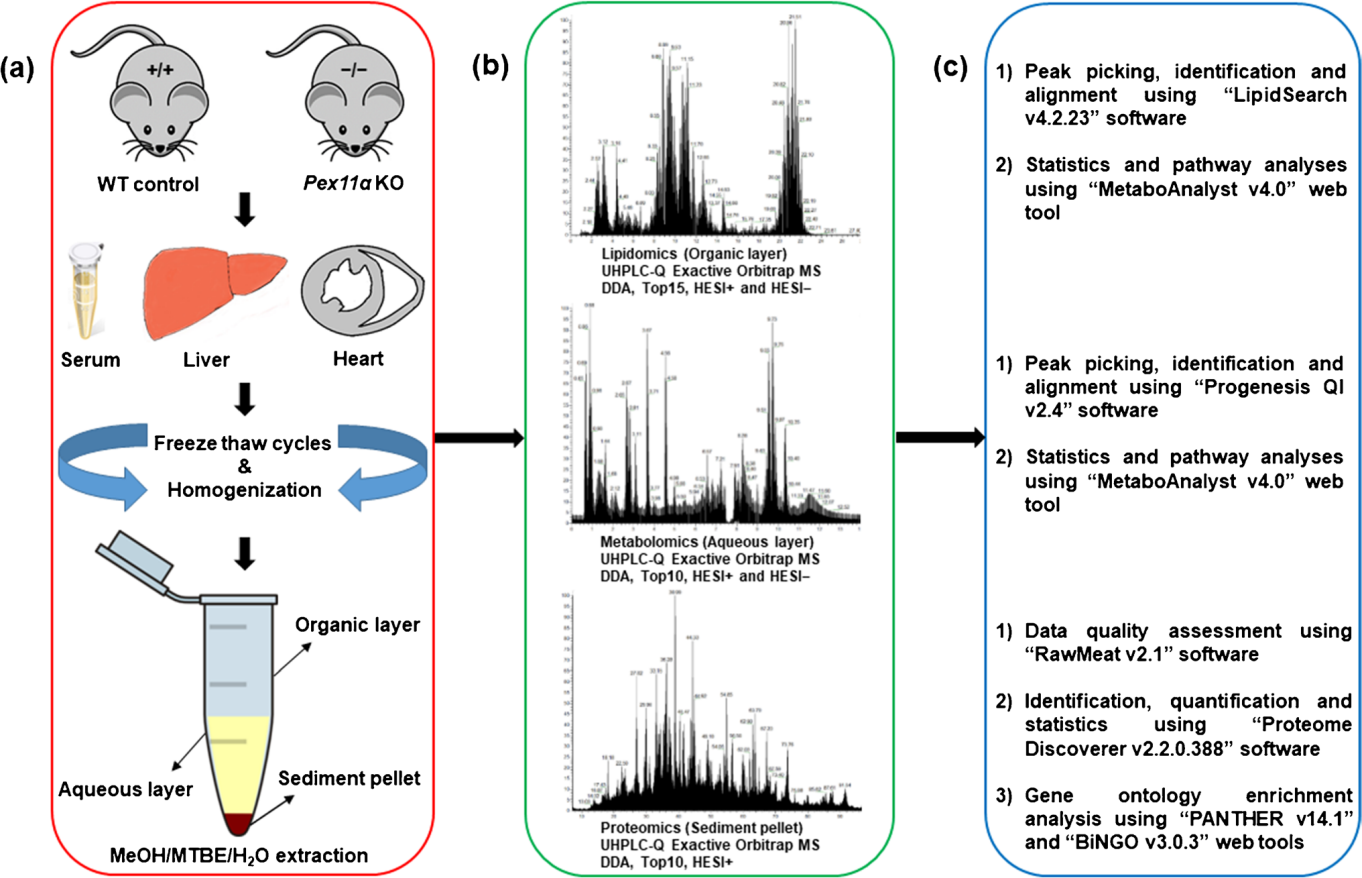
Schematic representation of the experimental workflow for sequential omics analyses of serum, liver, and heart tissue specimens from wild type (WT; *Pex11α*^+/+^) control and *Pex11α* knockout (KO; *Pex11α*^−/−^) mice. **a** Sample collection, homogenization, and simultaneous extraction of lipids (organic layer), polar metabolites (aqueous layer), and proteins (sediment pellet) using MeOH/MTBE/H_2_O liquid–liquid extraction. **b** Analytical workflow of multiple-molecular omics (lipidomics, metabolomics, and proteomics) data acquisition independently in positive- and negative-ion mode using ultra-high-performance liquid chromatography coupled with high-resolution tandem mass spectrometry (UHPLC-HRMS/MS). **c** Data processing, compound identification, statistics, and pathway analysis using dedicated bioinformatics tools

First, we optimized an integrated workflow that can profile a broad spectrum of biomolecules (lipids, polar metabolites, and proteins) from a single piece of tissue and/or biological fluids (Fig. 1). Briefly, an ice-cold MeOH/MTBE/H_2_O liquid–liquid extraction protocol was utilized for the concurrent extraction of lipids (upper organic layer), polar metabolites (lower aqueous layer), and proteins (lower precipitated pellet) [33, 34]. Extraction was carried out in glass vials in order to minimize the sample loss and contaminants caused by the interaction between organic solvents and plastic tubing [35]. After extraction, distinct fractions were collected and processed independently (detailed in “2” section), after which they were subjected to individual well-established omics analyses using UHPLC-HRMS/MS system. Specialized software tools were then used for identification, relative-quantification, and statistical analysis of lipids, polar metabolites, and proteins (Fig. 1). Taken together, over 93% of the identified lipids, polar metabolites, and proteins showed high technical reproducibility (area relative standard deviation values were less than 15%) across all replicates and tested biological specimens (ESM Fig. S3).

### Global lipidomics analysis

#### Comprehensive lipidome coverage

Lipids can be identified in positive- and/or negative-ion mode based on their chemical complexity. For instance, the majority of the glycerophospholipids (GP) and sphingolipids (SP) are identified in both ionization modes with various charge carriers, whereas neutral lipids such as monoglyceride (MG), diglyceride (DG), triglyceride (TG), coenzyme (Co), cholesterol, fatty acyl esters of cholesterol (ChE), and carnitine (AcCa) are predominantly observed in positive-ion mode [36, 37]. With the aim of representing broader lipidome coverage, data were acquired independently in positive- and negative-ion mode (three biological replicates in technical triplicates). Base peak chromatograms obtained with serum, liver, and heart tissue homogenate lipid extracts unveiled a clear peak separation and characteristic lipid ion profiles in both positive- and negative-ion mode (ESM Fig. S4).

LipidSearch (LS) software, which includes a database of greater than 1.7 million lipids and corresponding predicted fragment ions, was used for the identification and alignment of lipid molecular species based on precursor ion accurate mass and characteristic fragment ion patterns [22]. After identification, positive- and negative-ion mode search results were aligned within a time window (0.25 min) and merged into a single report. On average, a total of 3000 lipid ion species belonging to several lipid classes were identified from mice serum (2867), liver (3808), and heart (3518) tissue homogenates (ESM Dataset S1, total count).

Even though lipids were identified based on accurate mass (≤ 5 ppm) and fragment ions (≤ 5 ppm), the aligned-results report comprised a huge number of false positives due to various reasons including fragment mismatch, peak tailing, and poor integration of the chromatographic peak. In order to remove/minimize false positives and to provide more confident lipid identifications, a series of pre-defined filtering criteria were applied (ESM Table S4). For instance, (i) Rejection (Rej parameter calculated by LS based on signal to noise ratio, intensity ratio, and number of data points thresholds of each peak group) equal to false (ESM Dataset S1, filtered count_1), (ii) deletion of low confidence lipid assignments on the basis of fragmentation grade, removal of recurring lipid annotations by selection of main ion adduct from multiple charge carriers (ESM Data file S1 and Dataset S1, filtered count_2), and (iii) manual curation (ESM Dataset S1, filtered count_3). Throughout the manual curation, peak quality, fragmentation match score, area relative standard deviation, mass accuracy, isotopic profile, base retention time (as shown in ESM Fig. S4), and chromatographic peak integration for each precursor ion were carefully examined. Furthermore, a few ambiguous lipid assignments were ignored for final quantitation, which were possibly artifacts resulting from in-source fragmentation of corresponding precursor lipids. For instance, across all the identified samples, lysodimethylphosphatidylethanolamine (LdMePE) and dimethylphosphatidylethanolamine (dMePE) lipid species share the same retention time and approximately equivalent abundance ratio values with the identical acyl-chain compositions of LPC and PC lipid species, respectively (ESM Data file S1) [38]. Furthermore, low confidence free fatty acids were not considered by the LS software, due to the fact that the high confidence level identification with a diagnostic fragment ion ([M − H-44]^−^, resulting from a loss of CO_2_) requires relatively high collisional energies (NCE ˃ 40) [39]. Moreover, OAcyl(gamma-hydroxy) fatty acids (OAHFA), simple Glc series (CerG2GNAc), phosphatidylethanol (PEt), and phosphatidylmethanol (PMe) lipid species were also ignored for final quantitation due to insufficient diagnostic fragment ions for confident lipid identification.

**Fig. 2:**
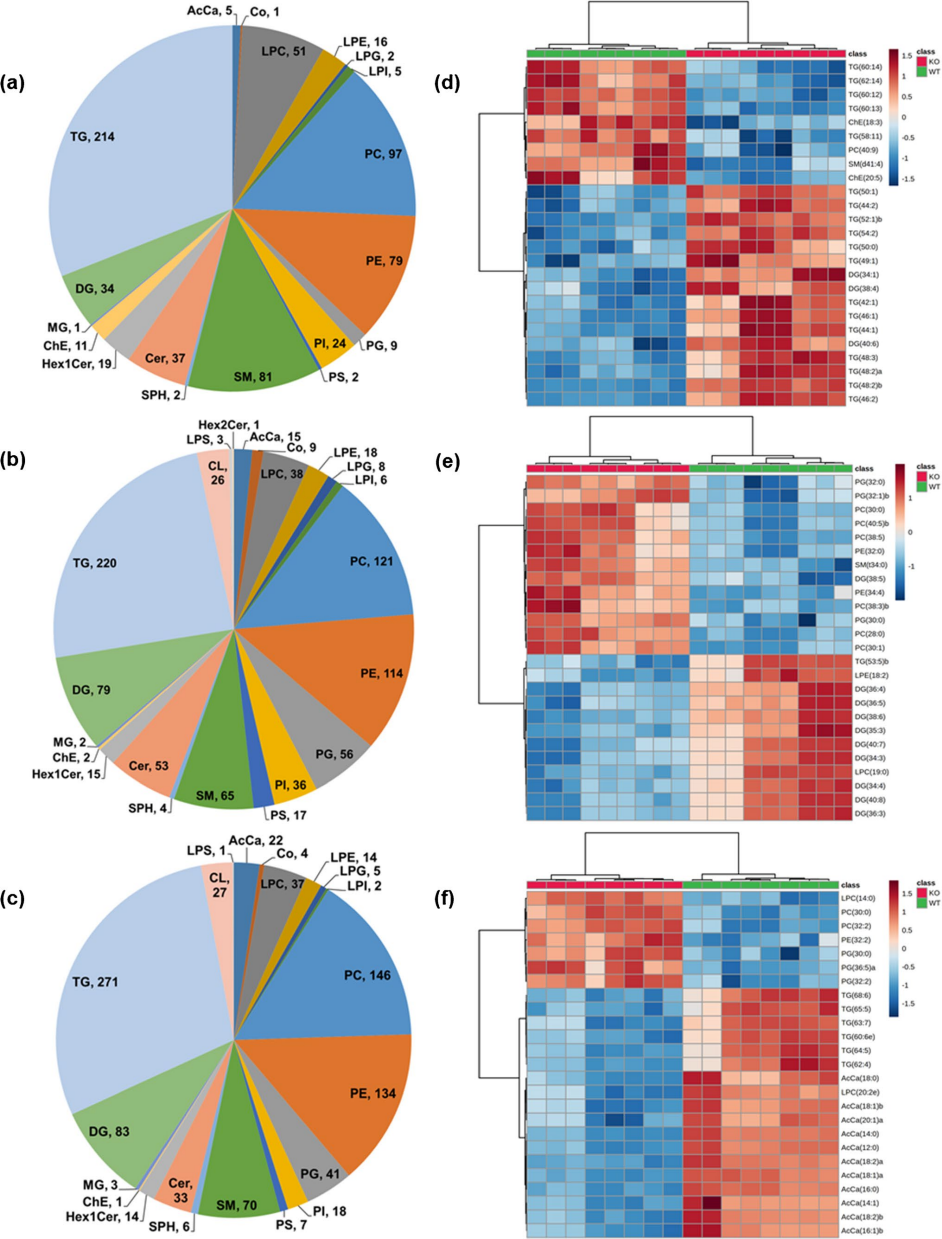
Overview of the global lipidomics analysis. Pie charts depict the summary as well as distribution patterns of identified lipid species/classes among all the tested biological specimens **a** serum, **b** liver, and **c** heart tissue homogenates. The numbers in pie charts represent the number of lipid species confidently identified and quantified in a particular lipid class. Heatmap illustration of lipid species between *Pex11α* knockout (KO; *Pex11α*^−/−^) and wild type (WT; *Pex11α*^+/+^) control mouse **d** serum, **e** liver, and **f** heart tissue homogenates. Data in heatmaps is based on *z*-scores for the normalized, transformed, and scaled data. The top 25 differentially abundant lipid species (with lowest FDR-adjusted *p*-values) were ranked based on *t*-test, distance was measured using the Euclidean correlation, and clustering was performed using the Ward algorithm

After careful data evaluation and manual curation, a total of 690 (serum), 908 (liver), and 939 (heart) distinct lipid species were retained as unambiguous identifications (ESM Dataset S1, final count). Figure 2a–c summarizes an overview and distribution patterns of the identified unique lipid species, covering 22 different lipid classes belonging to three major lipid categories including glycerophospholipids (PC, LPC, PE, LPE, PG, LPG, PI, LPI, PS, LPS, and CL), glycerolipids (MG, DG, and TG), sphingolipids (SM, SPH, Cer, Hex1Cer, and Hex2Cer), and others (AcCa, ChE, and Co). In general, TGs were the most prominent class of lipid species identified among all the tested biological specimens (214, 220, and 271 TG species in serum, liver, and heart tissue homogenates, respectively), followed by PC (97 in serum, 121 in liver, and 146 in heart) and PE (79 in serum, 114 liver, and 134 in heart). A more detailed look into the distribution patterns of the lipid classes revealed (i) a reasonably high number of LPC, SM, and ChE, (ii) relatively lower number of AcCa, PC, PE, PG, PS, and DG, and (iii) no cardiolipin (CL) and LPS lipid species were identified in serum samples in comparison to the liver and heart tissue homogenates (Fig. 2a–c). Complete lipidomic dataset (final list of identified/aligned lipid species, their relative abundance values, differentially abundant lipids, and the associated statistical significance values) in a format that follows “LIPID MAPS consortium” and the “Lipidomics Standards Initiative” minimum reporting guidelines is provided as supplementary Microsoft Excel worksheets (ESM Dataset S1).

#### Relative-quantitative lipidomics

The generated global lipidome data were statistically evaluated using fold-change analysis, *t*-test, volcano plots, principal component analysis (PCA), and hierarchical clustering analysis (HCA; including dendrogram and heatmaps), to distinguish between the groups. As seen in ESM Fig. S6a-b, a clear separation (occupation of different space in the PCA score plots) and a high degree of dissimilarity (dendrogram) were observed in all the tested biological specimens of *Pex11α* KO and WT control mice. This is eventually visualized with two-dimensional hierarchical clustering heatmaps (Fig. 2d–f), where each column represents a biological sample and each row represents a significant lipid molecular species, ranked based on *t*-test. Furthermore, descriptive volcano plot analysis of all measured lipids revealed significant (FDR-adjusted *p*-value ≤ 0.05) changes in the abundance (ratio threshold ± 2) of 102 lipid species (64 increased, 38 decreased) in serum, of 145 lipid species (80 increased, 65 decreased) in liver, and 102 lipid species (12 increased, 90 decreased) in heart tissue homogenates of *Pex11α* KO mice, in comparison to those of WT controls (ESM Fig. S6c and Dataset S1_Quan). Moreover, we calculated and compared the individual lipid class levels (sum of the normalized relative abundance values of all analyzed lipid species within the class) among WT control and *Pex11α* KO mice (ESM Fig. S5a-c and Dataset S1_Lipid class). The various lipid classes and the individual lipid molecular species that were differentially abundant are discussed below.

Sphingolipids (SP) are an essential class of bioactive lipids with a sphingoid backbone. They are present as underivatized (e.g., sphingosine), or N-acetylated with fatty acids (e.g., Cer), or further derivatized by addition of charged, neutral, phosphorylated, and glycosylated head groups to form more complex SP (e.g., SM, CerP, CerPE, HexCer, and GalCer). Depending on the fatty acid conjugates and concentration, they play vital roles in proliferation, differentiation, apoptosis, senescence, and autophagy [40]. In the current study, numerous sphingolipid species (139 in serum, 138 in liver, and 123 in heart) covering SM, Cer, Hex1Cer, Hex2Cer, and SPH lipid classes were documented (Fig. 2a-c, ESM Dataset S1). Within the SP category, several ceramide and cerebroside (e.g., hexosylceramide) lipid species were markedly increased in serum (seven Cer, five Hex1Cer species) and liver (nine Cer, five Hex1Cer species) tissue homogenates of *Pex11α* KO mouse. Among them, two lipid species, namely Hex1Cer(d36:1; d18:1_18:0) and Hex1Cer(d43:2; d18:1_25:1), were found to be common and raised in both the liver tissue and serum. Similarly, 12 and one SM lipid species exhibited higher levels in *Pex11α* KO mice liver and serum (while three species decreased) in comparison to the WT controls. In contrast, we did not notice statistically significant differences in the individual species and the total sphingolipid class levels of *Pex11α* KO mouse heart tissues (ESM Dataset S1 and Fig. S5). Pettus et al. observed increased levels of specific Cer and SM lipid species in brain and fibroblasts of newborn mice lacking *Pex5*, as well as in the fibroblasts of D‐specific multifunctional protein 2 (MFP2) deficient mice [41]. Furthermore, similar observations were witnessed in fibroblasts derived from X-linked adrenoleukodystrophy (X-ALD) patients and proposed perturbations in C_26:1/0_-ceramide lipid and ratio of C_26:1/0_-ceramide/C_22:0_-ceramide as possible potential diagnostic markers to study the peroxisomal disorders [41]. Likewise, other studies reported higher amounts of SM lipids with long-chain fatty acid moieties in liver [42] and cultured skin fibroblasts [43, 44], SM lipids with short-chain fatty acid moieties in cerebellum [42], and ceramide monohexoside in the cerebral gray matter but not in the white matter of ZS patients (e.g., *Pex1* and *Pex26* mutations) [45]. Moreover, increased levels of ceramide monohexoside were also observed in *Pex5*-mutated Chinese hamster ovary cells [46]. Remarkably, a recent metabolomic study disclosed unanticipated decreased levels of multiple SM lipid species in ZSD patient’s plasma [47].

Glycerophospholipids (GP) are structural integral components of most cell and organelle membranes, which play crucial roles in various physiological and pathological processes. Within the GP category, we identified several lipid species (285 in serum, 443 in liver, and 432 in heart) spanning 11 different lipid classes (PC, LPC, PE, LPE, PG, LPG, PI, LPI, PS, LPS, and CL), which unveiled a wide diversity of quantitative trends between the tested biological specimens (Fig. 2, ESM Dataset S1 and Fig. S5). In detail, among phosphatidylcholines (the most abundant lipid class in GP), three PC species (PC 30:0, 30:1, and 37:5) in serum, seven species (PC 28:0, 30:0, 30:1, 38:3, 38:5, 40:5, and 42:9) in liver, and four species (PC 30:0, 32:2, 41:2, and 42:9) in heart were significantly increased in *Pex11α* KO mice. Among these, PC(30:0; 16:0_14:0) lipid showed higher levels in all the tested biological specimens of *Pex11α* KO mice, whereas PC(30:1; 16:1_14:0) and PC(42:9; 22:5_20:4) lipid species exhibited a similar rise in serum and liver, heart and liver tissue homogenates, respectively. Only one lipid species (PC 36:5) in liver, two lipid species including one ether-linked species (PC 36:6e, 41:5) in heart, and more interestingly five species (PC 40:9, 41:6, 42:6, 42:11, and 44:12) in serum, all comprising poly-unsaturated fatty acids (PUFA; C22:6) were significantly decreased in the *Pex11α* deficient mice. LPC species with a single fatty acyl chain (C ≤ 20), derived from PC lipids, displayed comparatively lower levels in *Pex11α* deficient animals (LPC 19:0, 20:0, 20:2e in serum, LPC 19:0, 19:1, 20:1, 20:2 in liver, and LPC 20:2e in heart). In contrast, LPC 14:0 showed slightly higher levels in *Pex11α* KO mouse heart tissue homogenates. In phosphatidylethanolamine lipid class (the second-most abundant in GP), two species (PE 38:5, 40:4) in serum, one species (PE 32:2) in heart, and six lipid species (PE 32:0, 34:4, 38:4, 38:6e, 40:5p, 40:6e) in liver were significantly increased in *Pex11α* KO mice. Similar to PC, PE 36:5 and PUFA containing two species (PE 41:6, 42:8) were significantly decreased in *Pex11α* KO mouse liver. LysoPE lipids in the liver (eight species, ranging from LPE 18:2 to 22:6) followed the same pattern as of LPC but remained relatively constant in serum and heart. A number of PG lipid species (starting from PG 30:0 to 44:10) showed elevated levels in *Pex11α* KO mouse liver (nine species) and heart (four species) tissue homogenates. Among these, PG (30:0; 16:0_14:0) and PG (44:10; 22:5_22:5) lipids were found to be common and raised in both the liver and heart tissue homogenates. Nonetheless, individual PG lipid species in serum and total PG and LPG lipid species/class levels in all the tested biological specimens were not significantly changed. In addition to these phospholipid class alterations, we noted significantly increased levels of PI (16 species, starting from PI 34:1 to 40:7) and PS (four species including PS 34:2, 36:1, 38:4, and 42:7) lipid species in *Pex11α* KO mouse liver. On the contrary, three (PI 38:3, 40:4, and 40:5) and two (PI 39:4, 40:6) PI lipid species were decreased in serum and heart tissue homogenates. The remaining glycerophospholipid classes and their individual lipid molecular species did not show major differences in *Pex11α* deficient animals.

Cardiolipin (CL) is a dimeric phospholipid, which makes up to 20% (inner) and 3% (outer) of the total mitochondrial membrane lipid composition. While in peroxisomes, CL makes up to 2–4% of the total phospholipid pool [48]. In the present study, CL lipid species were identified in both liver (26 species) and heart (27 species) tissue homogenates, but not in serum (Fig. 2, ESM Dataset S1). Among these, five CL lipid species (CL 74:9, 76:12, 76:12, 78:14, and 78:15) were significantly elevated in *Pex11α* KO mouse liver, possibly due to mitochondrial proliferation [9, 10, 49]. Noticeably, the individual species and the total CL levels remained relatively unchanged in the heart tissue homogenates. Abnormalities in the CL composition are known to be associated with severe metabolic disorders (e.g., Tangier disease and Barth syndrome) and are also linked with various pathological states [50]. In this regard, it is worth mentioning that decreased levels of several CL species were observed in the cultured skin fibroblasts derived from single PEDs and ZSDs patients [44, 51]. Nevertheless, the precise molecular mechanisms behind these phospholipid class (CL) alterations (positive/negative) in relation to peroxisomes and peroxisomal disorders are not clear yet, requiring further studies in this regard.

In the glycerolipid (GL) category, we spotted several lipid species (249 in serum, 301 in liver, and 357 in heart) belonging to MG, DG, and TG lipid classes (Fig. 2, ESM Dataset S1). In serum, 11 different lipid species ranging from DG 32:0 to 40:6 showed significantly higher levels in *Pex11α* KO mice. In contrast, DG lipid class levels and a wide variety of individual lipid species (starting from DG 32:3 to 44:9) including several PUFA containing species (for instance, DG 40:7, 40:8, 42:6, 42:7, 42:9, 42:10, and 44:7) were significantly decreased (except DG 38:5) in *Pex11α* KO mouse liver. However, no statistically significant changes were noted in heart tissues. TG lipids consist of a glycerol backbone esterified with three fatty acids. They are a vital source of energy during cellular metabolism. Among these, 55 TG lipid species (34 increased, 21 decreased) in serum, 14 species (10 decreased, four increased) in liver, and 65 lipid species (three increased, 62 decreased) in heart showed statistically significant differences in *Pex11α* KO mice when compared to the WT controls. Among the differentially abundant TGs, TG(60:13; 20:5_18:2_22:6) lipid showed reduced levels in all the tested biological specimens of *Pex11α* KO mice. Likewise, increased levels of TG(44:1; 16:1_14:0_14:0), and decreased levels of TG(56:10; 18:3_18:2_20:5) and TG(58:11; 18:3_18:2_22:6) lipid species were noted in the liver tissue and serum, whereas lower levels of TG(62:14; 18:2_22:6_22:6) lipid species were observed commonly in serum and heart tissue homogenates. Overall, the majority of the ether-linked and PUFA containing TG lipid species were significantly decreased in *Pex11α* KO mice (ESM Dataset S1). A minimal number of MG lipid species were identified in serum (one species), heart (three species), and liver (two species) tissue homogenates and they were statistically not as robust as the lipid classes with higher total numbers of species.

Notably, as stated earlier, a number of GP and GL lipid species containing long-chain fatty acid and poly-unsaturated fatty acid moieties showed significant variances (both positive and negative) in the biological specimens of *Pex11α* KO mice. Interestingly, fragmentation data (MS/MS) revealed that the majority of the lipid species with docosahexaenoic acid (DHA, C22:6) moieties were decreased (with few exceptions) in *Pex11α* deficient mice when compared to those of WT controls (ESM Dataset S1). DHA is an essential poly-unsaturated fatty acid, synthesized from the dietary fatty acid α-linoleic acid (ALA, C18:3) by successive elongation and desaturation reactions in the endoplasmic reticulum followed by peroxisomal β-oxidation [52]. Several studies have documented reduced levels of DHA and DHA-containing lipid species in different peroxisomal diseases [51–56]. Moreover, a previous study suggests that DHA enhances the peroxisomal division in a microtubule-independent manner [55].

In addition to the differences in SP, GP, and GL classes and specific lipid species, other lipid classes including fatty acyl esters of carnitine (AcCa), free and esterified cholesterol (ChE) species, and coenzyme (Co) were confidently identified and relatively quantified in all the tested biological specimens (Fig. 2, ESM Dataset S1). Concisely, among acylcarnitines (AcCa), AcCa 6:0 in serum and AcCa 10:0 in liver showed an increment, while the majority of the measured AcCa species were reduced in *Pex11α* KO mouse heart tissues. Two cholesteryl ester species (ChE 18:3, 20:5) showed relatively lower levels in *Pex11α* KO mouse serum.

Several studies employed both targeted and untargeted lipidomics approaches to study the functional consequences and to explore novel molecular biomarkers for a better understanding and diagnosis of peroxisomal disorders. To mention a few, differential lipidomic analyses were carried out in (i) cultured primary skin fibroblasts derived from PBDs (e.g., *Pex1*, *Pex13*, *Pex16*, and *Pex7* mutations) and single PEDs (e.g., ABCD1, ACOX1, DBP, and ACBD5 deficiencies) patients [43, 44, 51, 56–58], (ii) brain, liver, and fibroblasts from ZSDs patients (e.g., *Pex1*, *Pex26*, and *Pex5* mutations) [42], (iii) plasma from ZSDs (e.g., *Pex1* mutation), AMACR and DBP deficiency, Refsum disease, and RCDP type 1 or 5 patients [47, 59], (iv) brain, liver, kidney, and retina of peroxisomal disorder (e.g., ZS, NALD, X-ALD, BED, and AMN) patients [54], and also in mouse and cell culture models of several peroxisomal dysfunctions [41, 46, 56, 60]. The diversity and specimen-specific alterations in distinct lipid profiles reflect the heterogeneity and the widespread lipid-associated metabolic functions of peroxisomes in different cell types, organs, and whole organisms.

### Global metabolomics analysis

Further, we investigated the polar metabolic changes and metabolic pathways modulated in different biological specimens due to *Pex11α* deficiency. To attain this, the lower aqueous phase containing polar metabolites was processed, untargeted metabolomic analysis was carried out (three biological replicates in technical triplicates), and obtained data were evaluated using the Progenesis QI. On average, global metabolomics data generated over 10,000 features across all the tested biological specimens. Putative identification of metabolites was performed for the ions that had MS/MS data. Furthermore, compounds were manually filtered, based on their coefficient of variation, duplicates were removed, and misidentifications based on retention time, quality of peak shape, score, fragmentation match score, isotope similarity, and mass accuracy were deleted. After removal of false positives, by combining both positive- and negative-ion modes data collectively, 123, 98, and 80 metabolites were tentatively identified in serum, liver, and heart tissue homogenates, respectively. The identities of the metabolites (putatively annotated metabolites, their relative abundance values, differential abundances, and the associated statistical significance values) are provided as supplementary Microsoft Excel worksheets (ESM Dataset S2). Metabolites covering a broad spectrum of chemical classes, including amino acids, nucleotides, peptides, carbohydrates, bile acids, secondary lipid mediators, polyamines, vitamins, and cofactors, were identified. The majority of them were found to be shared between all the tested biological specimens.

**Fig. 3:**
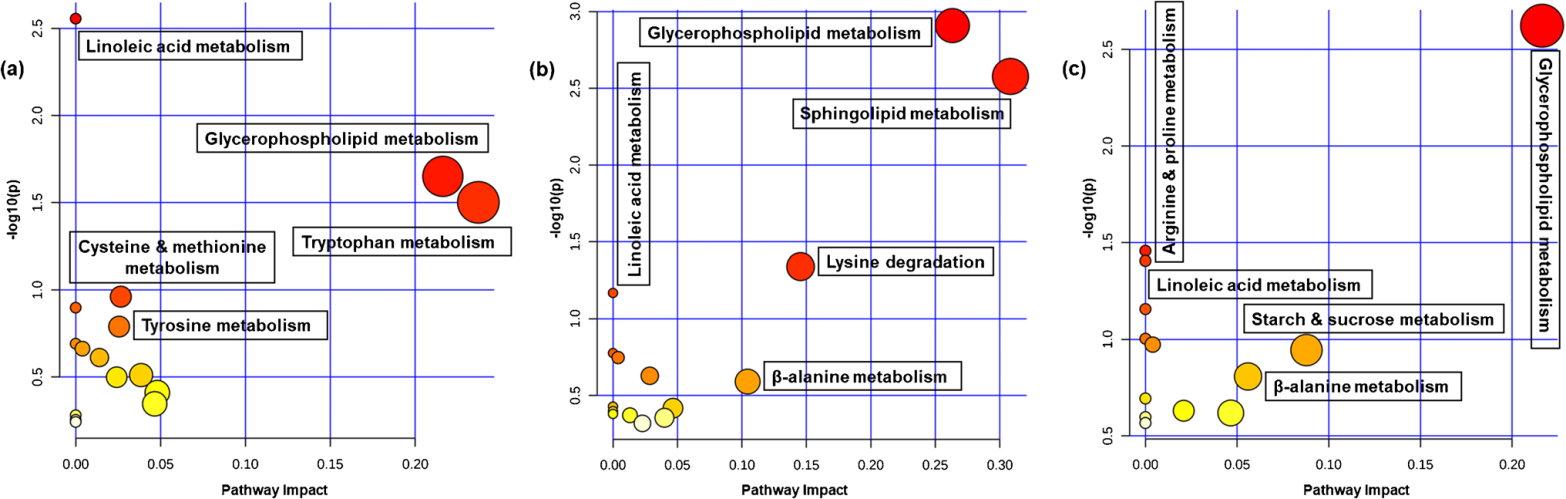
Scatterplots of enriched KEGG pathways for combined lipidomic/metabolomic experiments when comparing *Pex11α* knockout (KO; *Pex11α*^−/−^) with wild type (WT; *Pex11α*^+/+^) control mice. Pathway topology analysis depicting the dysregulated metabolic pathways in **a** serum, **b** liver, and **c** heart tissue homogenates, respectively. Here, the *x*-axis represents the pathway impact, and the *y*-axis represents the pathway enrichment. Each node marks a pathway. The size (pathway impact value) and color (-log10 *p*-value) of the nodes represent the number of lipid/polar metabolite species and level of significance, respectively

Using PCA, HCA, and descriptive univariate statistical analysis, we observed a clear separation and significant changes between the *Pex11α* KO and WT control groups (ESM Fig. S7 and Dataset S2). To explore the metabolic pathways that were dysregulated in the *Pex11α* KO mice, differentially abundant polar metabolites (25 in serum, 12 in liver, 11 in heart) and lipid species (102 in serum, 145 in liver, 102 in heart) were combined (of these, collectively 82, 114, and 52 compounds of serum, liver, and heart were found in the HMDB database), and metabolic pathways analyses (KEGG, *Mus musculus* libraries) were performed using the MetPA module of MetaboAnalyst. Several pathways related to lipid metabolism (e.g., glycerophospholipid, sphingolipid, glycerolipid, linolenic acid, α-linolenic acid and arachidonic acid metabolism, and fatty acid degradation), amino acid metabolism (e.g., tryptophan, purine, tyrosine, cysteine and methionine metabolism, and lysine degradation), nucleotide metabolism, carbohydrate metabolism, and other metabolic pathways were found to be altered in *Pex11α* KO mice. The summary of pathway analysis is shown in Fig. 3 and is listed in detail in ESM Table S12. Many research articles can be found, focusing on alterations in lipid species, and the role of lipid metabolism is well established in several peroxisomal disorders. Nevertheless, a comprehensive polar metabolomic map and the metabolic pathways that are altered in various peroxisomal disorders (for instance with peroxisomal biogenesis gene mutants or single peroxisomal enzyme mutants) are still poorly understood. Recently, Wangler et al. performed untargeted metabolomics analysis and revealed alterations in known lipid pathways and unanticipated changes in carbohydrate metabolic processes including starch and sucrose metabolism, glycolysis, glycogen catabolism, and pentose phosphate pathway in *Pex16* and *Pex2* mutant *Drosophila melanogaster* (fruit flies) and *Pex5* deficient mouse liver [61].

### Label-free quantitative proteomics analysis

Proteins play crucial roles in various cellular functions, and proteomics has emerged as a powerful tool to identify both pathophysiological mechanisms as well as potential biomarkers for various diseases. In order to identify alterations in the *Pex11α* KO mice, tissue protein relative abundance values were compared to those of WT control mice by relative-quantitative proteomics. At this level of the experiment, the remaining lower sediment pellets (possibly containing stable and highly abundant proteins) were re-solubilized, and proteins extracted, digested with trypsin, and analyzed individually (three biological replicates in technical triplicates) by shotgun label-free quantitative (LFQ) proteomics. Prior to the quantitative proteome data analysis, data quality attributes (e.g., charge distributions and scan rates) and suitability of the sample complexity and applied method (e.g., Full MS/ddms^2^, Top10) were inspected visually using RawMeat (v2.1, Vast Scientific) software (ESM Data file S2).

**Fig. 4:**
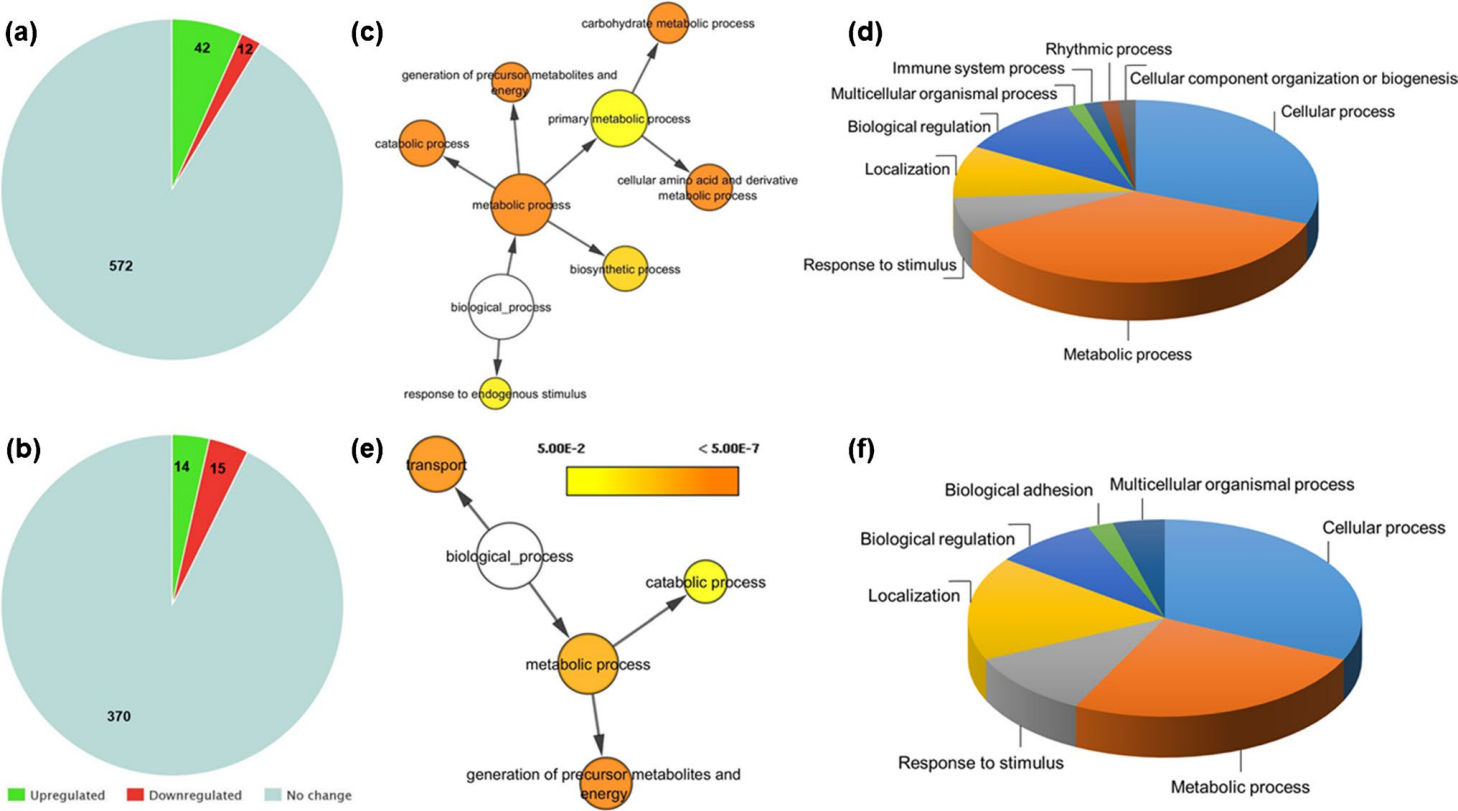
Label-free quantitative proteomic analysis of *Pex11α* knockout (KO; *Pex11α*^−/−^) and wild type (WT; *Pex11α*^+/+^) control mice. Pie charts depict the number of quantified and differentially abundant proteins in **a** liver and **b** heart tissue homogenates. Biological processes of the differentially abundant proteins in (**c** and **d**) liver and (**e** and **f**) heart analyzed by Cytoscape and PANTHER. Color scale indicates the significance, and size of the circle indicates the number of proteins involved

In total, the Proteome Discoverer software with Sequest HT search algorithm identified 1064 and 738 proteins from the sediment pellets of the liver and the heart tissue homogenates, respectively (ESM Dataset S3). Out of these, 626 and 399 master proteins in the liver and heart were quantified with two or more peptides and a strict target false discovery rate value of 0.01 (1% FDR). The corresponding list of identified and quantified proteins, as well as the data analysis output file including normalized abundance values, abundance ratios, and the corresponding statistical information, is presented as supplementary Microsoft Excel worksheets (ESM Dataset S3). Two-dimensional PCA score plots of the quantified proteome (normalized, log-transformed, and Pareto-scaled) showed that all the replicate acquisitions are clustered together and revealed a clear separation illustrating significant changes in the tissue proteome of *Pex11α* KO compared to WT controls (ESM Data file S2). In detail, the LFQ proteomics approach revealed significant (*p*-value threshold ≤ 0.05) changes in the abundance (ratio threshold ± 2) of 54 proteins (42 increased, 12 decreased) in the liver and 29 proteins (14 increased, 15 decreased) in the heart tissues of *Pex11α* KO in comparison to the WT control mouse (Fig. 4a-b, ESM Dataset S3). Interestingly, we noticed a downregulation of NUDT7 protein (peroxisomal coenzyme A diphosphatase) in the liver. NUDT7 eliminates toxic nucleotides (e.g., oxidized CoA) and nucleotide diphosphates (e.g., ADP-ribose, NAD + , and NADH) and regulates CoA and acyl-CoA levels in the peroxisomes in response to metabolic needs [62, 63]. Moreover, dysregulation of NUDT7 was also observed during senescence [64], and knockdown of NUDT7 resulted in the disruption of lipid homeostasis in chondrocytes [63]. In the heart, the small GTPase proteins from the Ras superfamily, which includes transforming protein RhoA, Ras-related protein Rap-1A, and Ras-related protein Rab-15 which regulates cytoskeletal reorganization, cell proliferation, and is associated with the peroxisomal membrane dynamics and biogenesis, were found to be downregulated in *Pex11α* KO [65, 66]. Likewise, other proteins including acyl-CoA-binding protein, fatty acid synthase, isocitrate dehydrogenase (NADP) cytoplasmic, isocitrate dehydrogenase (NADP) mitochondrial, aldehyde dehydrogenase, carbonyl reductase, glutathione S-transferase, enoyl-CoA delta isomerase 2, glutathione peroxidase, and farnesyl pyrophosphate synthase, among other proteins, were differentially abundant in *Pex11α* KO in comparison to WT control mouse tissues. Seemingly, the relatively low number of total protein identifications from the whole-tissue homogenates (in comparison with the conventional proteomics workflow) might be ascribed to the one-step liquid–liquid extraction procedure (traditionally used for lipidomics) optimized for serial-omics analyses, from a single piece of biological sample. Also, the non-appearance of several important peroxisomal membrane proteins, matrix proteins, and peroxins might be ascribed to their low abundance and to the well-known difficulties associated with the extraction of labile hydrophobic proteins.

Furthermore, to elucidate the biological context of the differentially abundant proteins (DAPs), gene ontology enrichment analysis was carried out using PANTHER and Cytoscape (BiNGO plug-in) bioinformatics tools. Biological information such as molecular functions, biological processes, cellular components, signalling pathways involved, and protein classes of the DAPs was obtained as shown in Fig. 4c-f and ESM Data file S3. Taken together, in liver and heart tissues, we found that DAPs were involved in translation, protein folding, amino acid metabolism, nucleotide metabolism, fatty acid processing, carbohydrate metabolism, steroid biosynthesis, electron transport chain, and ROS metabolism (Fig. 4c-f, ESM Data file S3). Lastly, it is worth noting that many of these proteins (DAPs) are located within the mitochondrion, which suggests that knockout of *Pex11α* may induce major changes to mitochondrial metabolism.

## Conclusion and future perspectives

In the present study, we utilized high-throughput mass spectrometry-guided sequential omics workflow and performed comprehensive semi-quantitative lipidomic, metabolomic, and proteomic analyses of different biological specimens (serum, liver, and heart) from *Pex11α* KO adult male animals. Complex lipids, polar metabolites, and proteins were simultaneously extracted via a single-step liquid–liquid (MeOH/MTBE/H_2_O) extraction method, and the corresponding omics datasets were acquired using ultra-high-performance liquid chromatography coupled with high-resolution tandem mass spectrometry and processed with LipidSearch, Progenesis QI, and Proteome Discoverer, respectively. Primarily, we identified characteristic changes in the composition of several lipid species/classes, polar metabolites, metabolic pathways, and protein profiles of serum, liver, and heart tissue homogenates of *Pex11α* KO mice in comparison to the WT controls. Nevertheless, there are certain limitations in the current study that need to be mentioned. First, serial-omics analyses of distinct biological specimens were performed with a small sample size (three biological replicates in technical triplicates). Although stringent data analysis criteria were employed, a profound statistical evaluation is restricted due to the limited number of biological replicates, thereby, false positives and/or negatives may not be overruled at this stage. Complementary studies with an independent large sample size may be warranted to elucidate the panel of molecular signatures that were presented in this study and to demonstrate their potential as diagnostic biomarkers. Second, due to medium number of protein identifications, the present study did not allow us to integrate multi-layer omics data to fully capture and represent the complete molecular pathway changes occurring due to *Pex11α* deficiency. Despite these limitations, to the best of our knowledge, this is the first comprehensive serial-omics study to report the molecular alterations occurring in different biological specimens of *Pex11α* deficient mice. Further studies addressing changes in other biological specimens (e.g., fibroblasts, brain, kidney, and testis), the influence of diet (e.g., high-fat diet and DHA supplementation) on molecular alterations, and integrated analysis of multi-omics data with large cohort studies need to be investigated in the near future to gain deeper insights of *Pex11α* molecular functions and to uncover the pathophysiological mechanisms of peroxisomal disorders.

## Supplementary Information

Below is the link to the electronic supplementary material.

Supplementary file1 (PDF 2072 kb)

Supplementary file2 (PDF 7935 kb)

Supplementary file3 (XLSX 1081 kb)

Supplementary file4 (XLSX 110 kb)

Supplementary file5 (XLSX 400 kb)

Supplementary file6 (PDF 637 kb)

Supplementary file7 (PDF 605 kb)

## Data Availability

All relevant data and materials are within the manuscript and its electronic supplementary material. Raw MS data files have been deposited to the EMBL-EBI MetaboLights data respiratory with the unique identifier MTBLS1887.

## References

[1] Wanders RJ (2014). Metabolic functions of peroxisomes in health and disease. Biochimie.

[2] Schrader M, Fahimi HD (2006). Peroxisomes and oxidative stress. Biochim Biophys Acta.

[3] Baumgart E. Application of in situ hybridization, cytochemical and immunocytochemical techniques for the investigation of peroxisomes. A review including novel data. Robert Feulgen Prize Lecture 1997. Histochem Cell Biol. 1997;108(3):185–210. 10.1007/s004180050160. 10.1007/s004180050160 9342614

[4] Karnati S, Baumgart-Vogt E (2008). Peroxisomes in mouse and human lung: their involvement in pulmonary lipid metabolism. Histochem Cell Biol.

[5] Fidaleo M (2010). Peroxisomes and peroxisomal disorders: the main facts. Exp Toxicol Pathol.

[6] Fujiki Y, Okumoto K, Mukai S, Honsho M, Tamura S (2014). Peroxisome biogenesis in mammalian cells. Front Physiol.

[7] Waterham HR, Ferdinandusse S, Wanders RJ (2016). Human disorders of peroxisome metabolism and biogenesis. Biochim Biophys Acta.

[8] Braverman NE, D’Agostino MD, Maclean GE (2013). Peroxisome biogenesis disorders: biological, clinical and pathophysiological perspectives. Dev Disabil Res Rev.

[9] Li X, Baumgart E, Dong GX, Morrell JC, Jimenez-Sanchez G, Valle D (2002). PEX11alpha is required for peroxisome proliferation in response to 4-phenylbutyrate but is dispensable for peroxisome proliferator-activated receptor alpha-mediated peroxisome proliferation. Mol Cell Biol.

[10] Li X, Baumgart E, Morrell JC, Jimenez-Sanchez G, Valle D, Gould SJ (2002). PEX11 beta deficiency is lethal and impairs neuronal migration but does not abrogate peroxisome function. Mol Cell Biol.

[11] Colasante C, Chen J, Ahlemeyer B, Baumgart-Vogt E (2015). Peroxisomes in cardiomyocytes and the peroxisome / peroxisome proliferator-activated receptor-loop. Thromb Haemost.

[12] Anthonio EA, Brees C, Baumgart-Vogt E, Hongu T, Huybrechts SJ, Van Dijck P (2009). Small G proteins in peroxisome biogenesis: the potential involvement of ADP-ribosylation factor 6. BMC Cell Biol.

[13] Kobayashi S, Tanaka A, Fujiki Y (2007). Fis1, DLP1, and Pex11p coordinately regulate peroxisome morphogenesis. Exp Cell Res.

[14] Weng H, Ji X, Naito Y, Endo K, Ma X, Takahashi R (2013). Pex11alpha deficiency impairs peroxisome elongation and division and contributes to nonalcoholic fatty liver in mice. Am J Physiol Endocrinol Metab.

[15] Kopczynski D, Coman C, Zahedi RP, Lorenz K, Sickmann A, Ahrends R (2017). Multi-OMICS: a critical technical perspective on integrative lipidomics approaches. Biochim Biophys Acta Mol Cell Biol Lipids.

[16] Breitkopf SB, Taveira MO, Yuan M, Wulf GM, Asara JM (2017). Serial-omics of P53-/-, Brca1-/- mouse breast tumor and normal mammary gland. Sci Rep.

[17] Hendgen-Cotta UB, Esfeld S, Coman C, Ahrends R, Klein-Hitpass L, Flogel U (2017). A novel physiological role for cardiac myoglobin in lipid metabolism. Sci Rep.

[18] Nakayasu ES, Nicora CD, Sims AC, Burnum-Johnson KE, Kim YM, Kyle JE et al. MPLEx: a robust and universal protocol for single-sample integrative proteomic, metabolomic, and lipidomic analyses. mSystems. 2016;1(3). 10.1128/mSystems.00043-16. 10.1128/mSystems.00043-16 PMC5069757 27822525

[19] Salem MA, Juppner J, Bajdzienko K, Giavalisco P (2016). Protocol: a fast, comprehensive and reproducible one-step extraction method for the rapid preparation of polar and semi-polar metabolites, lipids, proteins, starch and cell wall polymers from a single sample. Plant Methods.

[20] Zhao C, Zhu L, Li R, Wang H, Cai Z (2019). Omics approach reveals metabolic disorders associated with the cytotoxicity of airborne particulate matter in human lung carcinoma cells. Environ Pollut.

[21] Li Z, Lai ZW, Christiano R, Gazos-Lopes F, Walther TC, Farese RV (2018). Global analyses of selective insulin resistance in hepatocytes caused by palmitate lipotoxicity. Mol Cell Proteomics.

[22] Breitkopf SB, Ricoult SJH, Yuan M, Xu Y, Peake DA, Manning BD et al. A relative quantitative positive/negative ion switching method for untargeted lipidomics via high resolution LC-MS/MS from any biological source. Metabolomics. 2017;13(3). 10.1007/s11306-016-1157-8. 10.1007/s11306-016-1157-8 PMC5421409 28496395

[23] Kumar Y, Dholakia BB, Panigrahi P, Kadoo NY, Giri AP, Gupta VS (2015). Metabolic profiling of chickpea-Fusarium interaction identifies differential modulation of disease resistance pathways. Phytochemistry.

[24] Korwar AM, Vannuruswamy G, Jagadeeshaprasad MG, Jayaramaiah RH, Bhat S, Regin BS (2015). Development of diagnostic fragment ion library for glycated peptides of human serum albumin: targeted quantification in prediabetic, diabetic, and microalbuminuria plasma by parallel reaction monitoring, SWATH, and MS^E^. Mol Cell Proteomics.

[25] Koch A, Schlemmer T, Höfle L, Werner B, Preußer C, Hardt M et al. Host-induced gene silencing involves transfer of dsRNA-derived siRNA via extracellular vesicles. bioRxiv. 2020:2020.02.12.945154. 10.1101/2020.02.12.945154.

[26] Chong J, Soufan O, Li C, Caraus I, Li S, Bourque G et al. MetaboAnalyst 4.0: towards more transparent and integrative metabolomics analysis. Nucleic Acids Res. 2018;46(W1):W486-W94. doi:10.1093/nar/gky310. 10.1093/nar/gky310 PMC6030889 29762782

[27] Jha P, McDevitt MT, Halilbasic E, Williams EG, Quiros PM, Gariani K et al. Genetic regulation of plasma lipid species and their association with metabolic phenotypes. Cell Syst. 2018;6(6):709–21 e6. 10.1016/j.cels.2018.05.009. 10.1016/j.cels.2018.05.009 PMC6397773 29909275

[28] Jha P, McDevitt MT, Gupta R, Quiros PM, Williams EG, Gariani K et al. Systems analyses reveal physiological roles and genetic regulators of liver lipid species. Cell Syst. 2018;6(6):722–33 e6. 10.1016/j.cels.2018.05.016. 10.1016/j.cels.2018.05.016 PMC6054463 29909277

[29] Xia J, Wishart DS (2010). MetPA: a web-based metabolomics tool for pathway analysis and visualization. Bioinformatics.

[30] Mi H, Muruganujan A, Huang X, Ebert D, Mills C, Guo X et al. Protocol update for large-scale genome and gene function analysis with the PANTHER classification system (v.14.0). Nat Protoc. 2019;14(3):703–21. 10.1038/s41596-019-0128-8. 10.1038/s41596-019-0128-8 PMC6519457 30804569

[31] Maere S, Heymans K, Kuiper M (2005). BiNGO: a Cytoscape plugin to assess overrepresentation of gene ontology categories in biological networks. Bioinformatics.

[32] Kazi RS, Banarjee RM, Deshmukh AB, Patil GV, Jagadeeshaprasad MG, Kulkarni MJ (2017). Glycation inhibitors extend yeast chronological lifespan by reducing advanced glycation end products and by back regulation of proteins involved in mitochondrial respiration. J Proteomics.

[33] Matyash V, Liebisch G, Kurzchalia TV, Shevchenko A, Schwudke D (2008). Lipid extraction by methyl-tert-butyl ether for high-throughput lipidomics. J Lipid Res.

[34] Coman C, Solari FA, Hentschel A, Sickmann A, Zahedi RP, Ahrends R (2016). Simultaneous metabolite, protein, lipid extraction (SIMPLEX): a combinatorial multimolecular omics approach for systems biology. Mol Cell Proteomics.

[35] Yao CH, Liu GY, Yang K, Gross RW, Patti GJ. Inaccurate quantitation of palmitate in metabolomics and isotope tracer studies due to plastics. Metabolomics. 2016;12. doi:10.1007/s11306-016-1081-y. 10.1007/s11306-016-1081-y PMC5049887 27721678

[36] Garikapati V, Karnati S, Bhandari DR, Baumgart-Vogt E, Spengler B (2019). High-resolution atmospheric-pressure MALDI mass spectrometry imaging workflow for lipidomic analysis of late fetal mouse lungs. Sci Rep.

[37] Karnati S, Garikapati V, Liebisch G, Van Veldhoven PP, Spengler B, Schmitz G (2018). Quantitative lipidomic analysis of mouse lung during postnatal development by electrospray ionization tandem mass spectrometry. PLoS ONE.

[38] Xu LN, Wang XY, Jiao YP, Liu XH (2018). Assessment of potential false positives via orbitrap-based untargeted lipidomics from rat tissues. Talanta.

[39] Narvaez-Rivas M, Zhang QB (2016). Comprehensive untargeted lipidomic analysis using core-shell C30 particle column and high field orbitrap mass spectrometer. J Chromatogr A.

[40] Merrill AH (2011). Sphingolipid and glycosphingolipid metabolic pathways in the era of sphingolipidomics. Chem Rev.

[41] Pettus BJ, Baes M, Busman M, Hannun YA, Van Veldhoven PP. Mass spectrometric analysis of ceramide perturbations in brain and fibroblasts of mice and human patients with peroxisomal disorders. Rapid Commun Mass Spectrom. 2004;18(14):1569–74. doi:10.1002/rcm.1520. 10.1002/rcm.1520 15282781

[42] Miyazaki C, Saitoh M, Itoh M, Yamashita S, Miyagishi M, Takashima S (2013). Altered phospholipid molecular species and glycolipid composition in brain, liver and fibroblasts of Zellweger syndrome. Neurosci Lett.

[43] Hama K, Nagai T, Nishizawa C, Ikeda K, Morita M, Satoh N (2013). Molecular species of phospholipids with very long chain fatty acids in skin fibroblasts of Zellweger syndrome. Lipids.

[44] Herzog K, Pras-Raves ML, Vervaart MA, Luyf AC, van Kampen AH, Wanders RJ (2016). Lipidomic analysis of fibroblasts from Zellweger spectrum disorder patients identifies disease-specific phospholipid ratios. J Lipid Res.

[45] Saitoh M, Sakakihara Y, Mizuguchi M, Itoh M, Takashima S, Iwamori M (2007). Increase of ceramide monohexoside and dipalmitoyl glycerophospholipids in the brain of Zellweger syndrome. Neurosci Lett.

[46] Nagura M, Saito M, Iwamori M, Sakakihara Y, Igarashi T (2004). Alterations of fatty acid metabolism and membrane fluidity in peroxisome-defective mutant ZP102 cells. Lipids.

[47] Wangler MF, Hubert L, Donti TR, Ventura MJ, Miller MJ, Braverman N (2018). A metabolomic map of Zellweger spectrum disorders reveals novel disease biomarkers. Genet Med.

[48] Dudek J (2017). Role of cardiolipin in mitochondrial signaling pathways. Front Cell Dev Biol.

[49] Baumgart E, Vanhorebeek I, Grabenbauer M, Borgers M, Declercq PE, Fahimi HD (2001). Mitochondrial alterations caused by defective peroxisomal biogenesis in a mouse model for Zellweger syndrome (PEX5 knockout mouse). Am J Pathol.

[50] Claypool SM, Koehler CM (2012). The complexity of cardiolipin in health and disease. Trends Biochem Sci.

[51] Herzog K, Pras-Raves ML, Ferdinandusse S, Vervaart MAT, Luyf ACM, van Kampen AHC (2018). Functional characterisation of peroxisomal beta-oxidation disorders in fibroblasts using lipidomics. J Inherit Metab Dis.

[52] Ferdinandusse S, Denis S, Mooijer PA, Zhang Z, Reddy JK, Spector AA (2001). Identification of the peroxisomal beta-oxidation enzymes involved in the biosynthesis of docosahexaenoic acid. J Lipid Res.

[53] Janssen A, Baes M, Gressens P, Mannaerts GP, Declercq P, Van Veldhoven PP. Docosahexaenoic acid deficit is not a major pathogenic factor in peroxisome-deficient mice. Lab Invest. 2000;80(1):31–5. doi:10.1038/labinvest.3780005. 10.1038/labinvest.3780005 10653000

[54] Martinez M (1992). Abnormal profiles of polyunsaturated fatty acids in the brain, liver, kidney and retina of patients with peroxisomal disorders. Brain Res.

[55] Itoyama A, Honsho M, Abe Y, Moser A, Yoshida Y, Fujiki Y (2012). Docosahexaenoic acid mediates peroxisomal elongation, a prerequisite for peroxisome division. J Cell Sci.

[56] Abe Y, Honsho M, Nakanishi H, Taguchi R, Fujiki Y (2014). Very-long-chain polyunsaturated fatty acids accumulate in phosphatidylcholine of fibroblasts from patients with Zellweger syndrome and acyl-CoA oxidase1 deficiency. Biochim Biophys Acta.

[57] Dorninger F, Brodde A, Braverman NE, Moser AB, Just WW, Forss-Petter S (2015). Homeostasis of phospholipids—the level of phosphatidylethanolamine tightly adapts to changes in ethanolamine plasmalogens. Biochim Biophys Acta Mol Cell Biol Lipids.

[58] Yagita Y, Shinohara K, Abe Y, Nakagawa K, Al-Owain M, Alkuraya FS (2017). Deficiency of a retinal dystrophy protein, acyl-CoA binding domain-containing 5 (ACBD5), impairs peroxisomal beta-oxidation of very-long-chain fatty acids. J Biol Chem.

[59] Herzog K, Pras-Raves ML, Ferdinandusse S, Vervaart MAT, Luyf ACM, van Kampen AHC (2018). Plasma lipidomics as a diagnostic tool for peroxisomal disorders. J Inherit Metab Dis.

[60] Chen C, Wang H, Chen B, Chen D, Lu C, Li H (2019). Pex11a deficiency causes dyslipidaemia and obesity in mice. J Cell Mol Med.

[61] Wangler MF, Chao YH, Bayat V, Giagtzoglou N, Shinde AB, Putluri N (2017). Peroxisomal biogenesis is genetically and biochemically linked to carbohydrate metabolism in Drosophila and mouse. PLoS Genet.

[62] Gasmi L, McLennan AG (2001). The mouse Nudt7 gene encodes a peroxisomal nudix hydrolase specific for coenzyme A and its derivatives. Biochem J.

[63] Song J, Baek IJ, Chun CH, Jin EJ (2018). Dysregulation of the NUDT7-PGAM1 axis is responsible for chondrocyte death during osteoarthritis pathogenesis. Nat Commun.

[64] Cho YM, Bae SH, Choi BK, Cho SY, Song CW, Yoo JK (2003). Differential expression of the liver proteome in senescence accelerated mice. Proteomics.

[65] Schollenberger L, Gronemeyer T, Huber CM, Lay D, Wiese S, Meyer HE (2010). RhoA regulates peroxisome association to microtubules and the actin cytoskeleton. PLoS ONE.

[66] Marelli M, Smith JJ, Jung S, Yi E, Nesvizhskii AI, Christmas RH (2004). Quantitative mass spectrometry reveals a role for the GTPase Rho1p in actin organization on the peroxisome membrane. J Cell Biol.

