# Supplementary material for: Sequential lipidomic, metabolomic, and proteomic analyses of serum, liver, and heart tissue specimens from peroxisomal biogenesis factor 11α knockout mice
Source: Anal Bioanal Chem. 2022 Jan 27;414(6):2235–50. doi: 10.1007/s00216-021-03860-0 (PMC8821073; doi:10.1007/s00216-021-03860-0)
Supplement: Supplementary file 1 — Supplementary file1 (PDF 2072 kb) [file 216_2021_3860_MOESM1_ESM.pdf]

Electronic Supplementary Material

Sequential lipidomic, metabolomic and proteomic analyses of serum, liver and heart tissue specimens from peroxisomal biogenesis factor 11α knockout mice

Vannuruswamy Garikapati[1,2], Claudia Colasante[2], Eveline Baumgart-Vogt[2*], Bernhard Spengler[1*]

[1]Institute of Inorganic and Analytical Chemistry, Justus Liebig University Giessen, 35392 Giessen, Germany

[2]Institute for Anatomy and Cell Biology II, Division of Medical Cell Biology, Justus Liebig University Giessen, 35392 Giessen, Germany

**Electronic Supplementary Material (ESM_1)**	**Page No**
Table S1	4
Table S2	5
Table S3	6
Table S4	7-9
Table S5	10
Table S6	11
Table S7	12
Table S8	13
Table S9	14
Table S10	15
Table S11	16
Figure S1	17
Figure S2	18
Figure S3	19
Figure S4	20-21
Figure S5	22-24
Figure S6	25
Table S12	26-28
Figure S7	29

Additional Electronic Supplementary Material

Electronic Supplementary Material (ESM_2; Data file S1)

Data file S1 Details on lipid identification and relative-quantification by LipidSearch software

Electronic Supplementary Material (ESM_3; Dataset S1)

Dataset S1 Lipidomics raw dataset (Microsoft Excel worksheets)

Electronic Supplementary Material (ESM_4; Dataset S2)

Dataset S2 Metabolomics raw dataset (Microsoft Excel worksheets)

Electronic Supplementary Material (ESM_5; Dataset S3)

Dataset S3 Proteomics raw dataset (Microsoft Excel worksheets)

Electronic Supplementary Material (ESM_6; Data file S2)

Data file S2 Proteomics raw data quality evaluation using RawMeat software

Electronic Supplementary Material (ESM_7; Data file S3)

Data file S3 Bioinformatics analysis of differentially abundant proteins using PANTHER and BiNGO

Table S1 Reversed-phase chromatographic separation method for lipidomics experiments independently in positive- and negative-ion mode

Time (min)	Flow rate (ml/min)	Mobile phase A (%)	Mobile phase B (%)
0	0.250	70	30
2	0.250	57	43
2.1	0.250	45	55
12	0.250	35	65
18	0.250	15	85
20	0.250	0	100
25	0.250	0	100
25.1	0.250	70	30
28	0.250	70	30

Mobile phase A: H_2O:ACN, 40:60 (v/v) with 10 mM ammonium formate and 0.1% formic acid

Mobile phase B: IPA:ACN, 90:10 (v/v) with 10 mM ammonium formate and 0.1% formic acid

H_2O: Water

ACN: Acetonitrile

IPA: Isopropyl alcohol/2-propanol

Table S2 Heated electrospray ionization (HESI-II) source parameters for lipidomics experiments separately in positive- and negative-ion mode

Tune parameters	Positive-ion mode	Negative-ion mode
Sheath gas (a.u.)	35	35
Auxiliary gas (a.u.)	12	12
Sweep gas (a.u.)	1	1
Spray voltage (kV)	4.35	4.00
Capillary temperature (^{0}C)	330	330
S-lens RF	55	55
Auxiliary heater temperature (^{0}C)	325	325

a.u.: Arbitrary units

kV: Kilovolt

RF: Radio frequency

Table S3 Full scan/data-dependent MS/MS acquisition (Full MS/ddMS^2, Top15) method parameters for lipidomics experiments individually in positive- and negative-ion mode

Method parameters	Positive-ion mode	Negative-ion mode
Time (min)	28	28
Internal lock mass	391.28421	-
Charge state (Z)	1	1
Exclusion list	On	-
Resolution	70,000 (35,000)	70,000 (35,000)
AGC target	1e^6 (1e^5)	1e^6 (1e^5)
Maximum IT (ms)	250 (75)	250 (75)
Scan range	200-1800	200-1800
TopN	15	15
Isolation window (*m/z*)	1	1
Stepped NCE	25, 30	20, 30, 40
Underfill ratio (%)	0.6	0.6
Intensity threshold	8e^3	8e^3
Exclude isotopes	On	On
Dynamic exclusion (s)	8	8

Values in the parenthesis represent for MS^2

AGC: Automatic gain control

IT: Ion injection time

m/z: mass-to-charge ratio

NCE: Normalized collision energy

Prior to the sequential omics data acquisition, Q Exactive Orbitrap MS was externally calibrated in both positive- and negative-ion mode. Further, in positive-ion mode *m/z* 391.28421 (diisooctyl phthalate) was used as internal lock mass while no lock masses were used in negative-ion mode. Exclusion *m/z* ion list (for lipidomics experiments in positive-ion mode) was prepared from the extraction blank injections, which were acquired on a cleaned LC-MS/MS system (after MS instrument bakeout, mass calibration and LC maintenance, etc.) using a brand-new reversed-phase LC column and fresh LC-MS grade solvents. In addition, solvent blank and corresponding pooled extract samples were acquired independently in positive- and negative-ion mode at scheduled intervals during all omics data acquisition

Table S4 Filters applied in LipidSearch software (v4.2.23) to remove/minimize false positives after the alignment process

Rej = false and ({MainGrade[c]}.in ("A", "B", "C") or {MainGrade[s1]}.in ("A", "B", "C") or {MainGrade[s2]}.in ("A", "B", "C")) and

((Class = "Cer"	and (MainIon = "+H" or MainIon = "+HCOO"))	or
(Class = "CerP"	and (MainIon = "+H" or MainIon = "+HCOO"))	or
(Class = "Hex1Cer"	and (MainIon = "+H" or MainIon = "+HCOO"))	or
(Class = "Hex2Cer"	and (MainIon = "+H" or MainIon = "+HCOO"))	or
(Class = "Hex3Cer"	and (MainIon = "+H" or MainIon = "+HCOO"))	or
(Class = "SPH"	and (MainIon = "+H" or MainIon = "+H-H_2O"))	or
(Class = "SPHP"	and (MainIon = "+H" or MainIon = "+H-H_2O"))	or
(Class = "ChE"	and (MainIon = "+NH_4" or MainIon = "+H-H_2O"))	or
(Class = "PIP"	and (MainIon = "+Na" or MainIon = "-H"))	or
(Class = "PIP2"	and (MainIon = "+Na" or MainIon = "-H"))	or
(Class = "SM"	and MainIon = "+H")	or
(Class = "MG"	and MainIon = "+H")	or
(Class = "AEA"	and MainIon = "+NH_4")	or
(Class = "CerG2GNAc1"	and (MainIon = "+H" or MainIon = "-H" or MainIon = "+H-H_2O"))	or
(Class = "OAHFA"	and MainIon = "-H")	or
(Class = "PEt"	and (MainIon = "+H" or MainIon = "-H"))	or
(Class = "PMe"	and (MainIon = "+H" or MainIon = "-H"))	or
(Class = "GM3"	and (MainIon = "+H" or MainIon = "-H"))	or
(Class = "GM2"	and (MainIon = "+H" or MainIon = "-H"))	or
(Class = "ST"	and (MainIon = "+H" or MainIon = "-H"))	or
(Class = "FA"	and MainIon = "-H"))	or

Rej = false and ({MainGrade[c]}.in ("A", "B") or {MainGrade[s1]}.in ("A", "B") or {MainGrade[s2]}.in ("A", "B")) and

((Class = "Co"	and (MainIon = "+NH_4" or MainIon = "+H"))	or
(Class = "DG"	and (MainIon = "+NH_4" or MainIon = "+Na"))	or

(Class = "TG"	and MainIon = "+NH_4")	or
(Class = "AcCa"	and MainIon = "+H")	or
(Class = "CL"	and MainIon = "-H")	or
(Class = "DLCL"	and MainIon = "-H")	or
(Class = "MLCL"	and MainIon = "-H")	or
(Class = "LPA"	and MainIon = "-H")	or
(Class = "PA"	and MainIon = "-H")	or
(Class = "LdMePE"	and (MainIon = "+H" or MainIon = "-H"))	or
(Class = "dMePE"	and (MainIon = "+H" or MainIon = "-H"))	or
(Class = "LPC"	and (MainIon = "+H" or MainIon = "+HCOO"))	or
(Class = "PC"	and (MainIon = "+H" or MainIon = "+HCOO"))	or
(Class = "LPE"	and (MainIon = "+H" or MainIon = "-H"))	or
(Class = "PE"	and (MainIon = "+H" or MainIon = "-H"))	or
(Class = "LPG"	and (MainIon = "+NH_4" or MainIon = "-H"))	or
(Class = "PG"	and (MainIon = "+NH_4" or MainIon = "-H"))	or
(Class = "LPI"	and (MainIon = "+NH_4" or MainIon = "-H"))	or
(Class = "PI"	and (MainIon = "+NH_4" or MainIon = "-H"))	or
(Class = "LPS"	and (MainIon = "+H" or MainIon = "-H"))	or
(Class = "PS"	and (MainIon = "+H" or MainIon = "-H")))	

LipidSearch software (v4.2.23) parameters

Search/Identification parameters

Database – General and HCD; Search Type – Product; Experiment Type – LC-MS; Parent Tolerance – 0.1 Da; NL/Prec Tol – 0.5 Da; Precursor Tolerance (MS) – 5.0 ppm; Product (MS/MS) Tolerance – 5.0 ppm; Merge Range (Min) – 0.0; Minimal Peak Width (min) – 0.0; Threshold Type – Relative; Product Ion – 1.0%; m-score threshold – 2.0; Recalculate Isotope – ON; R.T. Interval (min) – 0.0; Execute Quantitation – ON; *m/z* Tolerance – -5.0/+5.0; Tolerance Type – ppm; R.T. range (min) – -0.5/+0.5; Top rank filter – ON; Main Node Filter – Main Isomer Peak; m-Score Threshold (Display) – 5.0; C-Score Threshold (Display) – 2.0; Fatty Acid Priority (FA Priority) – ON; ID Quality Filter – (A, B, C, D); Adducts – (in positive-ion mode) +H, +NH_4, +Na, +K, +H-H_2O, +H-2H_2O, +2H; Adducts – (in negative-ion mode) -H, +HCOO, -CH_3, -2H

Alignment parameters

Search Type – Product; Experiment Type – LC-MS; Alignment Method – Mean; R.T. Tolerance – 0.25; Calculate Unassigned Peak Area – ON; Filter Type – New Filter; Top rank Filter – ON; Main Node Filter – All isomer peaks; m-score threshold – 5.0; ID Quality – A, B and/or C

Fragmentation grade/Identification level

Grade A: Lipid class and fatty acyl chains (FAs) were completely identified

Grade B: Lipid class and some FAs were identified

Grade C: Lipid class or FAs were identified

Grade D: Lipids identified based on neutral losses (e.g. H_2O)

Abbreviations

AEA: N-Acylethanolamine; AcCa: Acylcarnitines or Carnitine esters; CL: Cardiolipin; Cer: Ceramide; CerG2GNAc1, Hex1Cer, Hex2Cer and Hex3Cer: Simple Glc series; CerP: Ceramides phosphate; ChE: Cholesteryl esters and/or Cholesterol; Co: Coenzyme; DG: Diglyceride; DLCL: Cardiolipin (dilyso); FA: Fatty acid and/or Fatty acyl chain; GM2 and GM3: Gangliosides; LPA: Lysophosphatidic acid; LPC: Lysophosphatidylcholine; LPE: Lysophosphatidylethanolamine; LPG: Lysophosphatidylglycerol; LPI: Lysophosphatidylinositol; LPS: Lysophosphatidylserine; LdMePE: Lysodimethylphosphatidylethanolamine; MG: Monoglyceride; MLCL: Cardiolipin (monolyso); OAHFA: OAcyl-(gamma-hydroxy) fatty acid; PA: Phosphatidic acid; PC: Phosphatidylcholine; PE: Phosphatidylethanolamine; PEt: Phosphatidylethanol; PG: Phosphatidylglycerol; PI: Phosphatidylinositol; PIP: Phosphatidylinositol phosphate; PIP2: Phosphatidylinositol bisphosphate; PMe: Phosphatidylmethanol; PS: Phosphatidylserine; SM: Sphingomyelin; SPH: Sphingosine; SPHP: Sphingosine phosphate; ST: Sulfatide; TG: Triglyceride; dMePE: Dimethylphosphatidylethanolamine; C: Wild type control; S1: *Pex11α* knockout; S2: Pooled sample; Ion-adducts: +H, +Na, $+NH_4$, $+H-H_2O$, +HCOO and –H; m-score: Fragmentation match score; HCD: Higher-energy collisional dissociation; RT: Retention time

References

1. Peake DA et al., Processing of a complex lipid dataset for the NIST inter-laboratory comparison exercise for lipidomics measurements in human serum and plasma. ASMS 2015 Poster
2. Kiyonami R, Peake DA et al., Large scale lipid profiling of a human serum lipidome using a high-resolution accurate-mass LC/MS/MS approach. LIPID MAPS Annual Meeting 2015 Poster
3. Breitkopf SB, Ricoult SJH, Yuan M, Xu Y, Peake DA, Manning BD et al., A relative quantitative positive/negative ion switching method for untargeted lipidomics via high-resolution LC-MS/MS from any biological source. Metabolomics. 2017;13(3)
4. Li Z, Lai ZW, Christiano R, Gazos-Lopes F, Walther TC, Farese RV, Jr. Global analyses of selective insulin resistance in hepatocytes caused by palmitate lipotoxicity. Mol Cell Proteomics. 2018;17(5):836-49

Table S5 Reversed-phase chromatographic separation method for metabolomics experiments of tissue homogenates (liver and heart) independently in positive- and negative-ion mode

Time (min)	Flow rate (ml/min)	Mobile phase A (%)	Mobile phase B (%)
0	0.300	99	1
1.5	0.300	99	1
15	0.300	1	99
17.5	0.300	1	99
17.8	0.300	99	1
20	0.300	99	1

Mobile phase A: 100% water with 0.1% formic acid

Mobile phase B: 100% acetonitrile with 0.1% formic acid

Table S6 Reversed-phase chromatographic separation method for metabolomics experiments of serum samples independently in positive- and negative-ion mode

Time (min)	Flow rate (ml/min)	Mobile phase A (%)	Mobile phase B (%)
0	0.300	99	1
1	0.300	85	15
4	0.300	65	35
7	0.300	5	95
9	0.300	5	95
10	0.300	99	1
14	0.300	99	1

Mobile phase A: 100% water with 0.1% formic acid

Mobile phase B: 100% methanol with 0.1% formic acid

Table S7 Heated electrospray ionization (HESI-II) source parameters for metabolomics experiments separately in positive- and negative-ion mode

Tune parameters	Positive-ion mode	Negative-ion mode
Sheath gas (a.u.)	42	42
Auxiliary gas (a.u.)	11	11
Sweep gas (a.u.)	0	0
Spray voltage (kV)	4.0	3.5
Capillary temperature (^{0}C)	300	300
S-lens RF	55	55
Auxiliary heater temperature (^{0}C)	310	310

a.u.: Arbitrary units

kV: Kilovolt

RF: Radio frequency

Table S8 Full scan/data-dependent MS/MS acquisition (Full MS/ddMS^2, Top10) method parameters for metabolomics experiments individually in positive- and negative-ion mode

**Method parameters**	**Positive-ion mode**	**Negative-ion mode**
Time (min)	20 / 14	20 / 14
Internal lock mass	391.28421	-
Charge state (Z)	1	1
Resolution	70,000 (17,500)	70,000 (17,500)
AGC target	$1e^6$ ($1e^5$)	$1e^6$ ($1e^5$)
Maximum IT (ms)	120 (80)	120 (80)
Scan range	67-1000	67-1000
TopN	10	10
Isolation window (*m/z*)	1.5	1.5
Stepped NCE	30, 35	25, 30, 35
Underfill ratio (%)	0.7	0.7
Intensity threshold	$8e^3$	$8e^3$
Dynamic exclusion (s)	10	10

Values in the parenthesis represent for MS^2

AGC: Automatic gain control

IT: Ion injection time

m/z: mass-to-charge ratio

NCE: Normalized collision energy

Table S9 Reversed-phase chromatographic separation method for proteomics experiments in positive-ion mode

Time (min)	Flow rate (ml/min)	Mobile phase A (%)	Mobile phase B (%)
0	0.250	97	3
5	0.250	97	3
95	0.250	60	40
105	0.250	50	50
108	0.250	2	98
110	0.250	2	98
112	0.250	97	3
120	0.250	97	3

Mobile phase A: 100% water with 0.1% formic acid

Mobile phase B: 100% acetonitrile with 0.1% formic acid

Table S10 Heated electrospray ionization (HESI-II) source parameters for proteomics experiments in positive-ion mode

**Tune parameters**	**Positive-ion mode**
Sheath gas (a.u.)	30
Auxiliary gas (a.u.)	8
Sweep gas (a.u.)	0
Spray voltage (kV)	4.2
Capillary temperature (^{0}C)	320
S-lens RF	55
Auxiliary heater temperature (^{0}C)	200

a.u.: Arbitrary units

kV: Kilovolt

RF: Radio frequency

Table S11 Full scan/data-dependent MS/MS acquisition (Full MS/ddMS^2, Top10) method parameters for proteomics experiments in positive-ion mode

**Method parameters**	**Positive-ion mode**
Time (min)	120
Internal lock mass	391.28421
Charge state (Z)	2
Resolution	70,000 (17,500)
AGC target	$1e^6$ ($1e^5$)
Maximum IT (ms)	120 (120)
Scan range	350-1800
TopN	10
Isolation window (*m/z*)	3
NCE	30
Underfill ratio (%)	0.3
Intensity threshold	$2.5e^3$
Charge exclusion	Unassigned, 1
Peptide match	On
Dynamic exclusion (s)	15

Values in the parenthesis represent for MS^2

AGC: Automatic gain control

IT: Ion injection time

m/z: mass-to-charge ratio

NCE: Normalized collision energy

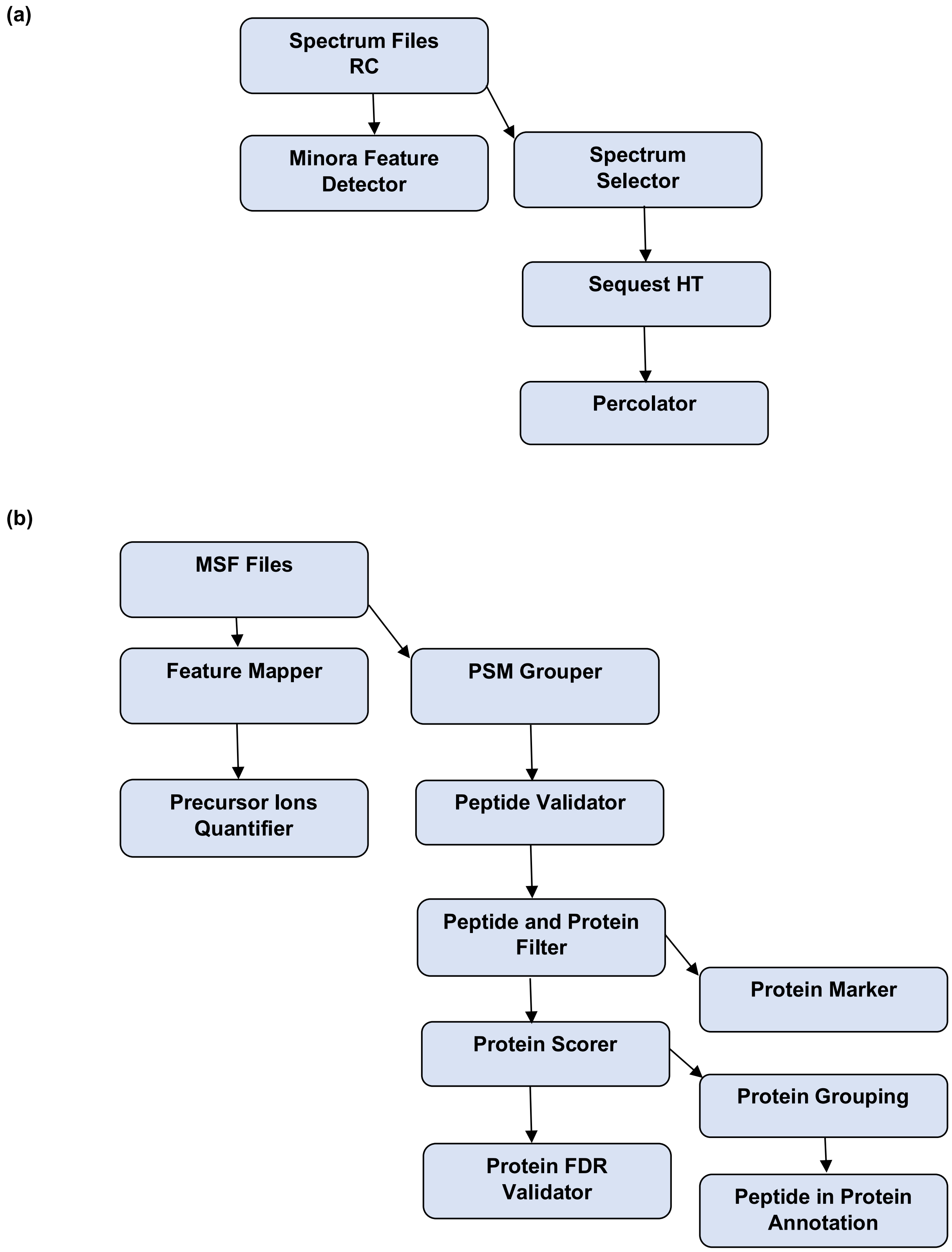

Fig. S1 Untargeted label-free relative-quantification workflow of Proteome Discoverer (v2.2.0.388) software (a) processing workflow and (b) consensus workflow

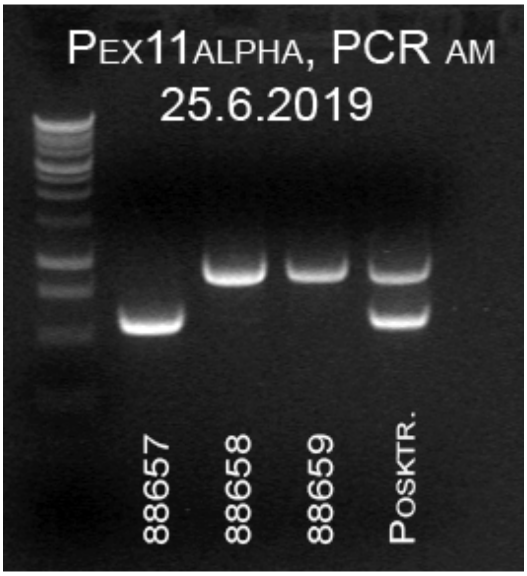

Fig. S2 Genotypes of animals were determined by polymerase chain reaction as described previously[1]. Wild type (e.g. 88657) allele (*Pex11α*$^{+/+}$) is amplified by primer 10 (5′-AATCAGGGACCTGTGCAACCTG-3′) and primer 11 (5′-AGTACAGCGTGGCTAATGAAGAGAC-3′). Homozygous peroxisomal biogenesis factor 11α knockout (e.g. 88658 and 88659) allele (*Pex11α*$^{-/-}$) is amplified by primer 10 (5′-AATCAGGGACCTGTGCAACCTG-3′) and Neo primer (5′-ATATTGCTGAAGAGCTTGGCGGC-3′). Both bands (e.g. POSKTR) amplified for heterozygous animals (*Pex11α*$^{+/-}$)

Reference

1. Li X, Baumgart E, Dong GX, Morrell JC, Jimenez-Sanchez G, Valle D, et al., PEX11alpha is required for peroxisome proliferation in response to 4-phenylbutyrate but is dispensable for peroxisome proliferator-activated receptor alpha-mediated peroxisome proliferation. Mol Cell Biol. 2002;22(23):8226-40

(a) Lipidomics data

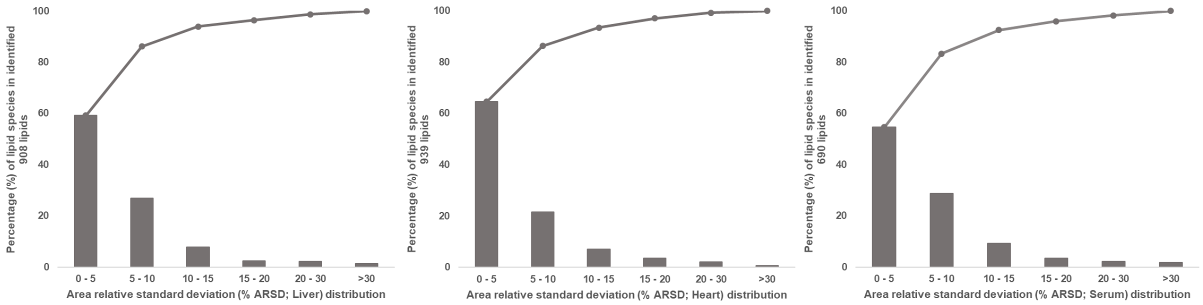

(b) Metabolomics data

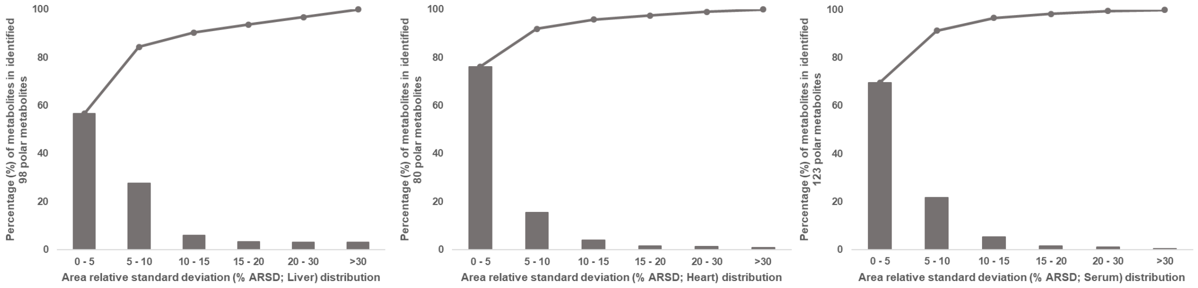

(c) Proteomics data

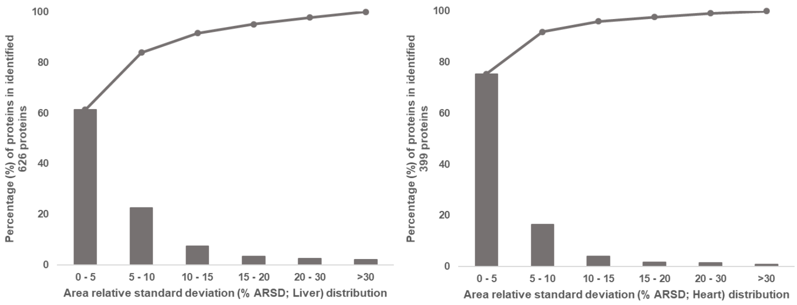

Fig. S3 Histograms and integral of area relative standard deviation (% ARSD) values for identified (a) lipids, (b) polar metabolites and (c) proteins in liver (left column), heart (middle column) and serum (right column, except for proteomics data) across all replicates

(a)

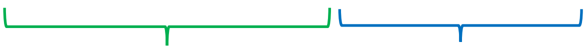

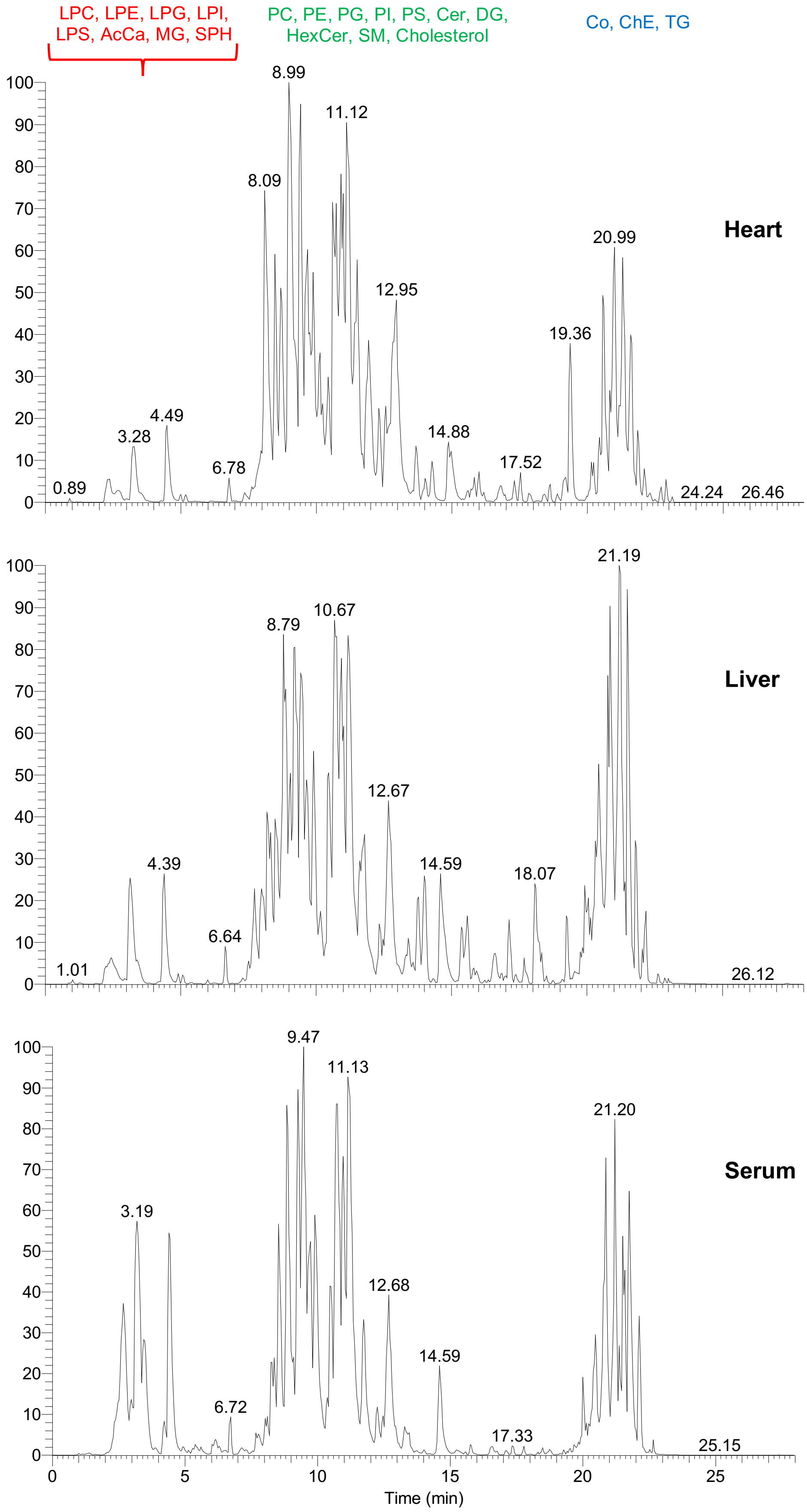

Fig. S4 a) Base peak chromatograms of the mouse heart, liver and serum lipidome extracted by MeOH/MTBE/H_2O liquid-liquid extraction method in positive-ion mode

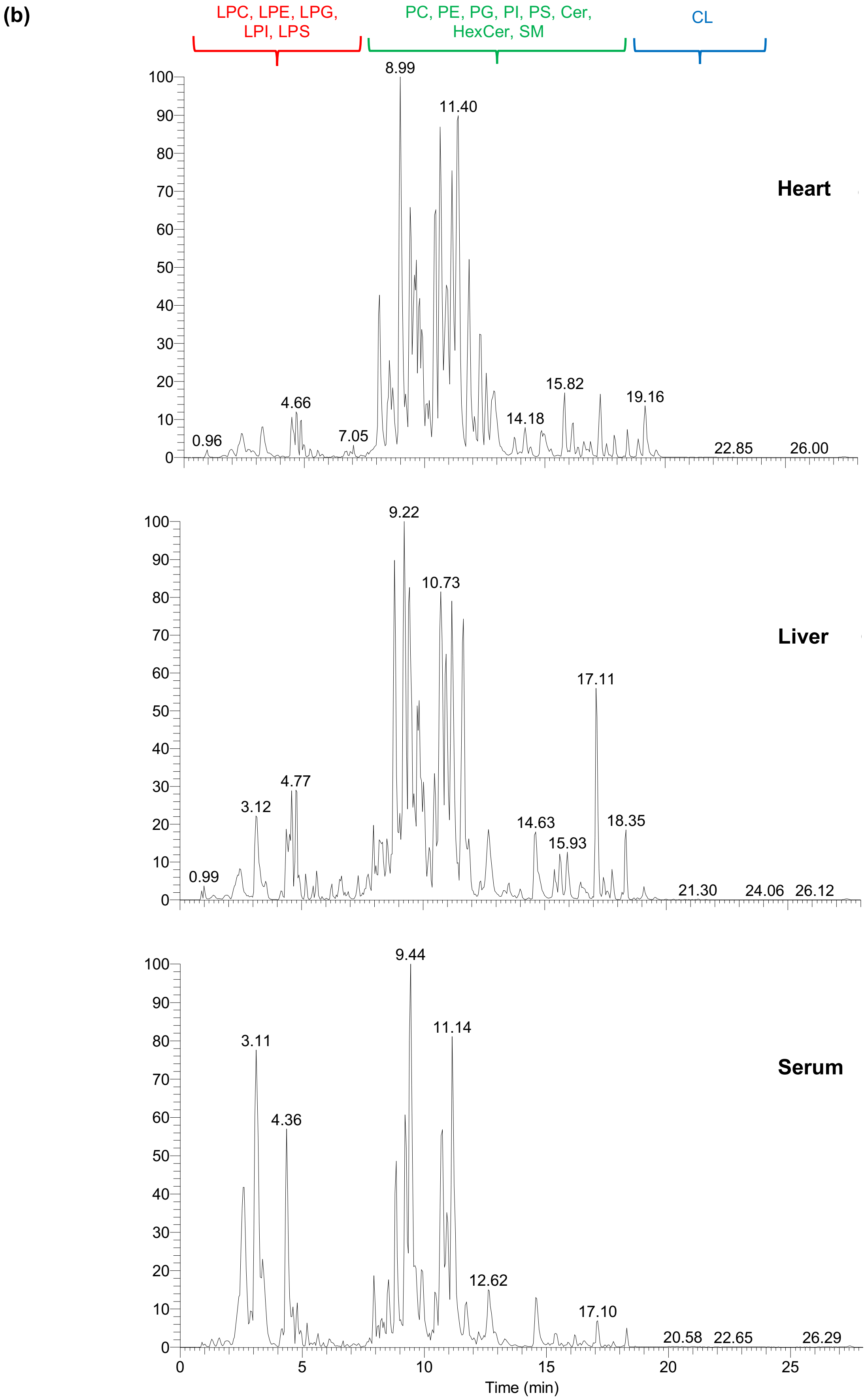

Fig. S4 b) Base peak chromatograms of the mouse heart, liver and serum lipidome extracted by MeOH/MTBE/H_2O liquid-liquid extraction method in negative-ion mode

(a)

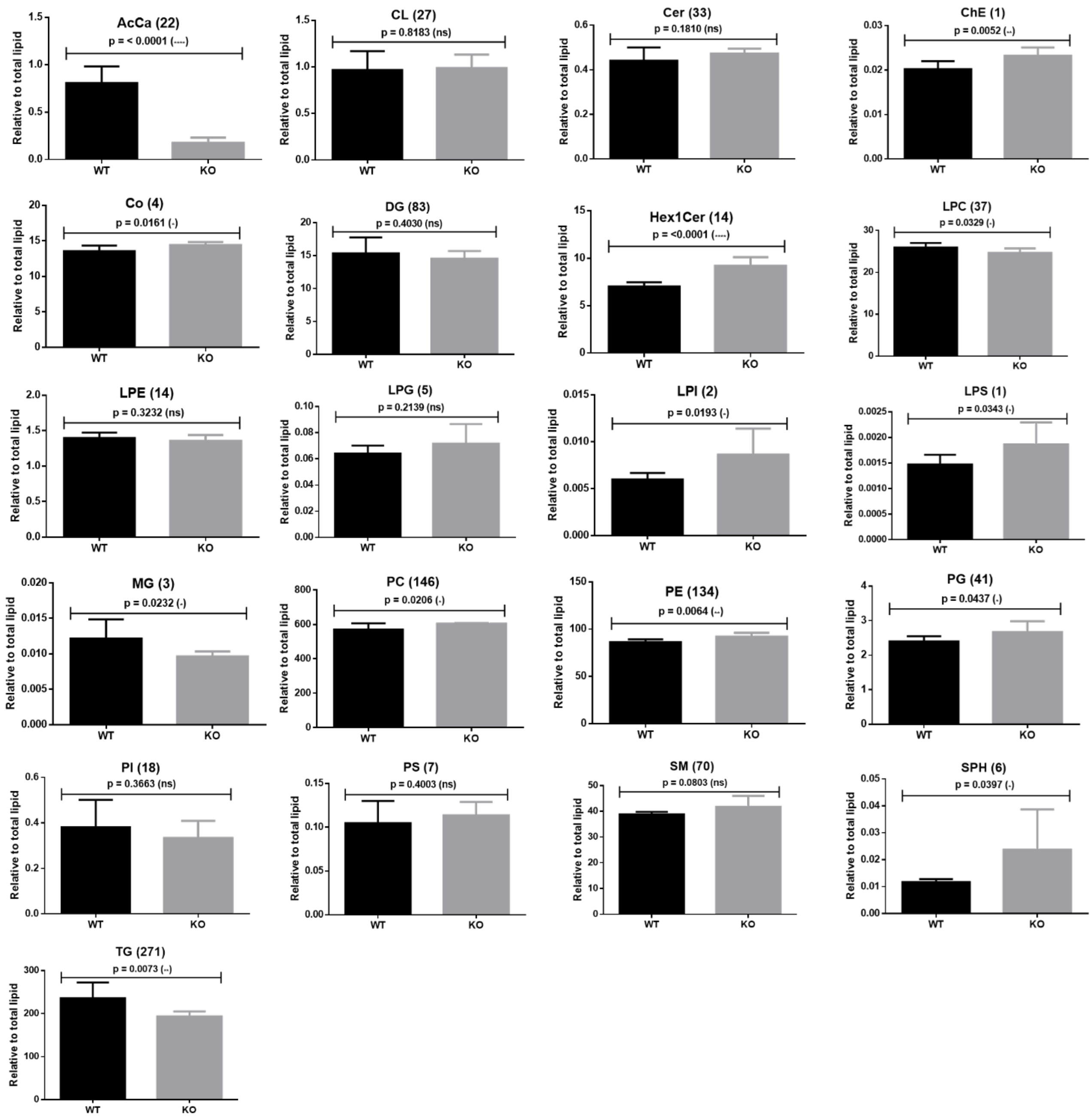

Fig. S5 a) Comparison of individual lipid classes (in total 21) between WT control and *Pex11α* KO mouse heart tissue homogenates. Total lipid class value represents the sum of the normalized relative abundance values of all measured lipid species in a particular lipid class (combined positive- and negative-ion mode data from three biological replicates and technical triplicates). The numbers in parenthesis denote the number of confident lipid species identifications within each lipid class. Underlying numerical data (mean values ± standard deviation) used to generate the bar graphs is presented in the additional electronic supplementary material dataset S1 (ESM_3), under the Microsoft Excel worksheet name_Lipid class

(b)

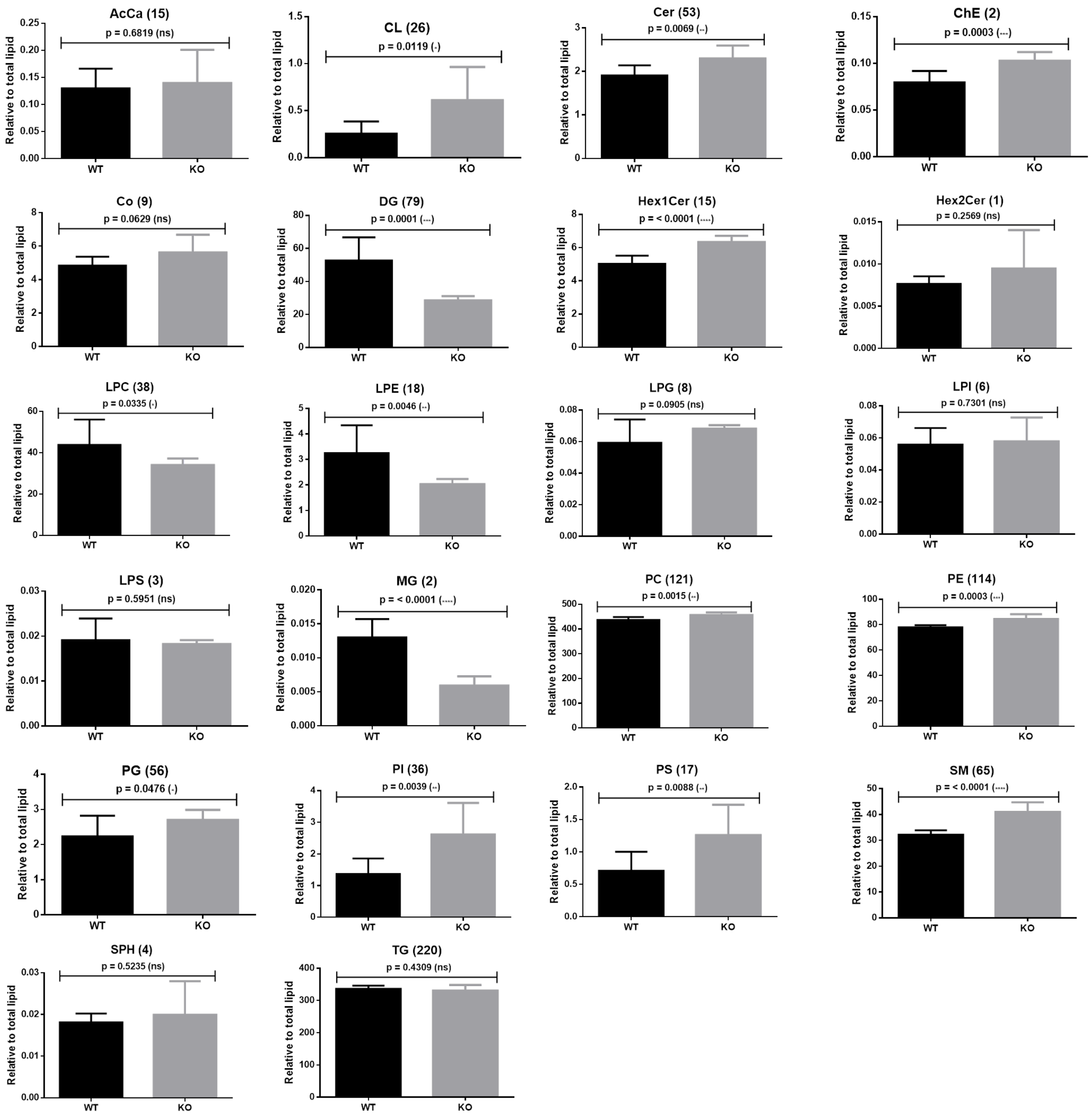

Fig. S5 b) Comparison of individual lipid classes (in total 22) between WT control and *Pex11α* KO mouse liver tissue homogenates. Total lipid class value represents the sum of the normalized relative abundance values of all measured lipid species in a particular lipid class (combined positive- and negative-ion mode data from three biological replicates and technical triplicates). The numbers in parenthesis denote the number of confident lipid species identifications within each lipid class. Underlying numerical data (mean values ± standard deviation) used to generate the bar graphs is presented in the additional electronic supplementary material dataset S1 (ESM_3), under the Microsoft Excel worksheet name_Lipid class

(c)

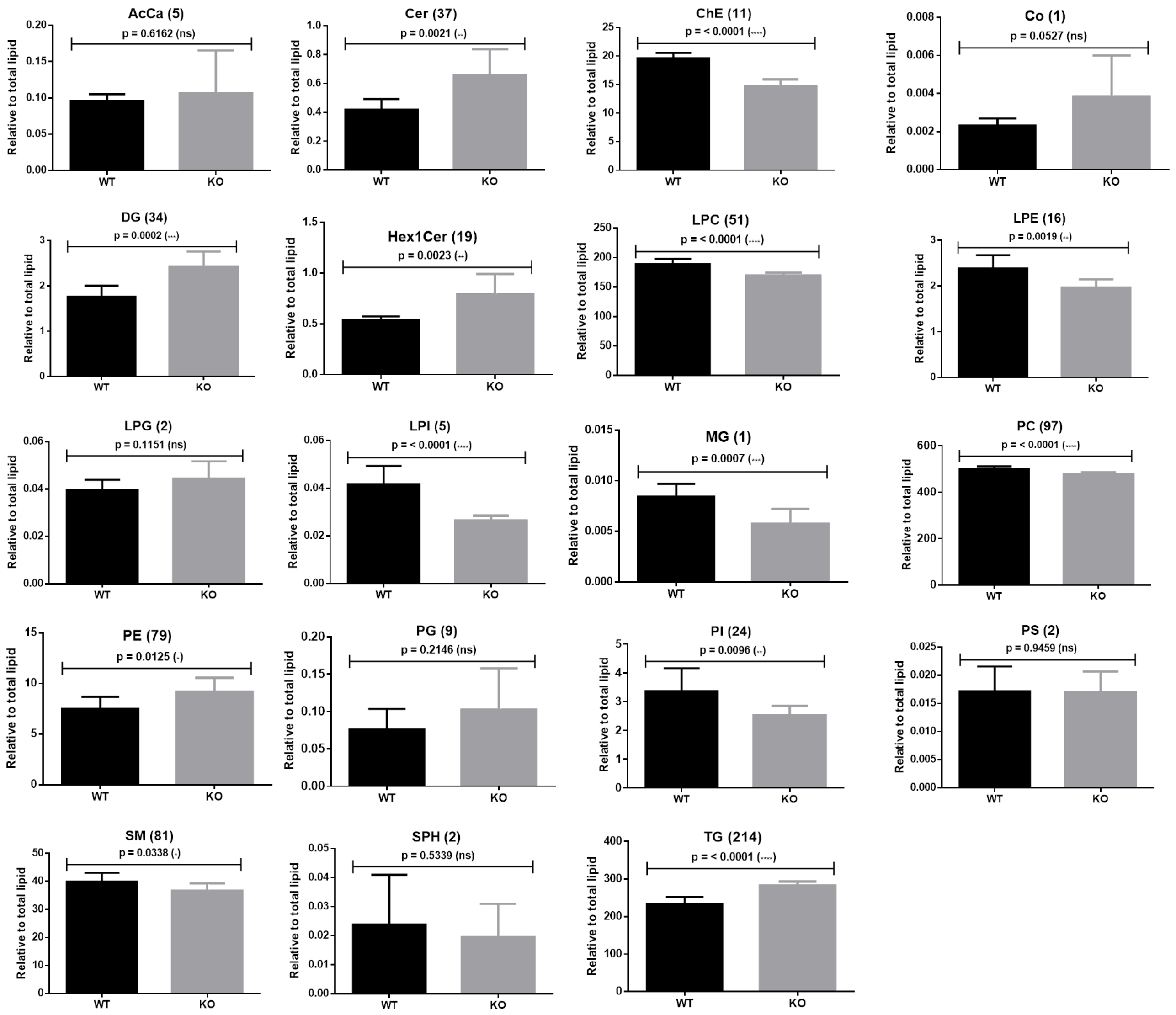

Fig. S5 c) Comparison of individual lipid classes (in total 19) between WT control and *Pex11α* KO mouse serum samples. Total lipid class value represents the sum of the normalized relative abundance values of all measured lipid species in a particular lipid class (combined positive- and negative-ion mode data from three biological replicates and technical triplicates). The numbers in parenthesis denote the number of confident lipid species identifications within each lipid class. Underlying numerical data (mean values ± standard deviation) used to generate the bar graphs is presented in the additional electronic supplementary material dataset S1 (ESM_3), under the Microsoft Excel worksheet name_Lipid class

Serum lipidome

a) b) c)

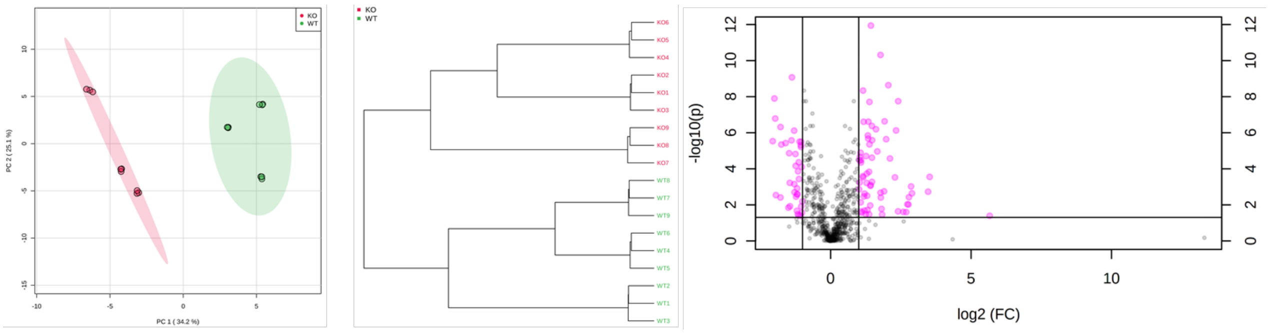

Heart lipidome

a) b) c)

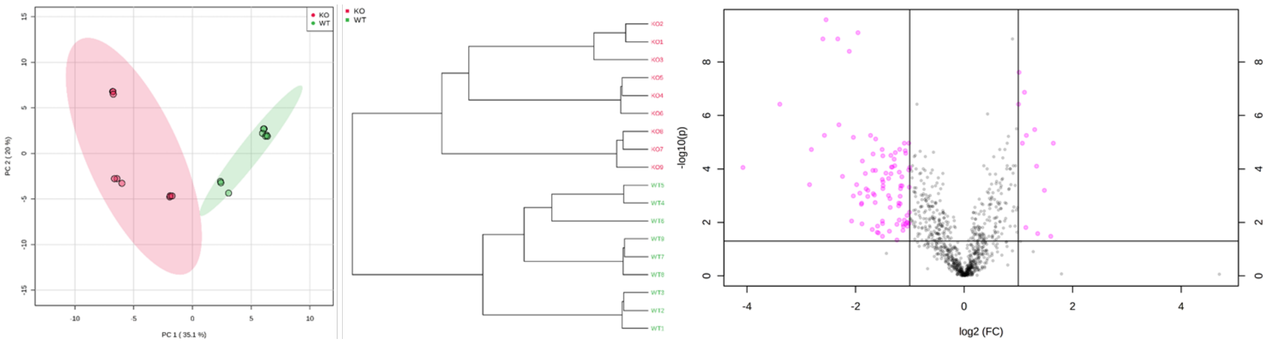

Liver lipidome

a) b) c)

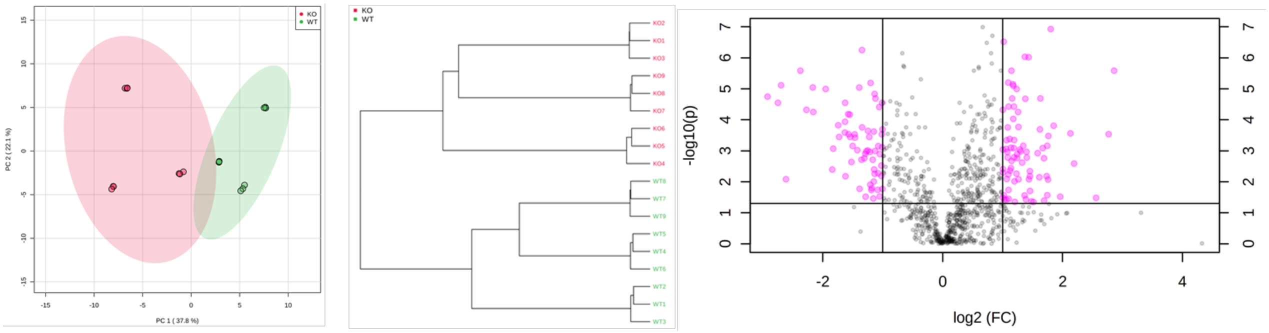

Fig. S6 a) Principal component analysis (PCA), b) hierarchical clustering analysis (HCA; dendrogram), and c) volcano plots (log2 fold changes versus -log10 FDR adjusted p-values) analyses of lipidomics data (combined positive- and negative-ion mode data from three biological replicates and technical triplicates) obtained from WT control and *Pex11α* KO mouse serum, heart and liver tissue homogenates

Table S12 a) Metabolic pathway analysis of combined differentially abundant lipids and polar metabolites (serum) using MetPA module of MetaboAnalyst (v4.0) web application

Pathway	Total	Expected	Hits	Raw p	-log10 p	Holm adjust	FDR	Impact
Linoleic acid metabolism	5	0.086321	2	0.002777	2.5564	0.2333	0.2333	0
Glycerophospholipid metabolism	36	0.62151	3	0.022363	1.6505	1	0.88296	0.21631
Tryptophan metabolism	41	0.70784	3	0.031534	1.5012	1	0.88296	0.23722
Cysteine and methionine metabolism	33	0.56972	2	0.10918	0.96185	1	1	0.02659
Arachidonic acid metabolism	36	0.62151	2	0.12628	0.89866	1	1	0
Tyrosine metabolism	42	0.7251	2	0.16222	0.7899	1	1	0.02559
alpha-Linolenic acid metabolism	13	0.22444	1	0.20333	0.69181	1	1	0
Glycosylphosphatidylinositol (GPI)-anchor biosynthesis	14	0.2417	1	0.2172	0.66314	1	1	0.00399
Glycerolipid metabolism	16	0.27623	1	0.24425	0.61216	1	1	0.01402
Sphingolipid metabolism	21	0.36255	1	0.30801	0.51143	1	1	0.03854
Purine metabolism	66	1.1394	2	0.31679	0.49923	1	1	0.02416
Alanine, aspartate and glutamate metabolism	28	0.4834	1	0.38865	0.41044	1	1	0.04808
Glycine, serine and threonine metabolism	34	0.58699	1	0.4505	0.3463	1	1	0.04655
Steroid biosynthesis	42	0.7251	1	0.52368	0.28093	1	1	0
Primary bile acid biosynthesis	46	0.79416	1	0.55666	0.25441	1	1	0
Aminoacyl-tRNA biosynthesis	48	0.82869	1	0.57231	0.24237	1	1	0

Table S12 b) Metabolic pathway analysis of combined differentially abundant lipids and polar metabolites (liver) using MetPA module of MetaboAnalyst (v4.0) web application

Pathway	Total	Expected	Hits	Raw p	-log10 p	Holm adjust	FDR	Impact
Glycerophospholipid metabolism	36	0.50199	4	0.001234	2.9089	0.10361	0.10361	0.26332
Sphingolipid metabolism	21	0.29283	3	0.002647	2.5772	0.21971	0.11118	0.30832
Lysine degradation	25	0.34861	2	0.045823	1.3389	1	1	0.14554
Linoleic acid metabolism	5	0.069721	1	0.067891	1.1682	1	1	0
alpha-Linolenic acid metabolism	13	0.18127	1	0.16747	0.77606	1	1	0
Glycosylphosphatidylinositol (GPI)-anchor biosynthesis	14	0.19522	1	0.17918	0.74671	1	1	0.00399
Pantothenate and CoA biosynthesis	19	0.26494	1	0.23542	0.62816	1	1	0.02857
beta-Alanine metabolism	21	0.29283	1	0.25687	0.59029	1	1	0.10448
Cysteine and methionine metabolism	33	0.46016	1	0.37401	0.42711	1	1	0
Glycine, serine and threonine metabolism	34	0.4741	1	0.38294	0.41687	1	1	0.04655
Arachidonic acid metabolism	36	0.50199	1	0.40043	0.39748	1	1	0
Arginine and proline metabolism	38	0.52988	1	0.41744	0.37941	1	1	0
Pyrimidine metabolism	39	0.54382	1	0.42577	0.37082	1	1	0.01318
Tryptophan metabolism	41	0.57171	1	0.4421	0.35448	1	1	0.03991
Primary bile acid biosynthesis	46	0.64143	1	0.48101	0.31785	1	1	0.02285

Table S12 c) Metabolic pathway analysis of combined differentially abundant lipids and polar metabolites (heart) using MetPA module of MetaboAnalyst (v4.0) web application

Pathway	Total	Expected	Hits	Raw p	-log10 p	Holm adjust	FDR	Impact
Glycerophospholipid metabolism	36	0.28685	3	0.0023819	2.6231	0.20008	0.91856	0.21631
Arginine and proline metabolism	38	0.30279	2	0.034898	1.4572	1	0.91856	0
Linoleic acid metabolism	5	0.039841	1	0.039262	1.406	1	0.91856	0
Vitamin B6 metabolism	9	0.071713	1	0.069649	1.1571	1	1	0
alpha-Linolenic acid metabolism	13	0.10359	1	0.099152	1.0037	1	1	0
Glycosylphosphatidylinositol (GPI)-anchor biosynthesis	14	0.11155	1	0.10639	0.97309	1	1	0.00399
Starch and sucrose metabolism	15	0.11952	1	0.11358	0.9447	1	1	0.08784
beta-Alanine metabolism	21	0.16733	1	0.1556	0.80799	1	1	0.05597
Glutathione metabolism	28	0.22311	1	0.20232	0.69397	1	1	0
Cysteine and methionine metabolism	33	0.26295	1	0.23422	0.63038	1	1	0.02089
Glycine, serine and threonine metabolism	34	0.27092	1	0.24046	0.61896	1	1	0.04655
Arachidonic acid metabolism	36	0.28685	1	0.25279	0.59723	1	1	0
Fatty acid degradation	39	0.31076	1	0.27096	0.5671	1	1	0

Serum metabolome

a)

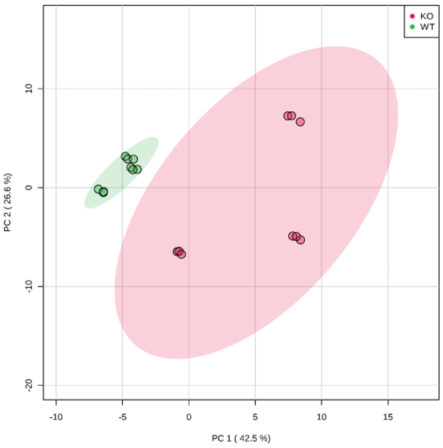

b)

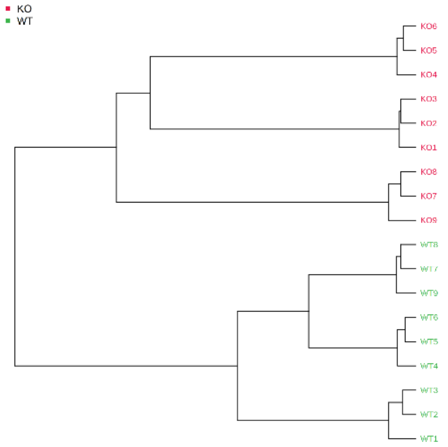

Liver metabolome

a)

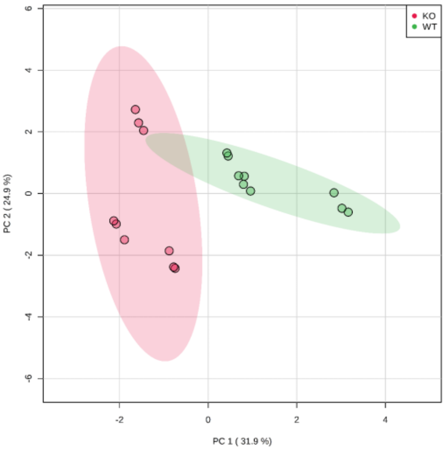

b)

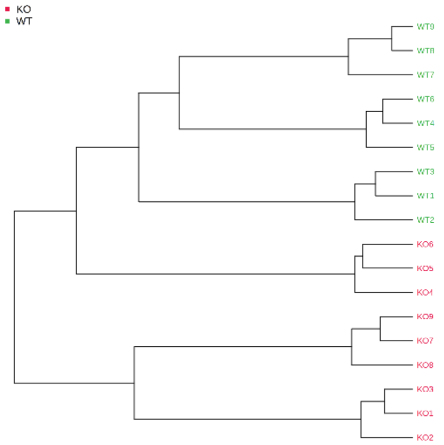

Heart metabolome

a)

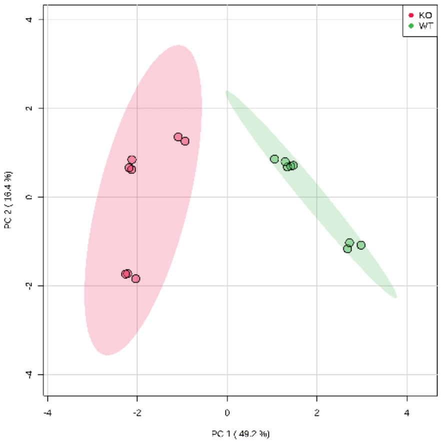

b)

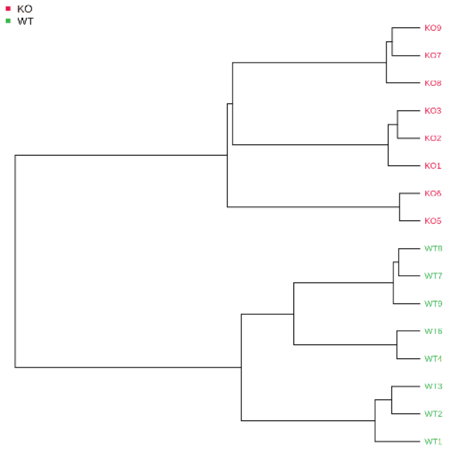

Fig. S7 a) Principal component analysis (PCA), b) hierarchical clustering analysis (HCA; dendrogram) of metabolomics data (combined positive- and negative-ion mode data from three biological replicates and technical triplicates) obtained from WT control and *Pex11α* KO mouse serum, liver and heart tissue homogenates
