# Supplementary material for: Sequential lipidomic, metabolomic, and proteomic analyses of serum, liver, and heart tissue specimens from peroxisomal biogenesis factor 11α knockout mice
Source: Anal Bioanal Chem. 2022 Jan 27;414(6):2235–50. doi: 10.1007/s00216-021-03860-0 (PMC8821073; doi:10.1007/s00216-021-03860-0)
Supplement: Supplementary file 2 — Supplementary file2 (PDF 7935 kb) [file 216_2021_3860_MOESM2_ESM.pdf]

Electronic Supplementary Material_2; Data file S1

Sequential lipidomic, metabolomic and proteomic analyses of serum, liver and heart tissue specimens from peroxisomal biogenesis factor 11α knockout mice

Vannuruswamy Garikapati[1,2], Claudia Colasante[2], Eveline Baumgart-Vogt[2*], Bernhard Spengler[1*]

[1]Institute of Inorganic and Analytical Chemistry, Justus Liebig University Giessen, 35392 Giessen, Germany

[2]Institute for Anatomy and Cell Biology II, Division of Medical Cell Biology, Justus Liebig University Giessen, 35392 Giessen, Germany

Details on lipid identification and relative-quantification by LipidSearch (v4.2.23) software[1,2]

Manual curation criteria

- Mass accuracy (MS ≤ 5 ppm and MS/MS ≤ 5 ppm)
- Confirmation of lipid identifications with multiple ion adducts and their diagnostic fragment ions (lipid class-specific fragments and lipid species-specific fragments) in both positive- and negative-ion mode[3-5]
- Fragmentation grade (identification level A, B and/or C)
- Base retention time window of lipid classes (as shown in ESM_1; Fig. S4)
- Isotopic profile and fatty acid distribution pattern
- Peak shape and quality
- Chromatographic peak integration and reassignment
- Fragmentation match score (m-score), c-score and t-score
- Area relative standard deviation (as shown in ESM_1; Fig. S3) and signal-to-noise ratio

References

1. Peake DA et al., Processing of a complex lipid dataset for the NIST inter-laboratory comparison exercise for lipidomics measurements in human serum and plasma. ASMS 2015 Poster
2. Kiyonami R, Peake DA et al., Large scale lipid profiling of a human serum lipidome using a high-resolution accurate-mass LC/MS/MS approach. LIPID MAPS Annual Meeting 2015 Poster
3. Murphy RC. Tandem mass spectrometry of lipids: molecular analysis of complex lipids. Royal Society of Chemistry, 2014
4. Lipidomics Standards Initiative guidelines (https://lipidomics-standards-initiative.org/guidelines)
5. Xu LN et al., Assessment of potential false positives via orbitrap-based untargeted lipidomics from rat tissues. Talanta 2018, 178, 287-293

Chromatogram

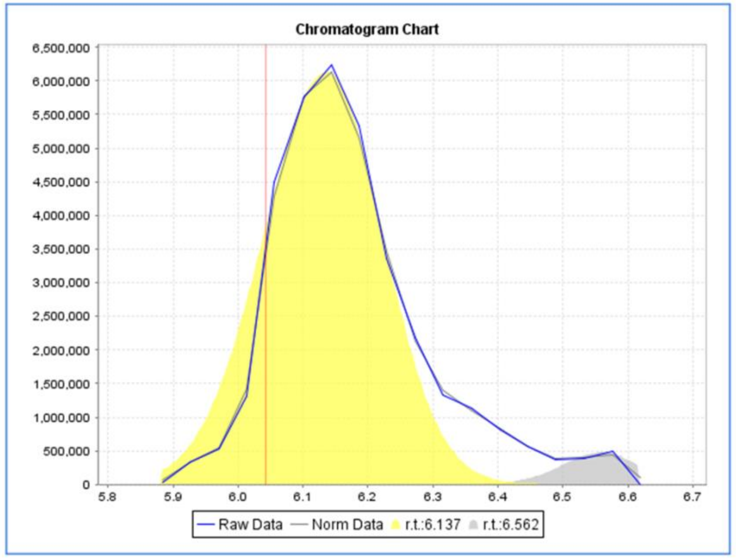

Spectrum

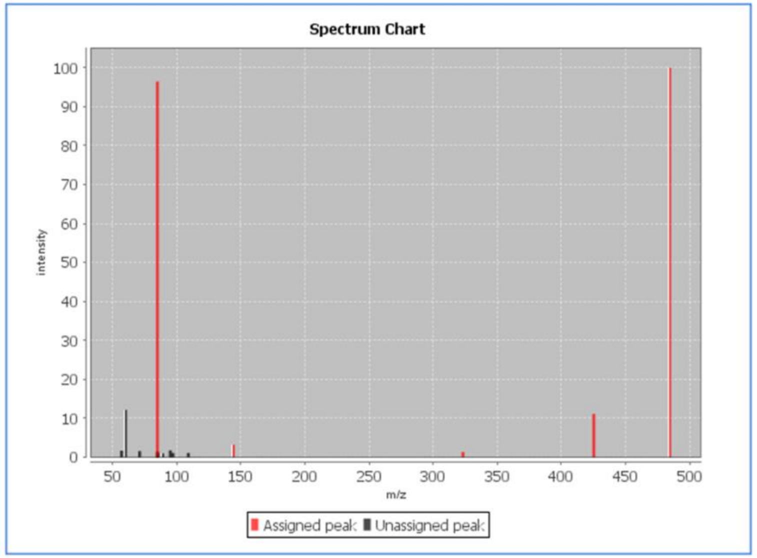

Match Lipid

LipidIon	M-Sc.	T-Sc.	Occ.	St.
◉ AcCa(22:0)+H	32.9	0.2	82.2	⊠

Match Detail

ObsMz	Type	It.(%)	Frag.	Delta(Da)
57.0708	MS2	1.699	-	-
60.0817	MS2	12.188	-	-
71.0864	MS2	1.596	-	-
85.0069	MS2	1.224	-	-
85.0291	MS2	96.426	NL[FA(22:0)-N(	0.0007
85.0497	MS2	1.516	-	-
85.1019	MS2	1.053	-	-
89.0604	MS2	1.004	-	-
95.086	MS2	1.772	-	-
97.1016	MS2	1.14	-	-
109.1017	MS2	1.128	-	-
144.1021	MS2	3.24	NL[FA(22:0)]	0.0002
323.3316	MS2	1.356	FA(22:0)-OH	0.0008
425.3631	MS2	11.138	M+H-N(CH3)3	0.0005
484.4365	MS2	100	M+H	0.0005

Copy

Acylcarnitines or Carnitine esters (AcCa)

<u>Information</u>

- Ionization: Positive-ion mode
- Main ion adduct used for identification: $[M+H]^+$
- Identification (fragmentation) grade: A and B
- Main ion adduct used for quantification: $[M+H]^+$

Representation of acylcarnitines or carnitine esters (AcCa) identification information obtained with LipidSearch software
e.g. $[AcCa(22:0)+H]^+$

Chromatogram

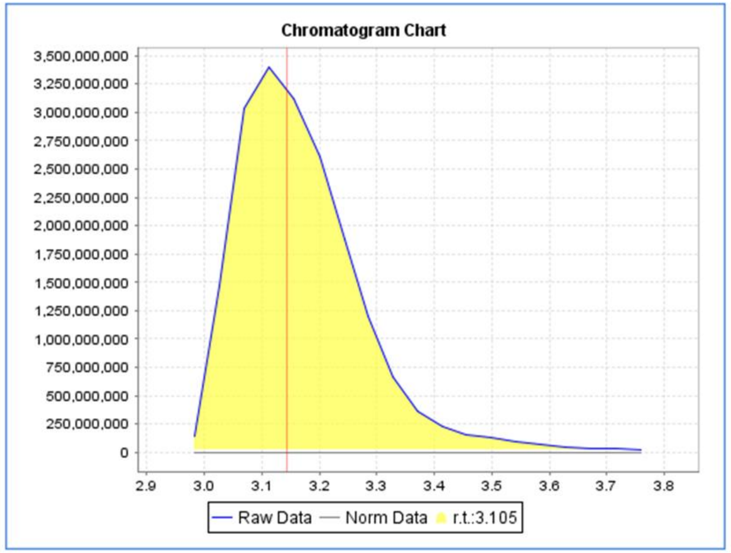

Spectrum

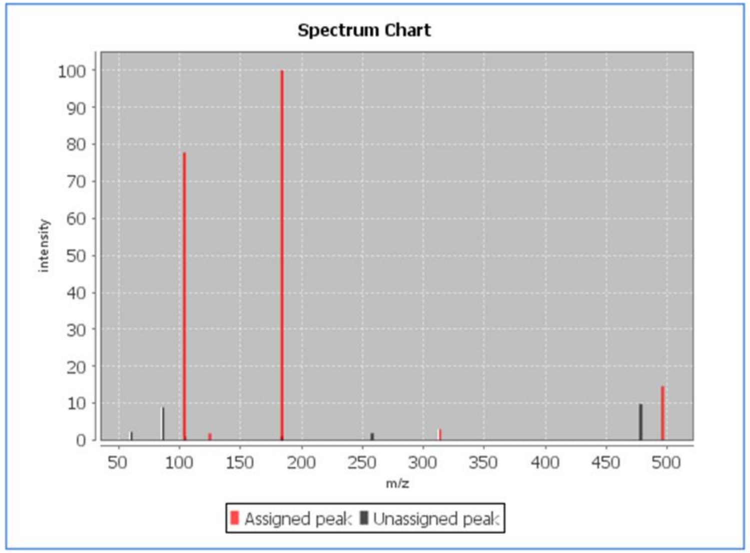

Match Lipid

LipidIon	M-Sc.	T-Sc.	Occ.	St.
LPC(16:0)+H	44	0.1	87.9	
PC(8:0e_8:0)+H	35.2	0.1	87.9	
PC(10:0e_6:0)+H	35.2	0.1	87.9	

Match Detail

ObsMz	Type	It.(%)	Frag.	Delta(Da)
60.0818	MS2	2.233	-	-
86.0973	MS2	8.918	-	-
104.1076	MS2	77.821	C5H14N1O1	0.0007
104.137	MS2	1.047	-	-
125.0002	MS2	1.896	C2H6O4P1	0.0004
184.0738	MS2	100	(P-Cho)+H	0.0005
184.1428	MS2	1.101	-	-
258.111	MS2	1.925	-	-
313.2747	MS2	2.973	M+H-PC	0.001
478.3304	MS2	9.807	-	-
496.3411	MS2	14.628	M+H	0.0013

Copy

Lysophosphatidylcholine (LPC)

Information

- Ionization**:** Positive-ion mode
- Main ion adducts used for identification**:** $[M+H]^+$, $[M+Na]^+$ and $[M+K]^+$
- Identification (fragmentation) grade**:** A and B
- Main ion adduct used for quantification**:** $[M+H]^+$

Representation of lysophosphatidylcholine (LPC) identification information obtained with LipidSearch software
e.g. $[LPC(16:0)+H]^+$

Lysophosphatidylcholine (LPC)

Chromatogram

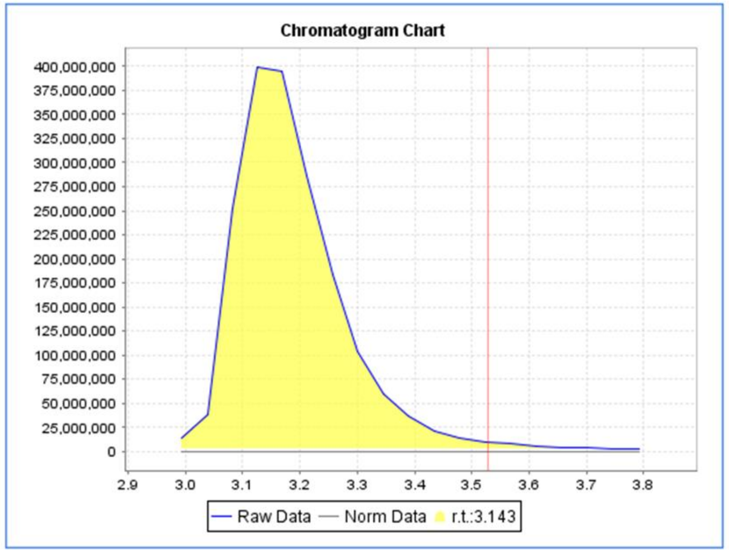

Spectrum

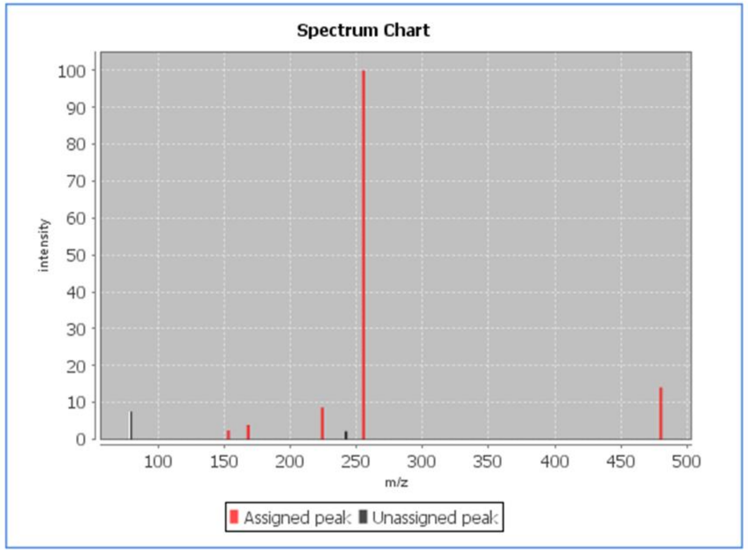

Match Lipid

LipidIon	M-Sc.	T-Sc.	Occ.	St.
◉ LPC(16:0)+HCOO	46.5	0	93	☒
○ PC(8:0e_8:0)+HCOO	5.8	0	19.2	☒
○ PC(10:0e_6:0)+HCOO	5.8	0	19.2	☒

Match Detail

ObsMz	Type	It.(%)	Frag.	Delta(Da)
78.9573	MS2	7.528	-	-
152.9947	MS2	2.436	GP-H3O	-0.0012
168.042	MS2	3.925	(P-Cho)-CH3-H	-0.0011
224.0689	MS2	8.675	M-FA(16:0)-CH3	-0.0005
242.0801	MS2	2.178	-	-
255.233	MS2	100	FA(16:0)-H	0.0001
480.3102	MS2	14.119	M-CH3	0.0007

Copy

Information

- **Ionization:** Negative-ion mode
- **Main ion adducts used for identification:** $[M+HCOO]^-$ and $[M\text{-}CH_3]^-$
- **Identification (fragmentation) grade:** A and B
- **Main ion adduct used for quantification:** $[M+HCOO]^-$

Representation of lysophosphatidylcholine (LPC) identification information obtained with LipidSearch software
e.g. $[LPC(16:0)+HCOO]^-$

Chromatogram

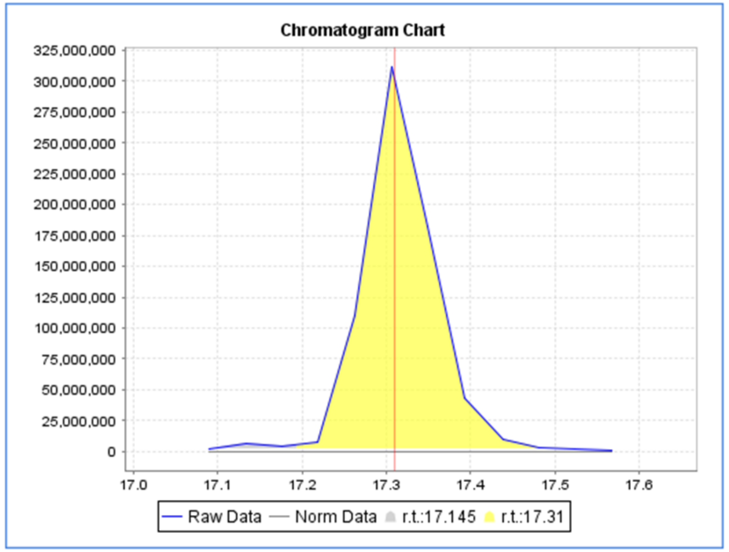

Spectrum

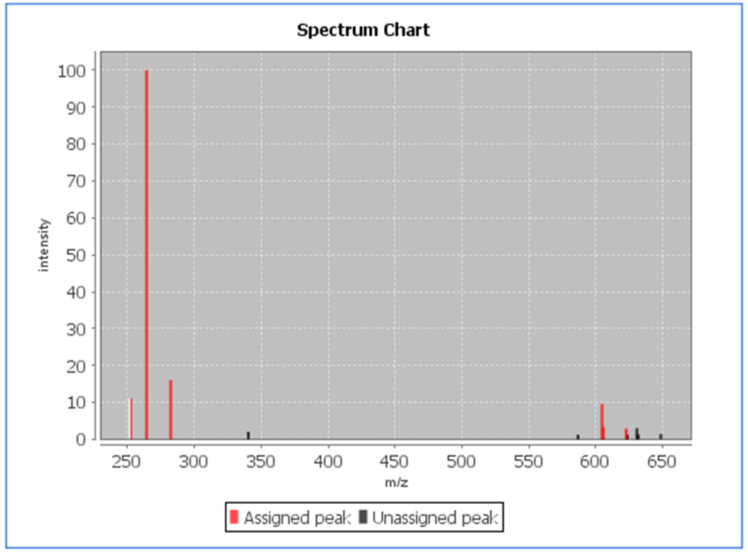

Match Lipid

LipidIon	M-Sc.	T-Sc.	Occ.	St.
**Cer(d18:1_22:0)+H**	**37.5**	**0.7**	**93.8**	☒
Cer(t18:0_22:0)+H-H2O	25.5	1.4	85.1	☒
Cer(d17:0_23:1)+H	3.2	0.7	16.1	☒
Cer(m17:1_23:0+O)+H	3.2	0.7	16.1	☒
Cer(d22:0_18:1)+H	2	0.7	10	☒
Cer(m22:1_18:0+O)+H	2	0.7	10	☒

Match Detail

ObsMz	Type	It.(%)	Frag.	Delta(Da)
252.2692	MS2	11.168	SPH(d18:1)+H-H2O-CH2O	0.0006
264.2691	MS2	100	SPH(d18:1)+H-2H2O	0.0006
282.2799	MS2	16.076	SPH(d18:1)+H-H2O	0.0007
340.3584	MS2	2.077	-	-
586.5937	MS2	1.283	-	-
604.6048	MS2	9.587	NL[H2O]	0.0021
605.6079	MS2	3.334	NL[H2O] [isotope]	1.0052
622.6149	MS2	2.921	M+H	0.0016
623.6176	MS2	1.264	-	-
630.6202	MS2	3.034	-	-
631.6215	MS2	1.418	-	-
648.6301	MS2	1.506	-	-

Copy

Ceramides (Cer)

Information

- **Ionization:** Positive-ion mode
- **Main ion adducts used for identification:** $[M+H]^+$, $[M+H-H_2O]^+$ and $[M+H-2H_2O]^+$
- **Identification (fragmentation) grade:** A , B and C
- **Main ion adduct used for quantification:** $[M+H]^+$

Representation of ceramides (Cer) identification information obtained with LipidSearch software e.g. $[Cer(d40:1)+H]^+$

Chromatogram

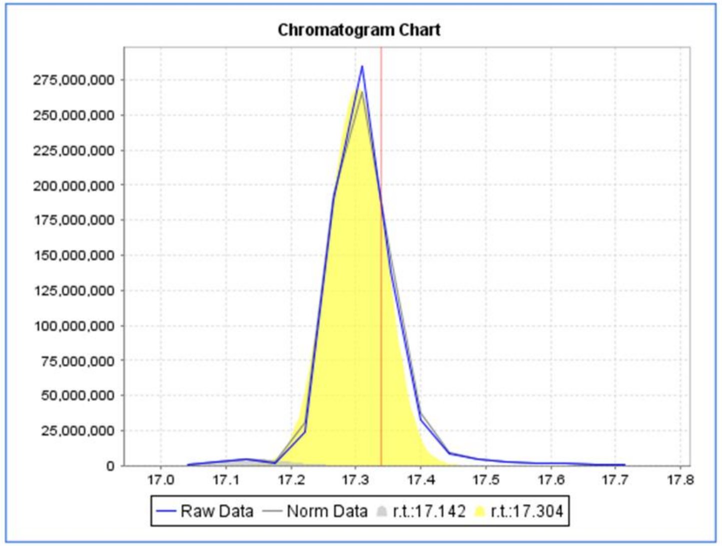

Spectrum

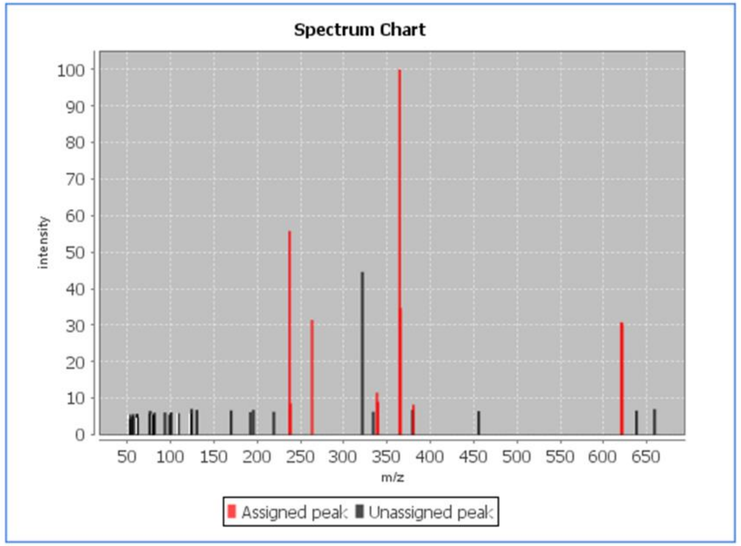

Ceramides (Cer)

Information

- ➢ Ionization**:** Negative-ion mode
- ➢ Main ion adducts used for identification**:** $[M+HCOO]^-$ and $[M\text{-}H]^-$
- ➢ Identification (fragmentation) grade**:** A , B and C
- ➢ Main ion adduct used for quantification**:** $[M+HCOO]^-$

Match Lipid

LipidIon	M-Sc.	T-Sc.	Occ.	St.
◉ **Cer(d18:1_22:0)+HCOO**	**35.7**	**0**	**59.5**	⊠
○ Cer(d16:0_24:1)+HCOO	19.9	0	49.8	⊠
○ Cer(d15:0_25:1)+HCOO	7.3	0	36.4	⊠
○ Cer(d15:1_25:0)+HCOO	7.3	0	36.4	⊠
○ Cer(d18:0_22:1)+HCOO	6.4	0	21.2	⊠
○ Cer(d24:0_16:1)+HCOO	4.7	0	23.3	⊠
○ Cer(d22:0_18:1)+HCOO	3.9	0	19.7	⊠
○ Cer(d24:1_16:0)+HCOO	3.9	0	19.7	⊠
○ Cer(d17:1_23:0)+HCOO	3	0	15.2	⊠
○ Cer(m18:1_22:0+O)+HCOO	2.6	0	12.9	⊠

Match Detail

ObsMz	Type	It.(%)	Frag.	Delta(Da)
195.5036	MS2	6.801	-	-
218.9571	MS2	6.297	-	-
237.2228	MS2	55.795	Frag[d18:1]-H	0.0004
238.2263	MS2	8.554	Frag[d18:1]-H [i	1.004
263.2387	MS2	31.44	NL[H2O,FAmide	0.0006
321.3167	MS2	44.586	-	-
333.8749	MS2	6.308	-	-
338.3446	MS2	11.552	FAmide(22:0)-H	0.0017
339.3287	MS2	8.98	FAmide(22:0)-H	0.9859
364.36	MS2	100	NL[d18:1,H2O]	0.0015
365.363	MS2	34.747	NL[d18:1,H2O]	1.0046
379.3787	MS2	6.797	-	-
380.3534	MS2	8.25	NL[d18:1,H2]	0
455.8556	MS2	6.503	-	-
620.6018	MS2	30.8	M-H	0.0031

Copy

Representation of ceramides (Cer) identification information obtained with LipidSearch software e.g. $[Cer(d40:1)+HCOO]^-$

Chromatogram

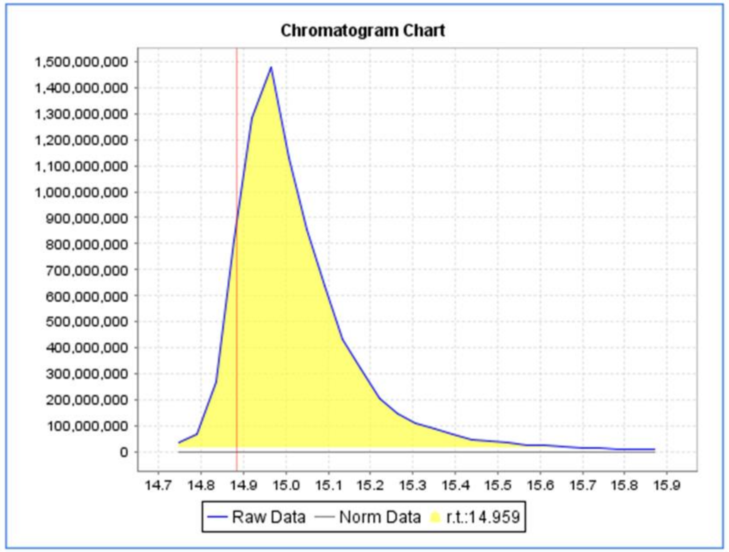

Spectrum

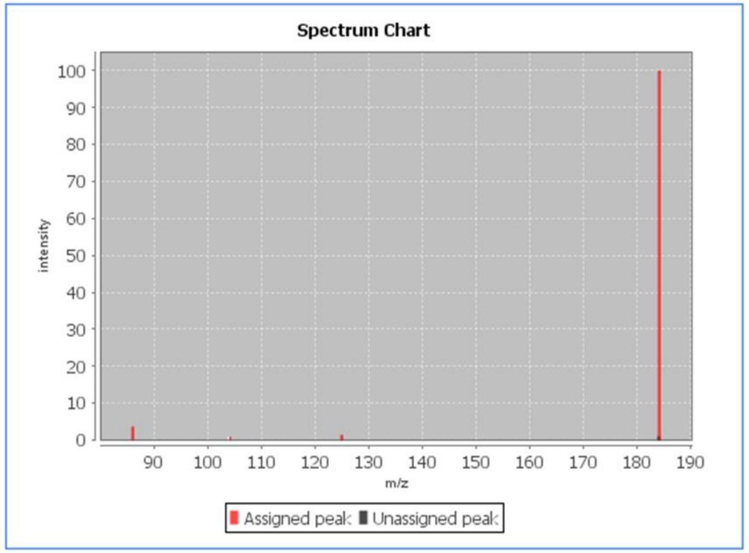

Match Lipid

LipidIon	M-Sc.	T-Sc.	Occ.	St.
SM(d16:0_24:1)+H	39.6	0.9	99	
SM(d16:1_24:0)+H	39.6	0.9	99	
SM(d17:0_23:1)+H	39.6	0.9	99	
SM(d17:1_23:0)+H	39.6	0.9	99	
SM(d18:0_22:1)+H	39.6	0.9	99	
**SM(d18:1_22:0)+H**	**39.6**	**0.9**	**99**	
SM(d19:0_21:1)+H	39.6	0.9	99	
SM(d19:1_21:0)+H	39.6	0.9	99	
SM(d20:0_20:1)+H	39.6	0.9	99	
SM(d20:1_20:0)+H	39.6	0.9	99	

Match Detail

ObsMz	Type	It.(%)	Frag.	Delta(Da)
86.097	MS2	3.697	C5H12N1	0.0006
104.1073	MS2	1.009	C5H14N1O1	0.0003
125	MS2	1.469	C2H6O4P1	0.0001
184.0037	MS2	1.098	-	-
184.0733	MS2	100	(P-Cho)+H	0

Copy

Sphingomyelin (SM)

Information

- Ionization: Positive- and negative-ion mode
- Main ion adducts used for identification: $[M+H]^+$, $[M+Na]^+$ and $[M+HCOO]^-$
- Identification (fragmentation) grade: A , B and C
- Main ion adduct used for quantification: $[M+H]^+$

Representation of sphingomyelin (SM) identification information obtained with LipidSearch software
e.g. $[SM(d40:1)+H]^+$

Chromatogram

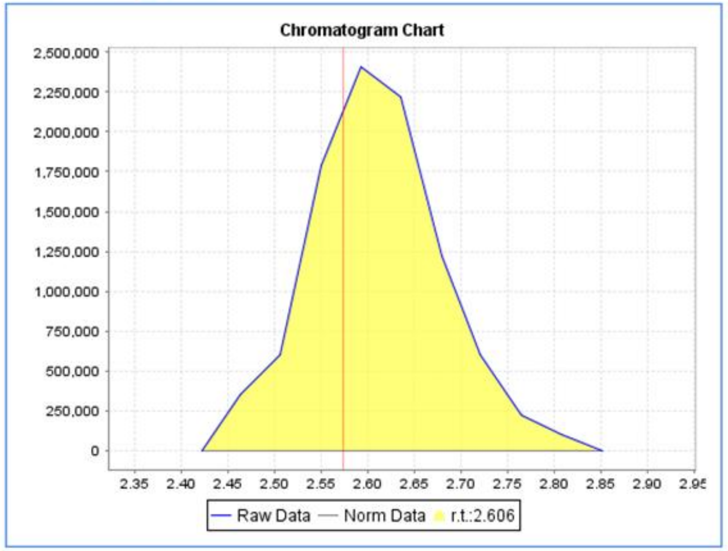

Spectrum

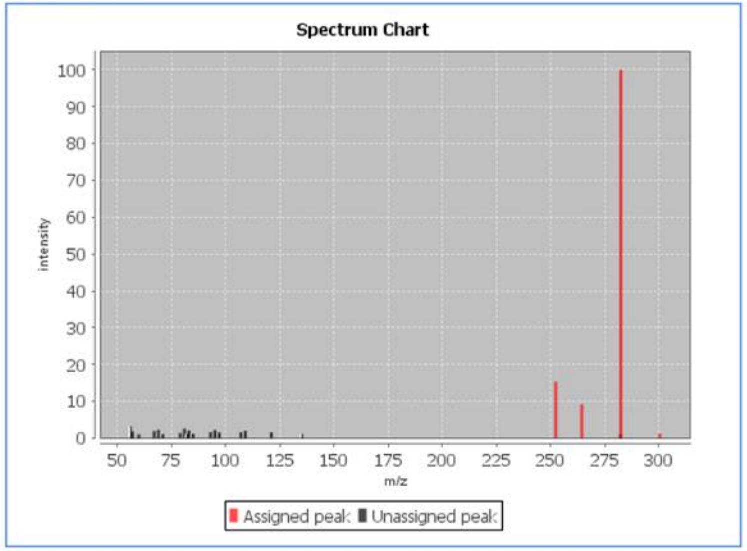

Match Lipid

LipidIon	M-Sc.	T-Sc.	Occ.	St.
SPH(d18:1)+H	23.7	0.5	78.9	
SPH(t18:0)+H-H2O	23.7	0.8	78.9	

Match Detail

ObsMz	Type	It.(%)	Frag.	Delta(Da)
82.0659	MS2	1.17	-	-
83.0863	MS2	2.128	-	-
85.1019	MS2	1.221	-	-
93.0705	MS2	1.686	-	-
95.0861	MS2	2.244	-	-
97.1018	MS2	1.607	-	-
107.0861	MS2	1.629	-	-
109.1017	MS2	2.036	-	-
121.1015	MS2	1.62	-	-
135.1171	MS2	1.154	-	-
252.269	MS2	15.355	NL[CH2O+H2O	0.0005
264.269	MS2	9.23	NL[2H2O]	0.0004
282.1546	MS2	1.064	-	-
282.2797	MS2	100	NL[H2O]	0.0006
300.2906	MS2	1.239	M+H	0.0009

Copy

Sphingosine (SPH)

Information

- Ionization: Positive-ion mode
- Main ion adducts used for identification: $[M+H]^+$ and $[M+H-H_2O]^+$
- Identification (fragmentation) grade: A , B and C
- Main ion adduct used for quantification: $[M+H]^+$

Representation of sphingosine (SPH) identification information obtained with LipidSearch software e.g. $[SPH(d18:1)+H]^+$

Chromatogram

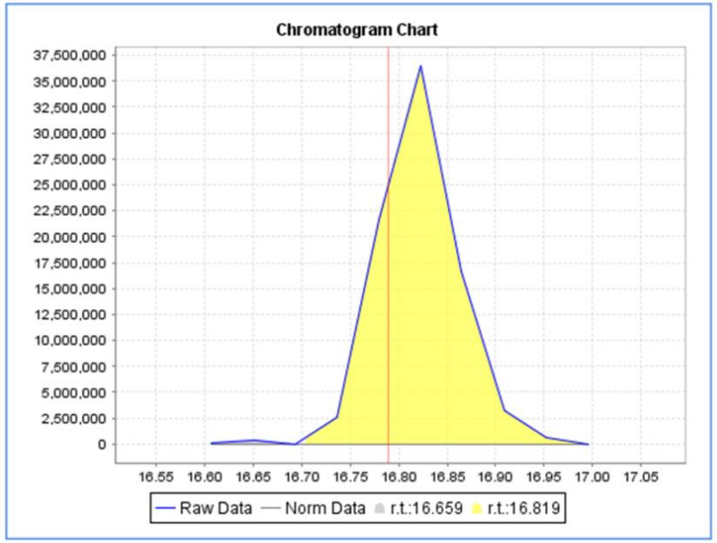

Spectrum

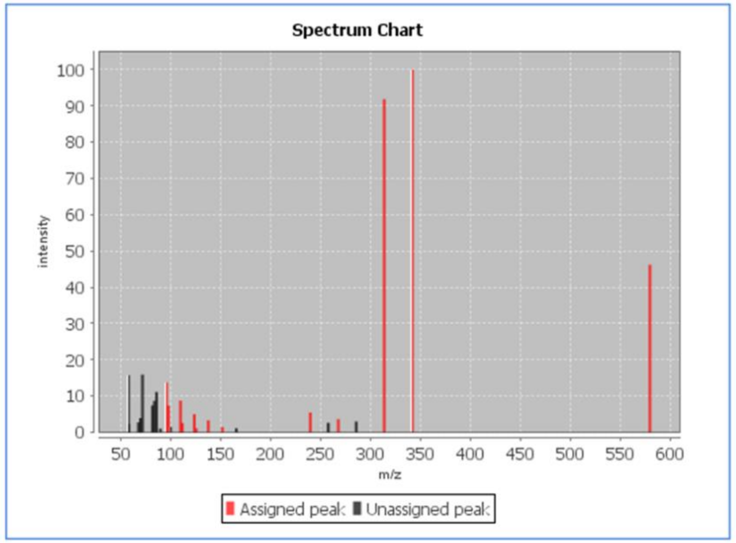

Match Lipid

LipidIon	M-Sc.	T-Sc.	Occ.	St.
◉ **DG(18:0_16:0)+NH4**	**40.1**	**0.3**	**79**	
○ TG(12:0e_6:0_16:0)+NH4	16.3	0.3	53	
○ DG(10:0_24:0)+NH4	2.6	0.3	24.3	
○ DG(11:0_23:0)+NH4	2.6	0.3	24.3	
○ DG(12:0_22:0)+NH4	2.6	0.3	24.3	
○ DG(13:0_21:0)+NH4	2.6	0.3	24.3	
○ DG(20:0_14:0)+NH4	2.6	0.3	24.3	
○ DG(15:0_19:0)+NH4	2.6	0.3	24.3	
○ DG(17:0_17:0)+NH4	2.6	0.3	24.3	
○ DG(26:0_8:0)+NH4	2.6	0.3	24.3	
○ DG(28:0_6:0)+NH4	2.6	0.3	24.3	
○ TG(12:0e_8:0_14:0)+NH4	2.6	0.3	24.3	
○ TG(12:0e_10:0_12:0)+NH4	2.6	0.3	24.3	

Match Detail

ObsMz	Type	It.(%)	Frag.	Delta(Da)
99.1174	MS2	1.52	-	-
109.1016	MS2	8.773	C8H13	0.0005
111.1173	MS2	2.568	C8H15	0.0005
123.1172	MS2	4.979	C9H15	0.0003
125.1328	MS2	1.117	C9H17	0.0003
137.1326	MS2	3.304	C10H17	0.0002
151.1482	MS2	1.463	C11H19	0.0001
165.164	MS2	1.116	-	-
239.2372	MS2	5.447	FA(16:0)-OH	0.0003
257.2477	MS2	2.623	-	-
267.2688	MS2	3.644	FA(18:0)-OH	0.0006
285.279	MS2	2.978	-	-
313.2743	MS2	91.874	NL[FA(18:0)-H+	0.0005
341.3055	MS2	100	NL[FA(16:0)-H+	0.0005
579.5352	MS2	46.208	M-OH	0.0005

Copy

Diglyceride (DG)

Information

- Ionization**:** Positive-ion mode
- Main ion adducts used for identification**:** $[M+Na]^+$, $[M+NH_4]^+$, $[M+H]^+$ and $[M+K]^+$
- Identification (fragmentation) grade**:** A and B
- Main ion adducts used for quantification**:** $[M+NH_4]^+$ and $[M+Na]^+$

Representation of diglyceride (DG) identification information obtained with LipidSearch software e.g. $[DG(34:0)+NH_4]^+$

Chromatogram

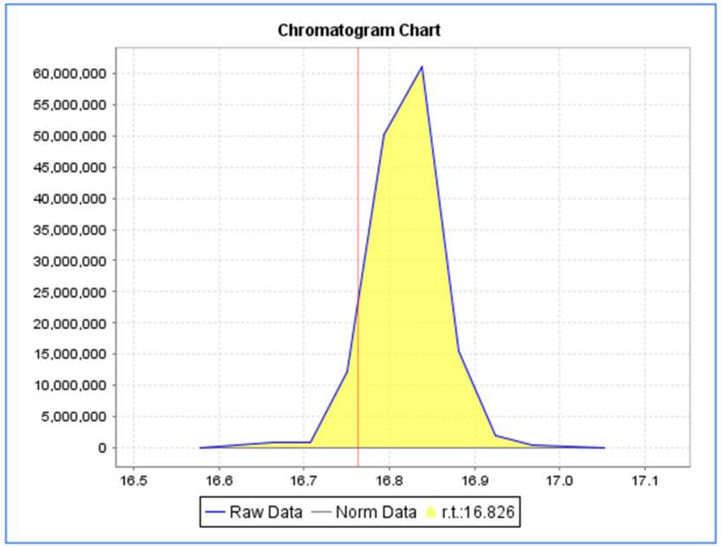

Spectrum

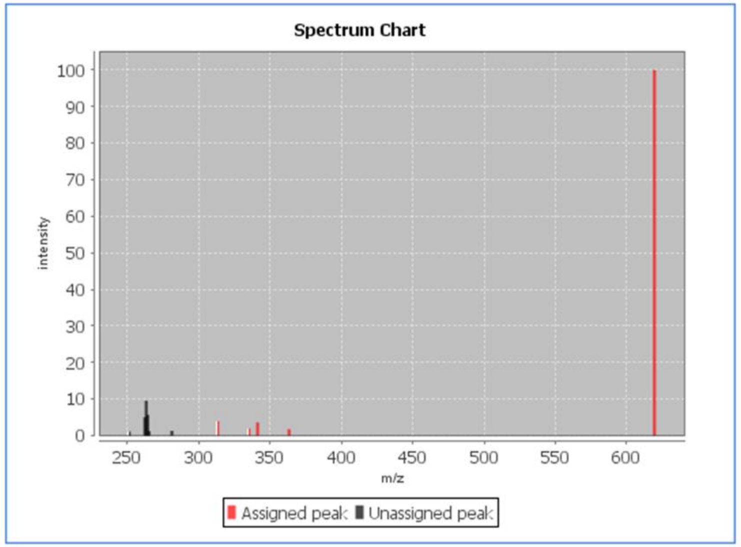

Match Lipid

LipidIon	M-Sc.	T-Sc.	Occ.	St.
DG(18:0_16:0)+Na	12.8	0.8	32.1	
DG(16:0_20:3)+H	3.3	1.6	16.3	
DG(18:0_18:3)+H	3.1	1.6	15.7	
TG(12:0e_6:0_16:0)+Na	3.1	0.8	15.4	
TG(12:0e_6:0_18:3)+H	2.1	1.6	10.3	

Match Detail

ObsMz	Type	It.(%)	Frag.	Delta(Da)
251.2563	MS2	1.115	-	-
262.2532	MS2	5.038	-	-
263.2568	MS2	9.553	-	-
264.2603	MS2	5.706	-	-
265.263	MS2	1.215	-	-
281.2671	MS2	1.297	-	-
313.2745	MS2	3.979	NL[FA(18:0)-H+	0.0007
335.2566	MS2	1.897	NL[FA(18:0)]	0.001
341.3055	MS2	3.64	NL[FA(16:0)-H+	0.0005
363.288	MS2	1.777	NL[FA(16:0)]	0.001
619.5273	MS2	100	M+Na	0.0001

Copy

Diglyceride (DG)

Information

- Ionization: Positive-ion mode
- Main ion adducts used for identification: $[M+Na]^+$, $[M+NH_4]^+$, $[M+H]^+$ and $[M+K]^+$
- Identification (fragmentation) grade: A and B
- Main ion adducts used for quantification: $[M+Na]^+$ and $[M+NH_4]^+$

Representation of diglyceride (DG) identification information obtained with LipidSearch software e.g. $[DG(34:0)+Na]^+$

Chromatogram

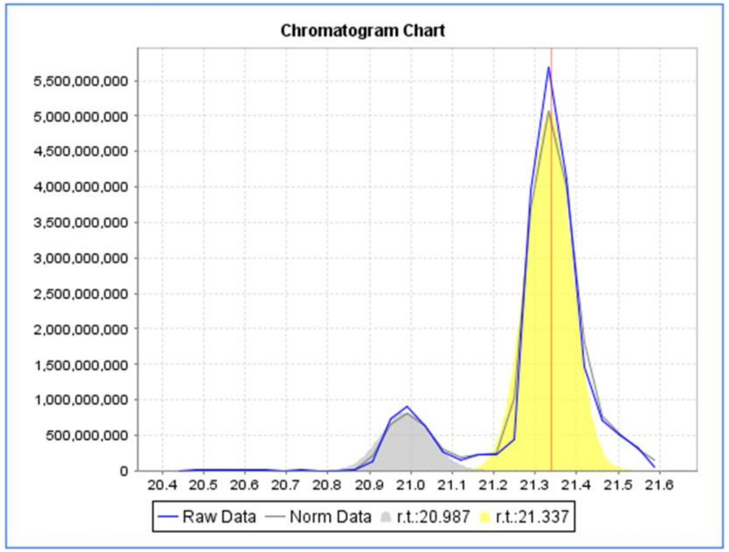

Spectrum

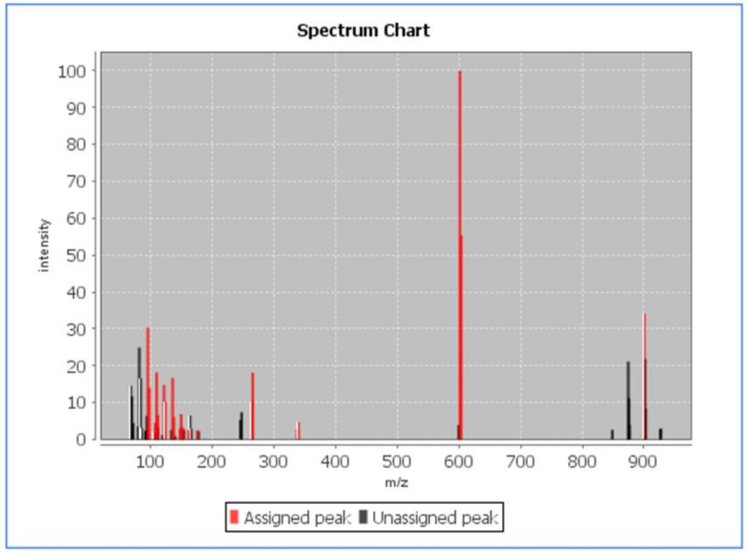

Match Lipid

LipidIon	M-Sc.	T-Sc.	Occ.	St.
TG(18:1_18:1_18:2)+NH4	58.7	0.1	64.2	
TG(18:0_18:2_18:2)+NH4	29.4	0.1	41.2	
TG(18:0_18:1_18:3)+NH4	21.4	0.1	51.7	
TG(26:1_10:1_18:2)+NH4	16.9	0.1	40.9	
TG(16:0_18:1_20:3)+NH4	16	0.1	51	
TG(16:1_18:1_20:2)+NH4	16	0.1	51	

Match Detail

ObsMz	Type	It.(%)	Frag.	Delta(Da)
245.2268	MS2	5.321	-	-
247.2429	MS2	7.425	-	-
263.2376	MS2	10.285	FA(18:2)-OH	0.0007
265.2533	MS2	18.099	FA(18:1)-OH	0.0007
337.2746	MS2	2.817	MG(18:2)-OH	0.0009
339.2901	MS2	4.735	MG(18:1)-OH	0.0007
599.5042	MS2	3.917	-	-
601.5206	MS2	100	NL[FA(18:1)-H+NH4]	0.0016
603.5363	MS2	55.323	NL[FA(18:2)-H+NH4]	0.0017
848.7728	MS2	2.673	-	-
874.7882	MS2	21.143	-	-
875.7919	MS2	11.172	-	-
876.7922	MS2	3.992	-	-
900.8035	MS2	34.146	M+NH4	0.0021

Copy

Triglyceride (TG)

Information

- Ionization: Positive-ion mode
- Main ion adducts used for identification: $[M+NH_4]^+$, $[M+Na]^+$, $[M+H]^+$ and $[M+K]^+$
- Identification (fragmentation) grade: A and B
- Main ion adduct used for quantification: $[M+NH_4]^+$

Representation of triglyceride (TG) identification information obtained with LipidSearch software
e.g. $[TG(54:4)+NH_4]^+$

Chromatogram

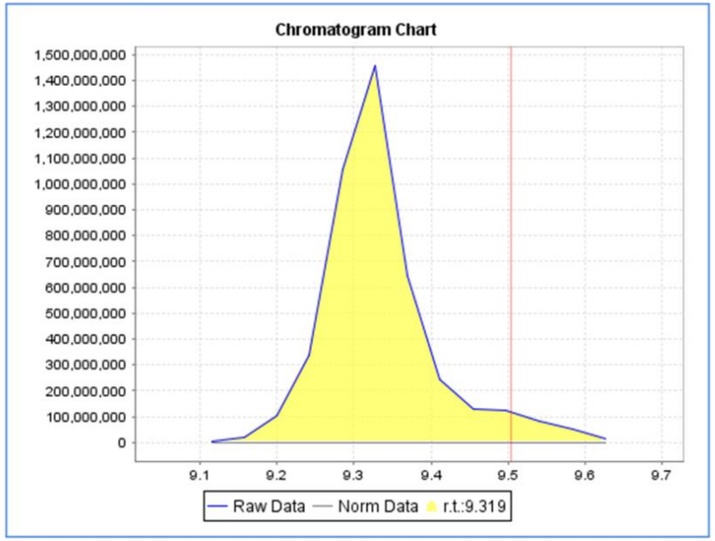

Spectrum

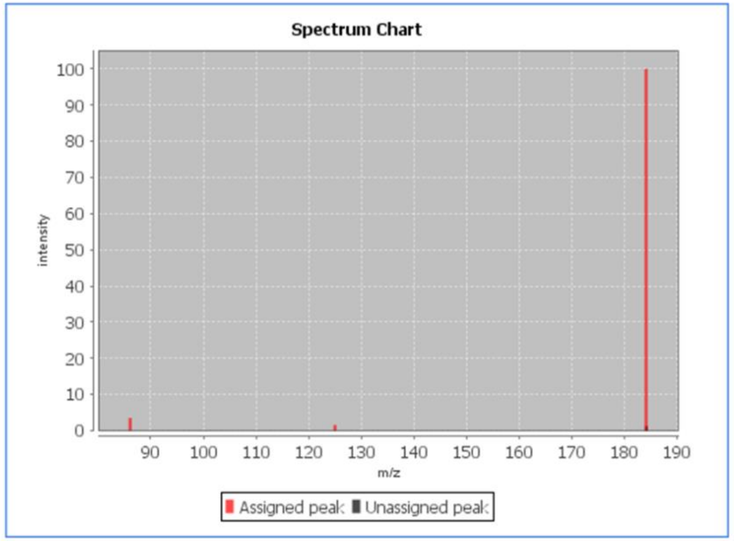

Match Lipid

LipidIon	M-Sc.	T-Sc.	Occ.	St.
PC(8:0_24:1)+H	29.7	1	98.8	
PC(9:0_23:1)+H	29.7	1	98.8	
PC(10:0_22:1)+H	29.7	1	98.8	
PC(11:0_21:1)+H	29.7	1	98.8	
PC(20:1_12:0)+H	29.7	1	98.8	
PC(19:1_13:0)+H	29.7	1	98.8	
PC(18:1_14:0)+H	29.7	1	98.8	
PC(17:1_15:0)+H	29.7	1	98.8	
**PC(16:0_16:1)+H**	**29.7**	**1**	**98.8**	
PC(18:0_14:1)+H	29.7	1	98.8	
PC(20:0_12:1)+H	29.7	1	98.8	
PC(22:0_10:1)+H	29.7	1	98.8	
PC(26:1_6:0)+H	29.7	1	98.8	

Match Detail

ObsMz	Type	It.(%)	Frag.	Delta(Da)
86.0971	MS2	3.497	C5H12N	0.0007
125.0001	MS2	1.568	C2H6O4P1	0.0002
184.0735	MS2	100	(P-Cho)+H	0.0002
184.1413	MS2	1.274	-	-

Copy

Phosphatidylcholine (PC)

Information

- Ionization: Positive-ion mode
- Main ion adducts used for identification: $[M+H]^+$, $[M+Na]^+$ and $[M+K]^+$
- Identification (fragmentation) grade: A and B
- Main ion adduct used for quantification: $[M+H]^+$

Representation of phosphatidylcholine (PC) identification information obtained with LipidSearch software e.g. $[PC(32:1)+H]^+$

Phosphatidylcholine (PC)

Chromatogram

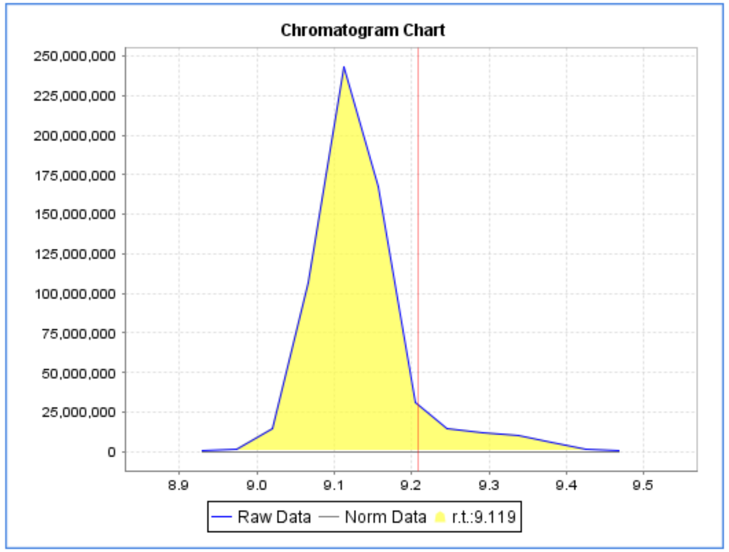

Spectrum

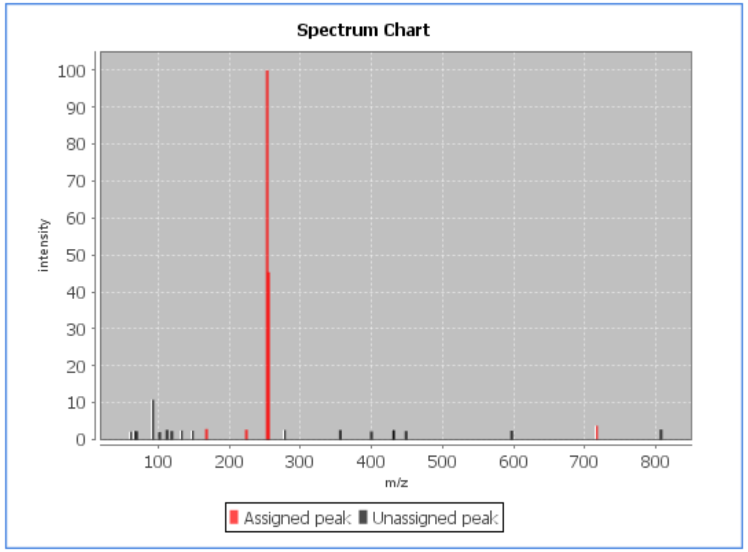

Match Lipid

LipidIon	M-Sc.	T-Sc.	Occ.	St.
◉ **PC(16:0_16:1)+HCOO**	**37.9**	**0.2**	**75.7**	
○ PS(16:0_19:0)-H	2.2	3.9	22.2	

Match Detail

ObsMz	Type	It.(%)	Frag.	Delta(Da)
132.4829	MS2	2.5	-	-
148.0111	MS2	2.442	-	-
168.0422	MS2	2.92	(P-Cho)-CH3-H	-0.001
224.0696	MS2	2.699	M-FA1-FA2+H2O-CH3	0.0002
253.2175	MS2	100	FA(16:1)-H	0.0002
255.2332	MS2	45.333	FA(16:0)-H	0.0002
277.44	MS2	2.631	-	-
356.3354	MS2	2.599	-	-
400.0869	MS2	2.231	-	-
430.5109	MS2	2.553	-	-
431.0115	MS2	2.515	-	-
448.7605	MS2	2.318	-	-
597.5173	MS2	2.442	-	-
716.5259	MS2	3.786	M-CH3	0.0023

Copy

Information

- Ionization: Negative-ion mode
- Main ion adducts used for identification: $[M+HCOO]^-$ and $[M\text{-}CH_3]^-$
- Identification (fragmentation) grade: A and B
- Main ion adduct used for quantification: $[M+HCOO]^-$

Representation of phosphatidylcholine (PC) identification information obtained with LipidSearch software e.g. $[PC(32{:}1)+HCOO]^-$

Chromatogram

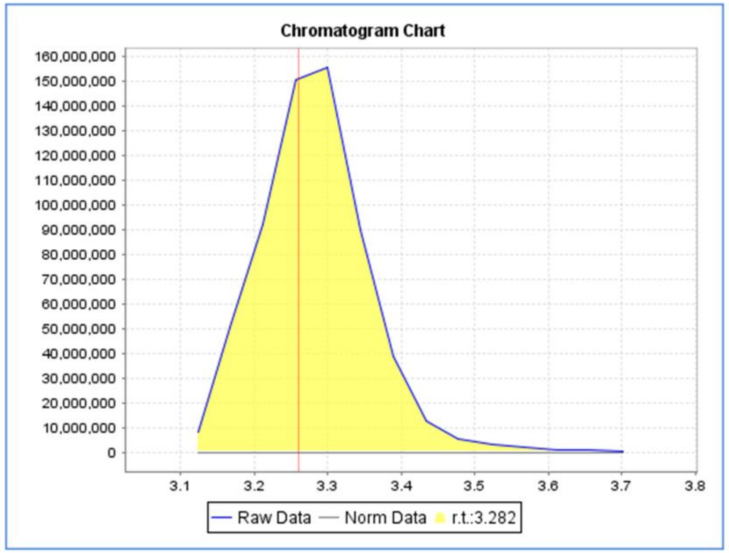

Spectrum

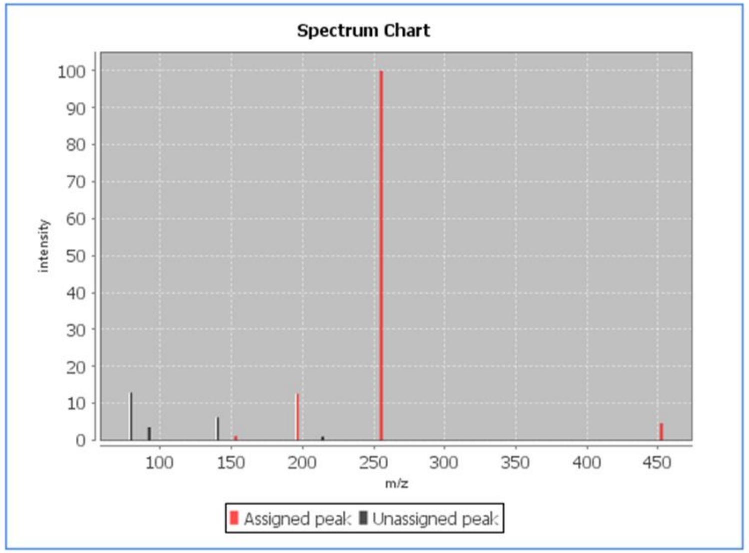

Match Lipid

LipidIon	M-Sc.	T-Sc.	Occ.	St.
LPE(16:0)-H	24.8	0.4	82.7	

Match Detail

ObsMz	Type	It.(%)	Frag.	Delta(Da)
78.9573	MS2	12.949	-	-
92.1398	MS2	3.562	-	-
140.0104	MS2	6.261	-	-
152.9947	MS2	1.16	GP-H3O	-0.0011
196.0372	MS2	12.623	M-FA(16:0)-H	-0.0008
214.0479	MS2	1.048	-	-
255.233	MS2	100	FA(16:0)-H	0
452.2785	MS2	4.611	M-H	0.0002

Copy

Lysophosphatidylethanolamine (LPE)

Information

- Ionization**:** Negative-ion mode
- Main ion adduct used for identification**:** $[M\text{-}H]^-$
- Identification (fragmentation) grade**:** A and B
- Main ion adduct used for quantification**:** $[M\text{-}H]^-$

Representation of lysophosphatidylethanolamine (LPE) identification information obtained with LipidSearch software
e.g. $[LPE(16:0)\text{-}H]^-$

Chromatogram

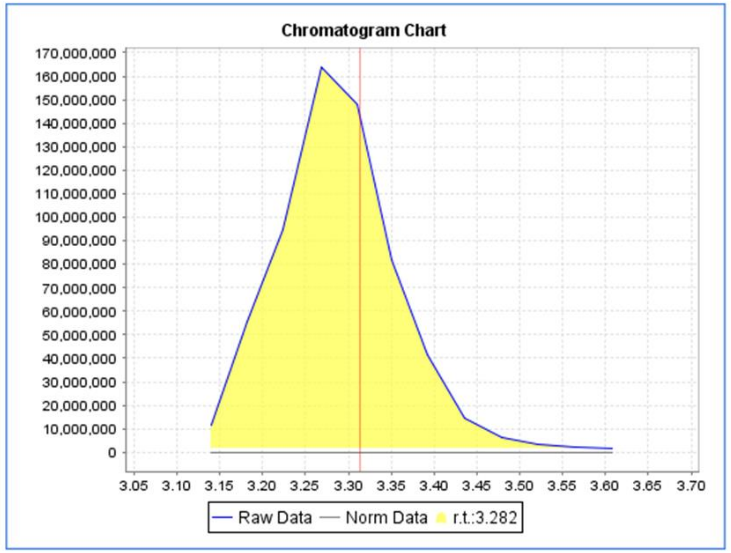

Spectrum

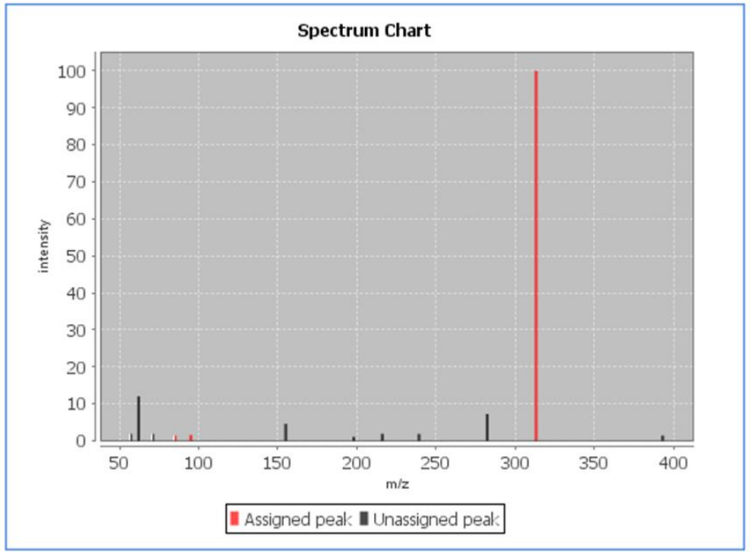

Lysophosphatidylethanolamine (LPE)

<u>Information</u>

- Ionization**:** Positive-ion mode
- Main ion adducts used for identification**:** $[M+H]^+$ and $[M+Na]^+$
- Identification (fragmentation) grade**:** A and B
- Main ion adduct used for quantification**:** $[M+H]^+$

Match Lipid

LipidIon	M-Sc.	T-Sc.	Occ.	St.
◉ LPE(16:0)+H	7.7	0.1	75.1	⊠
○ PE(8:0e_8:0)+H	7.7	0.1	75.1	⊠
○ PE(10:0e_6:0)+H	7.7	0.1	75.1	⊠

Match Detail

ObsMz	Type	It.(%)	Frag.	Delta(Da)
57.0709	MS2	1.906	-	-
62.061	MS2	12.064	-	-
71.0865	MS2	1.912	-	-
85.1018	MS2	1.44	C6H13	0.0007
95.0861	MS2	1.586	C7H11	0.0006
155.0107	MS2	4.634	-	-
198.0529	MS2	1.089	-	-
216.0636	MS2	1.901	-	-
239.2374	MS2	1.886	-	-
282.2799	MS2	7.291	-	-
313.2746	MS2	100	NL[PE]	0.0009
393.2404	MS2	1.477	-	-

Copy

Representation of lysophosphatidylethanolamine (LPE) identification information obtained with LipidSearch software
e.g. $[LPE(16:0)+H]^+$

Phosphatidylethanolamine (PE)

<u>Information</u>

- Ionization**:** Positive-ion mode
- Main ion adducts used for identification**:** $[M+H]^+$ and $[M+Na]^+$
- Identification (fragmentation) grade**:** A and B
- Main ion adduct used for quantification**:** $[M+H]^+$

Representation of phosphatidylethanolamine (PE) identification information obtained with LipidSearch software
e.g. $[PE(36:2)+H]^+$

Chromatogram

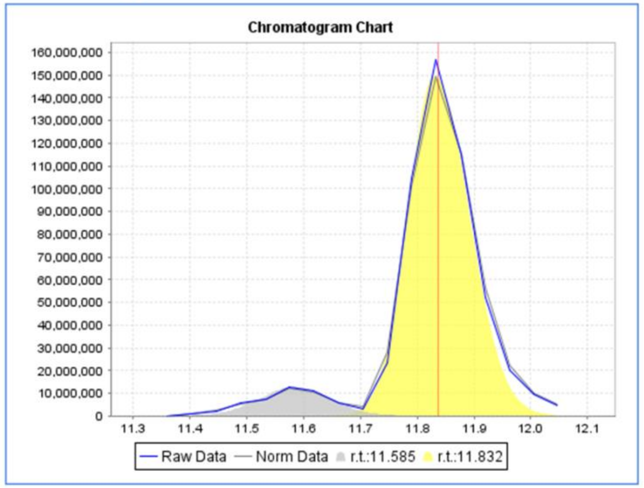

Spectrum

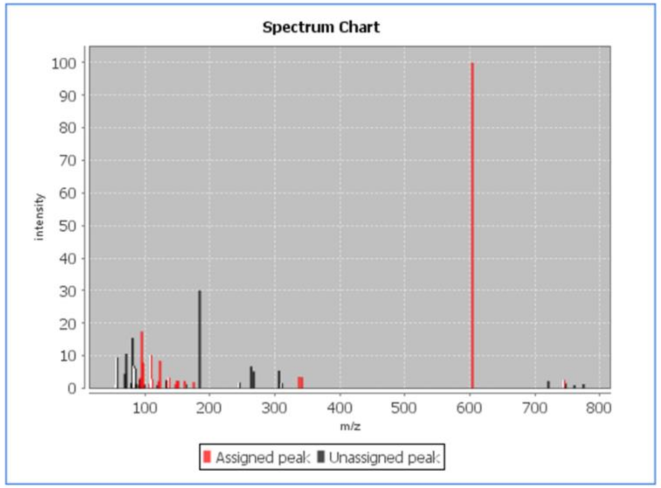

Match Lipid

LipidIon	M-Sc.	T-Sc.	Occ.	St.
◉ PE(18:0_18:2)+H	18.5	0.2	58.8	
○ PC(15:0_18:2)+H	7.4	3	34.4	
○ PE(12:0_24:2)+H	6.5	0.2	56.5	
○ PE(14:0_22:2)+H	6.5	0.2	56.5	
○ PE(16:0_20:2)+H	6.5	0.2	56.5	
○ PE(16:1_20:1)+H	6.5	0.2	56.5	
○ PE(17:1_19:1)+H	6.5	0.2	56.5	
○ PE(18:1_18:1)+H	6.5	0.2	56.5	
○ PE(22:0_14:2)+H	6.5	0.2	56.5	
○ PE(22:1_14:1)+H	6.5	0.2	56.5	
○ PE(24:0_12:2)+H	6.5	0.2	56.5	
○ PE(24:1_12:1)+H	6.5	0.2	56.5	
○ PE(26:0_10:2)+H	6.5	0.2	56.5	
○ PE(25:0_11:2)+H	6.5	0.2	56.5	
○ PE(25:1_11:1)+H	6.5	0.2	56.5	
○ PE(26:1_10:1)+H	6.5	0.2	56.5	

Match Detail

ObsMz	Type	It.(%)	Frag.	Delta(Da)
151.1485	MS2	2.341	C11H19	0.0004
161.1329	MS2	2.258	C12H17	0.0004
163.1485	MS2	1.28	-	-
175.1486	MS2	1.942	C13H19	0.0005
184.0737	MS2	30.077	-	-
245.2269	MS2	1.758	-	-
263.2376	MS2	6.749	-	-
267.2688	MS2	5.231	-	-
306.28	MS2	5.466	-	-
310.3115	MS2	1.585	-	-
337.2746	MS2	3.55	MG(18:2)-OH	0.0008
341.3056	MS2	3.444	MG(18:0)-OH	0.0006
603.5358	MS2	100	NL[PE]	0.0011
720.5909	MS2	2.228	-	-
744.5525	MS2	2.598	M+H	-0.0013

Copy

ObsMz	Type	It.(%)	Frag.	Delta(Da)
55.0552	MS2	1.147		
57.0709	MS2	9.491		
69.0708	MS2	4.477		
71.0864	MS2	10.618		
79.0551	MS2	1.611		
81.0707	MS2	15.508		
83.0863	MS2	6.949		
85.102	MS2	6.121		
86.0972	MS2	1.401		
91.0548	MS2	1.14		
92.1468	MS2	2.722		
93.0706	MS2	3.286	C7H9	0.0007
95.0862	MS2	17.492	C7H11	0.0007
97.1019	MS2	7.943	C7H13	0.0007
99.0811	MS2	1.155		
107.0861	MS2	1.46	C8H11	0.0006
109.1018	MS2	10.269	C8H13	0.0006
111.1174	MS2	2.869	C8H15	0.0006
119.0862	MS2	1.047		
121.1018	MS2	2.253	C9H13	0.0006
123.1172	MS2	8.486	C9H15	0.0004
133.1016	MS2	2.545		
135.1172	MS2	2.476	C10H15	0.0004
137.1328	MS2	3.336	C10H17	0.0003
147.1172	MS2	1.258	C11H15	0.0003
149.1328	MS2	2.355	C11H17	0.0003

Chromatogram

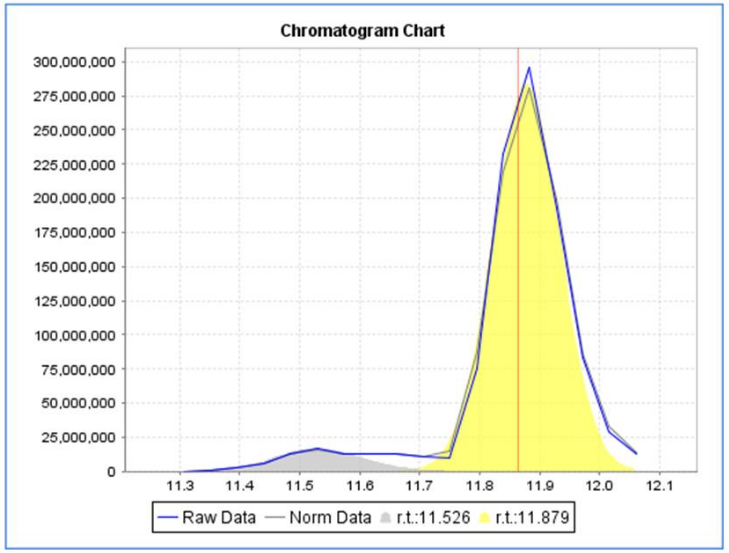

Spectrum

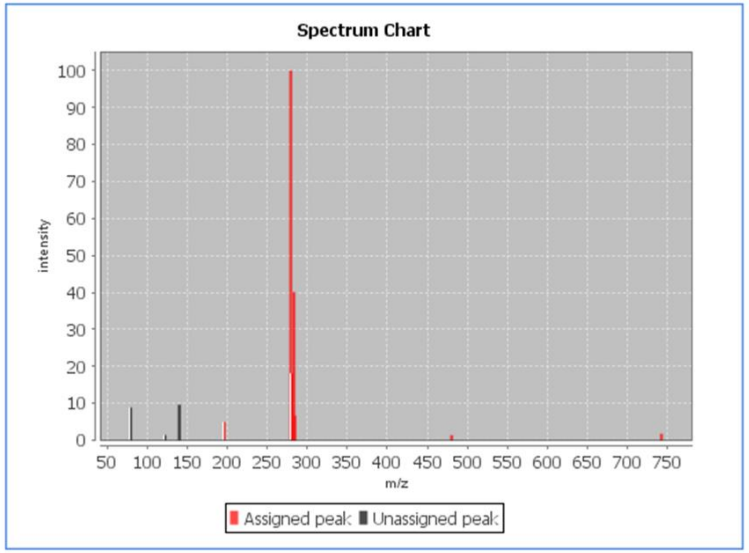

Phosphatidylethanolamine (PE)

Information

- Ionization**:** Negative-ion mode
- Main ion adduct used for identification**:** $[M\text{-}H]^-$
- Identification (fragmentation) grade**:** A and B
- Main ion adduct used for quantification**:** $[M\text{-}H]^-$

Match Lipid

LipidIon	M-Sc.	T-Sc.	Occ.	St.
**PE(18:0_18:2)-H**	**35.8**	**0.3**	**89.5**	
dMePE(16:0_18:2)-H	12.5	2.3	62.5	
PC(16:0_18:2)-CH3	12.5	2.4	62.5	

Match Detail

ObsMz	Type	It.(%)	Frag.	Delta(Da)
78.9572	MS2	8.905	-	-
121.9995	MS2	1.463	-	-
140.0103	MS2	9.641	-	-
196.0371	MS2	4.923	M-FA1-FA2+H2O-H	-0.0009
279.2329	MS2	100	FA(18:2)-H	0
280.2363	MS2	18.164	FA(18:2)-H [isotope]	1.0033
283.2642	MS2	40.118	FA(18:0)-H	-0.0001
284.2677	MS2	6.708	FA(18:0)-H [isotope]	1.0035
480.3096	MS2	1.391	LPE(18:0)-H	0
742.5389	MS2	1.778	M-H	-0.0004

Copy

Representation of phosphatidylethanolamine (PE) identification information obtained with LipidSearch software
e.g. $[PE(36:2)\text{-}H]^-$

Phosphatidylglycerol (PG)

Chromatogram

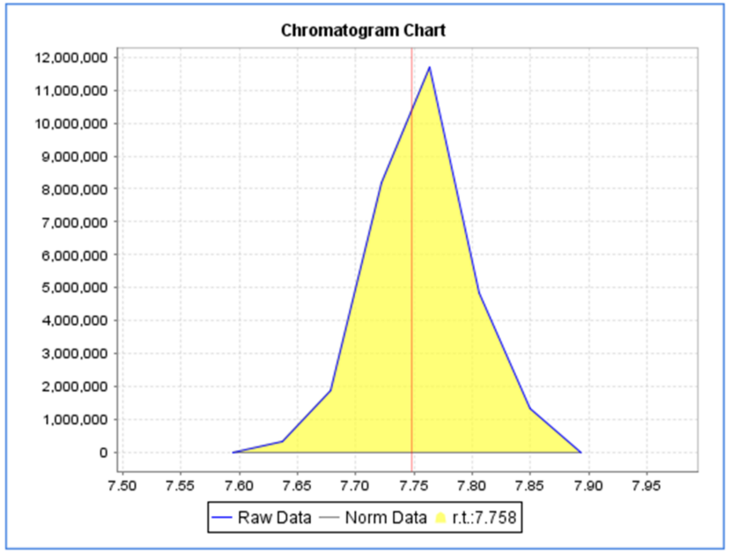

Spectrum

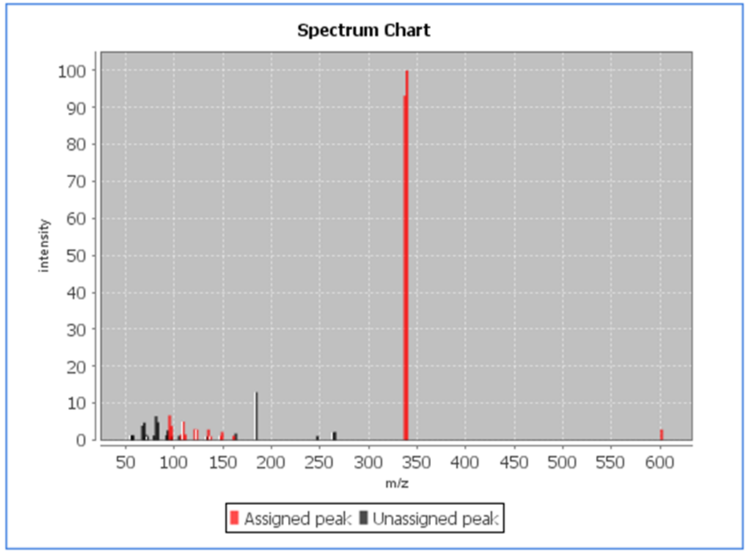

Match Lipid

LipidIon	M-Sc.	T-Sc.	Occ.	St.
PG(18:1_18:2)+NH4	25.1	2.3	80.6	
PS(18:0_18:1)+H	5.2	9.1	46.8	

Match Detail

ObsMz	Type	It.(%)	Frag.	Delta(Da)
123.1172	MS2	2.902	C9H15	0.0004
133.1014	MS2	1.043	-	-
135.1171	MS2	2.909	C10H15	0.0003
137.1328	MS2	1.088	C10H17	0.0003
147.1171	MS2	1.188	C11H15	0.0002
149.1328	MS2	2.24	C11H17	0.0004
161.1329	MS2	1.197	C12H17	0.0004
163.1483	MS2	1.804	-	-
184.0737	MS2	13.011	-	-
247.2427	MS2	1.124	-	-
263.2375	MS2	2.107	-	-
265.2532	MS2	2.25	-	-
337.2745	MS2	93.162	MG(18:2)-OH	0.0008
339.2902	MS2	100	MG(18:1)-OH	0.0008
601.5166	MS2	2.903	NL[PG,+NH4]+H	-0.0025

Copy

Information

- Ionization: Positive-ion mode
- Main ion adducts used for identification: $[M+NH_4]^+$, $[M+H]^+$ and $[M+Na]^+$
- Identification (fragmentation) grade: A and B
- Main ion adduct used for quantification: $[M+NH_4]^+$

Representation of phosphatidylglycerol (PG) identification information obtained with LipidSearch software e.g. $[PG(36:3)+NH_4]^+$

ObsMz	Type	It.(%)	Frag.	Delta(Da)
55.0552	MS2	1.308		
57.0345	MS2	1.18		
57.0709	MS2	1.428		
67.0551	MS2	3.905		
69.0707	MS2	4.744		
71.0864	MS2	1.462		
72.0816	MS2	1.034		
79.0551	MS2	1.282		
81.0707	MS2	6.478		
83.0862	MS2	4.832		
92.1458	MS2	1.347		
93.0706	MS2	2.643		
95.0862	MS2	6.72	C7H11	0.0006
97.0654	MS2	1.085		
97.1018	MS2	3.848	C7H13	0.0006
105.0704	MS2	1.191		
107.0862	MS2	1.538	C8H11	0.0006
109.1017	MS2	5.017	C8H13	0.0005
111.1174	MS2	1.549	C8H15	0.0006
121.1016	MS2	3.011	C9H13	0.0004

Chromatogram

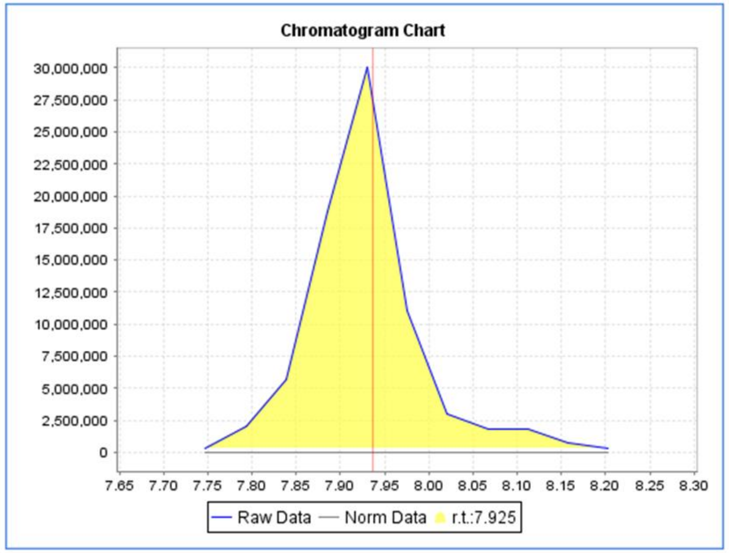

Spectrum

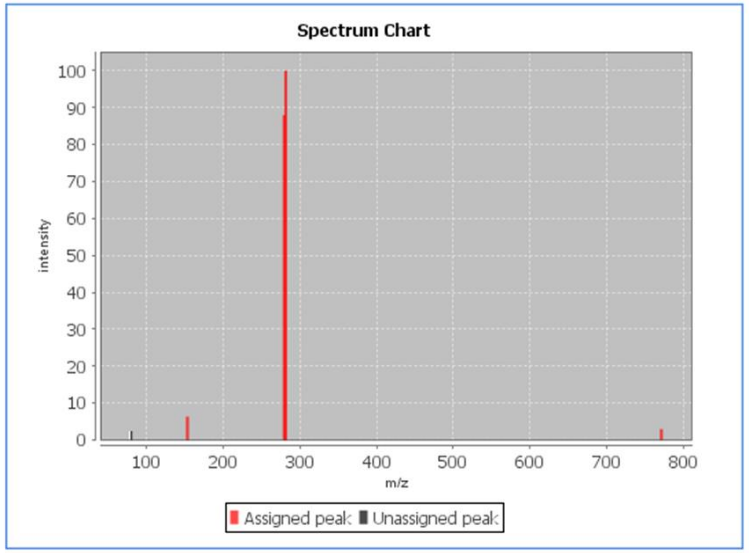

Match Lipid

LipidIon	M-Sc.	T-Sc.	Occ.	St.
PG(18:1_18:2)-H	29.6	1.5	98.8	

Match Detail

ObsMz	Type	It.(%)	Frag.	Delta(Da)
78.9574	MS2	2.434	-	-
152.9947	MS2	6.327	GP-H3O	-0.0012
279.2334	MS2	87.976	FA(18:2)-H	0.0004
281.249	MS2	100	FA(18:1)-H	0.0004
771.5175	MS2	2.927	M-H	-0.0006

Copy

Phosphatidylglycerol (PG)

Information

- Ionization**:** Negative-ion mode
- Main ion adduct used for identification**:** $[M\text{-}H]^-$
- Identification (fragmentation) grade**:** A and B
- Main ion adduct used for quantification**:** $[M\text{-}H]^-$

Representation of phosphatidylglycerol (PG) identification information obtained with LipidSearch software
e.g. $[PG(36{:}3)\text{-}H]^-$

Chromatogram

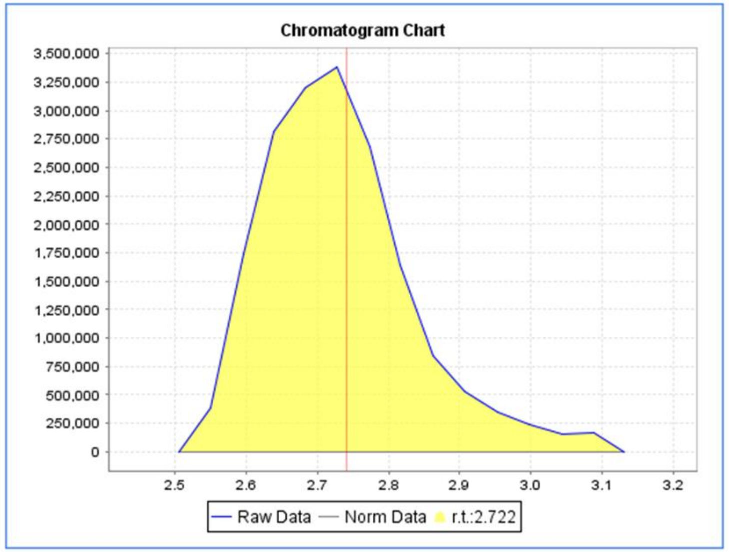

Spectrum

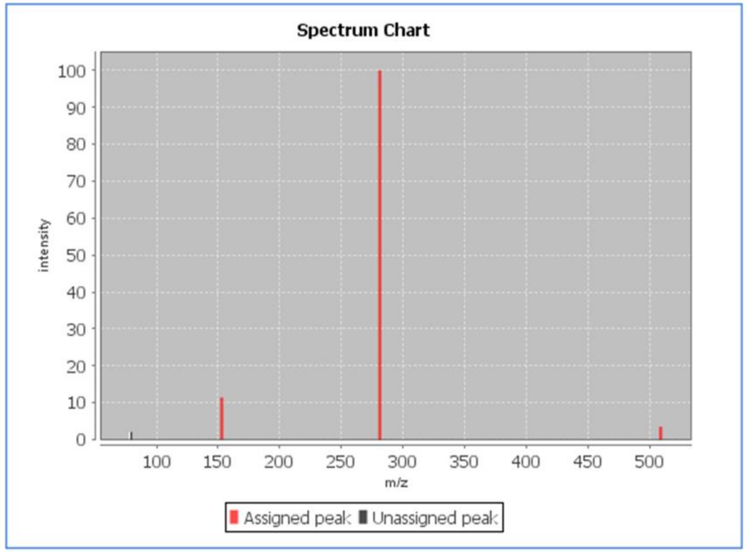

Match Lipid

LipidIon	M-Sc.	T-Sc.	Occ.	St.
LPG(18:1)-H	19.6	0.3	98.2	

Match Detail

ObsMz	Type	It.(%)	Frag.	Delta(Da)
78.9572	MS2	2.099	-	-
152.9946	MS2	11.447	GP-H3O	-0.0012
281.2489	MS2	100	FA(18:1)-H	0.0003
509.29	MS2	3.48	M-H	0.0015

Copy

Lysophosphatidylglycerol (LPG)

Information

- Ionization: Negative- and positive-ion mode
- Main ion adducts used for identification: $[M\text{-}H]^-$, $[M+NH_4]^+$ and $[M+H]^+$
- Identification (fragmentation) grade: A and B
- Main ion adduct used for quantification: $[M\text{-}H]^-$

Representation of lysophosphatidylglycerol (LPG) identification information obtained with LipidSearch software
e.g. $[LPG(18:1)\text{-}H]^-$

Chromatogram

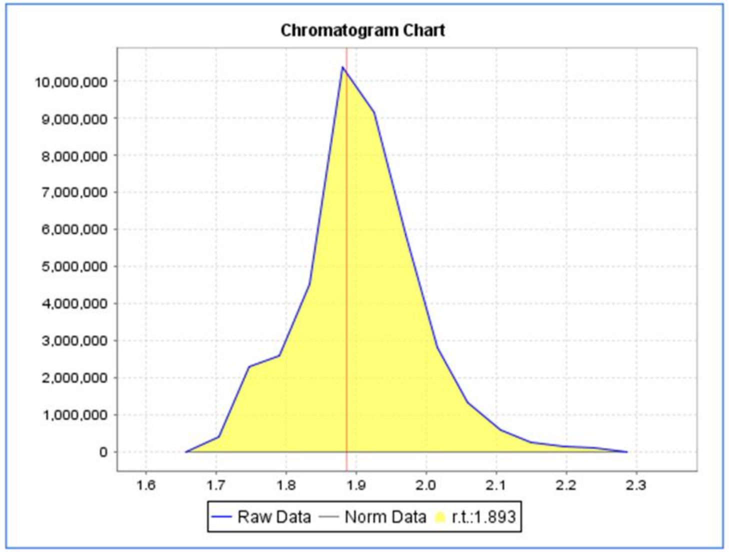

Spectrum

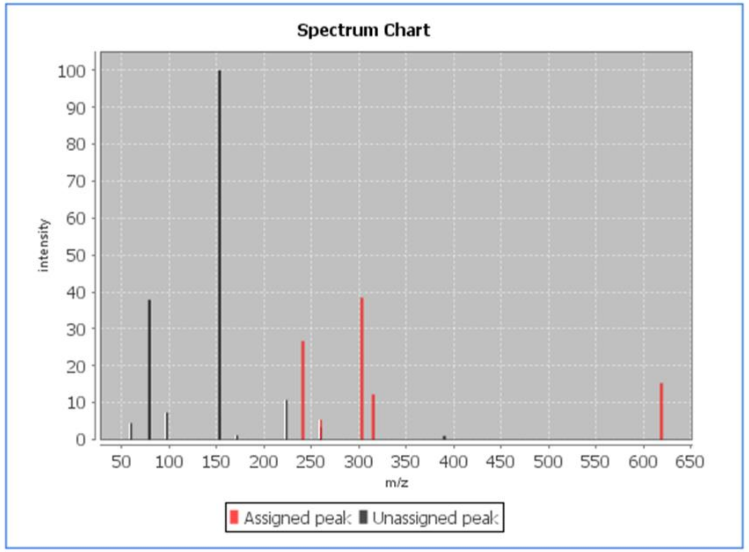

Match Lipid

LipidIon	M-Sc.	T-Sc.	Occ.	St.
LPI(20:4)-H	11.6	1	33.3	
PI(8:0e_12:4)-H	4.9	1	16.4	
PI(8:0p_12:3)-H	4.9	1	16.4	
PI(8:1e_12:3)-H	4.9	1	16.4	
PI(10:0e_10:4)-H	4.9	1	16.4	
PI(10:0p_10:3)-H	4.9	1	16.4	
PI(10:1e_10:3)-H	4.9	1	16.4	

Match Detail

ObsMz	Type	It.(%)	Frag.	Delta(Da)
59.012	MS2	4.502	-	-
78.9572	MS2	37.906	-	-
96.9678	MS2	7.343	-	-
152.9944	MS2	100	-	-
171.0054	MS2	1.182	-	-
223.0007	MS2	10.776	-	-
241.0116	MS2	26.716	PH(inositol)-H2(	-0.0003
259.0225	MS2	3.357	-	-
259.2433	MS2	5.341	FA(20:4)-H-CO:	0.0002
303.2331	MS2	38.454	FA(20:4)-H	0.0001
315.049	MS2	12.231	M-FA(20:4)-H	0.0003
390.1433	MS2	1.035	-	-
619.2891	MS2	15.299	M-H	0.0002

Copy

Lysophosphatidylinositol (LPI)

Information

- Ionization: Negative- and positive-ion mode
- Main ion adducts used for identification: $[M-H]^-$, $[M+H]^+$ and $[M+NH_4]^+$
- Identification (fragmentation) grade: A and B
- Main ion adduct used for quantification: $[M-H]^-$

Representation of lysophosphatidylinositol (LPI) identification information obtained with LipidSearch software
e.g. $[LPI(20:4)-H]^-$

Chromatogram

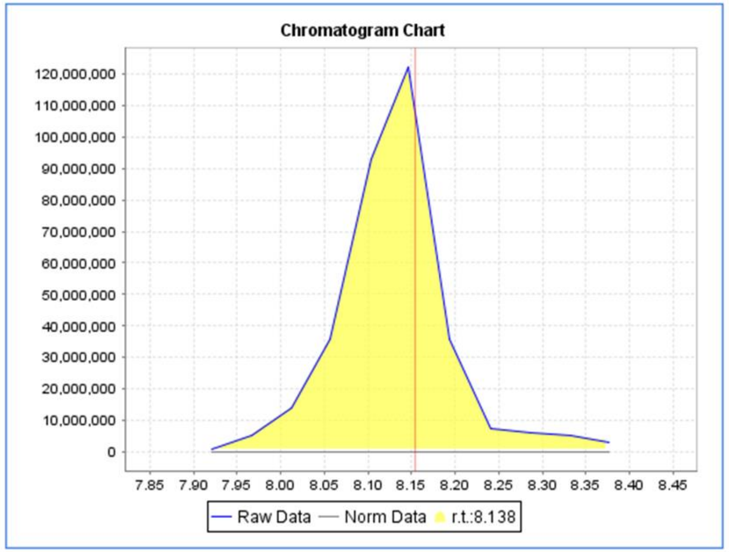

Spectrum

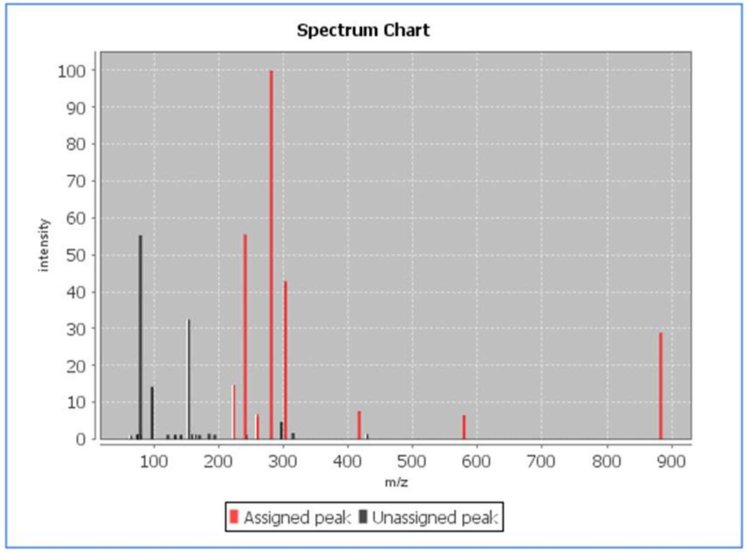

Match Lipid

LipidIon	M-Sc.	T-Sc.	Occ.	St.
◉ PI(18:1_20:4)-H	48.9	0.1	65.2	☒
○ PI(16:0_22:5)-H	6.2	0.1	20.7	☒
○ PI(16:1_22:4)-H	6.2	0.1	20.7	☒
○ PI(18:0_20:5)-H	6.2	0.1	20.7	☒
○ PI(20:3_18:2)-H	6.2	0.1	20.7	☒
○ PI(18:3_20:2)-H	6.2	0.1	20.7	☒
○ PI(18:4_20:1)-H	6.2	0.1	20.7	☒
○ PI(24:1_14:4)-H	6.2	0.1	20.7	☒
○ PI(24:2_14:3)-H	6.2	0.1	20.7	☒
○ PI(26:1_12:4)-H	6.2	0.1	20.7	☒
○ PI(27:1_11:4)-H	6.2	0.1	20.7	☒
○ PI(28:1_10:4)-H	6.2	0.1	20.7	☒

Match Detail

ObsMz	Type	It.(%)	Frag.	Delta(Da)
193.89	MS2	1.24	-	-
223.0008	MS2	14.71	PH(inositol)-2H2O-H	-0.0006
241.0116	MS2	55.524	PH(inositol)-H2O-H	-0.0003
242.1822	MS2	1.226	-	-
259.0224	MS2	5.982	IP	0
259.2432	MS2	6.788	FA(20:4)-H-CO2	0.0001
281.2487	MS2	100	FA(18:1)-H	0.0001
297.0378	MS2	4.706	-	-
303.2331	MS2	42.824	FA(20:4)-H	0.0001
315.0498	MS2	1.672	-	-
417.2404	MS2	7.655	LPA(18:1)-H3O	-0.0008
428.7987	MS2	1.391	-	-
579.2956	MS2	6.538	LPI(18:1)-H2O-H	0.0017
883.5342	MS2	28.895	M-H	0

Copy

Phosphatidylinositol (PI)

Information

- Ionization: Negative-ion mode
- Main ion adduct used for identification: $[M\text{-}H]^-$
- Identification (fragmentation) grade: A and B
- Main ion adduct used for quantification: $[M\text{-}H]^-$

Representation of phosphatidylinositol (PI) identification information obtained with LipidSearch software
e.g. $[PI(38{:}5)\text{-}H]^-$

Chromatogram

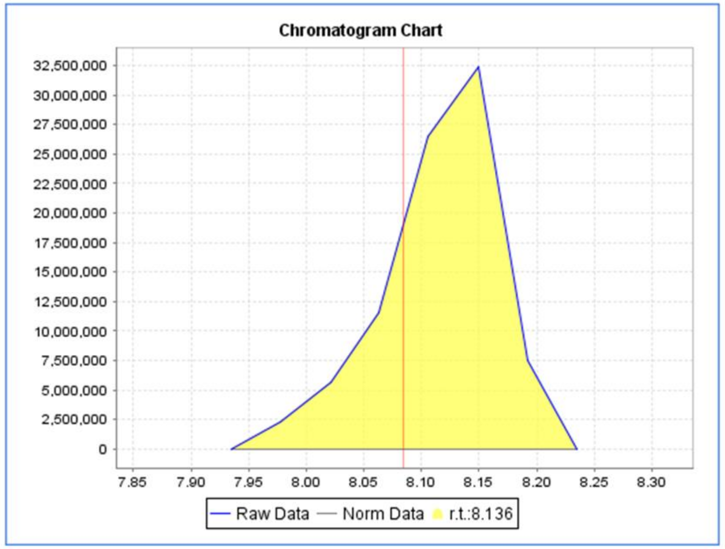

Spectrum

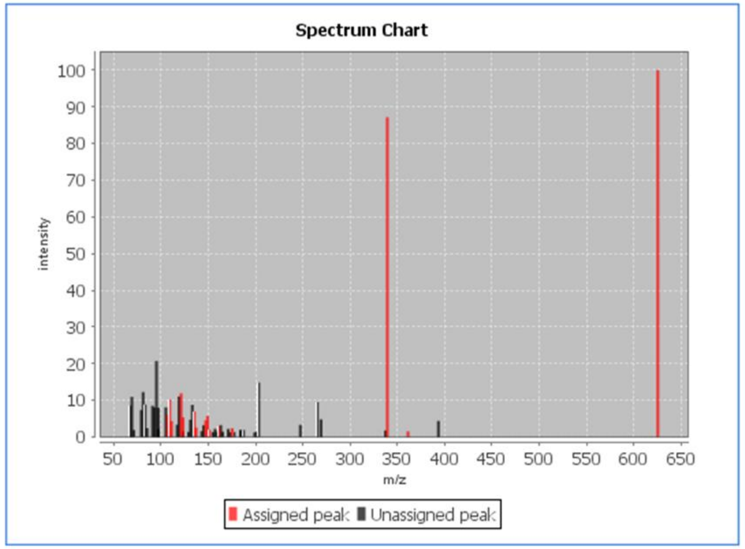

Match Lipid

LipidIon	M-Sc.	T-Sc.	Occ.	St.
**PI(18:1_20:4)+NH4**	**17.2**	**0.4**	**55.3**	
PI(20:3_18:2)+NH4	7.7	0.4	36.3	
PI(16:0_22:5)+NH4	4	0.4	35.9	
PI(16:1_22:4)+NH4	4	0.4	35.9	
PI(18:0_20:5)+NH4	4	0.4	35.9	
PI(18:3_20:2)+NH4	4	0.4	35.9	
PI(18:4_20:1)+NH4	4	0.4	35.9	
PI(24:1_14:4)+NH4	4	0.4	35.9	
PI(24:2_14:3)+NH4	4	0.4	35.9	
PI(26:1_12:4)+NH4	4	0.4	35.9	
PI(27:1_11:4)+NH4	4	0.4	35.9	
PI(28:1_10:4)+NH4	4	0.4	35.9	

Match Detail

ObsMz	Type	It.(%)	Frag.	Delta(Da)
184.0736	MS2	1.935	-	-
185.1331	MS2	1.895	-	-
187.1487	MS2	1.936	-	-
199.1486	MS2	1.269	-	-
201.1642	MS2	1.5	-	-
203.1801	MS2	14.867	-	-
247.2429	MS2	3.324	-	-
265.2535	MS2	9.52	-	-
269.2272	MS2	4.808	-	-
337.2743	MS2	1.79	-	-
339.2905	MS2	87.173	MG(18:1)-OH	0.0011
361.2748	MS2	1.54	MG(20:4)-OH	0.0011
393.3015	MS2	4.379	-	-
625.5202	MS2	100	NL[PI,+NH4]+H	0.0012

Copy

Phosphatidylinositol (PI)

Information

- Ionization: Positive-ion mode
- Main ion adducts used for identification: $[M+H]^+$, $[M+Na]^+$, $[M+K]^+$ and $[M+NH_4]^+$
- Identification (fragmentation) grade: A and B
- Main ion adduct used for quantification: $[M+NH_4]^+$

Representation of phosphatidylinositol (PI) identification information obtained with LipidSearch software e.g. $[PI(38:5)+NH_4]^+$

Lysophosphatidylserine (LPS)

Chromatogram

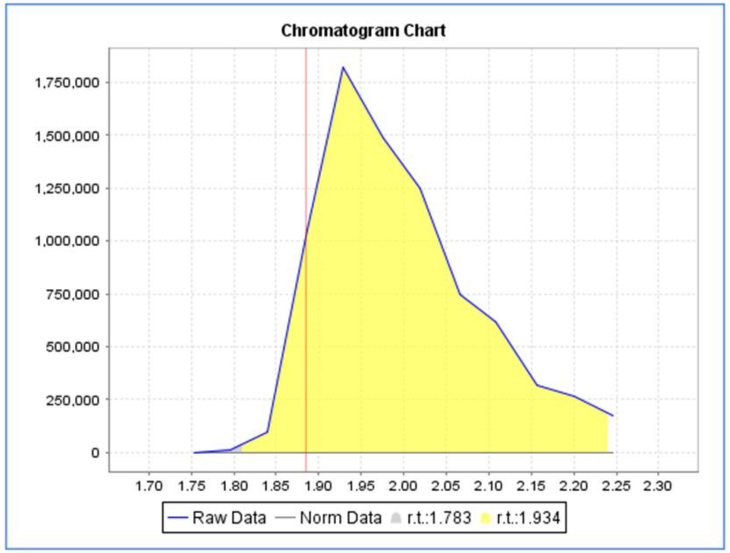

Spectrum

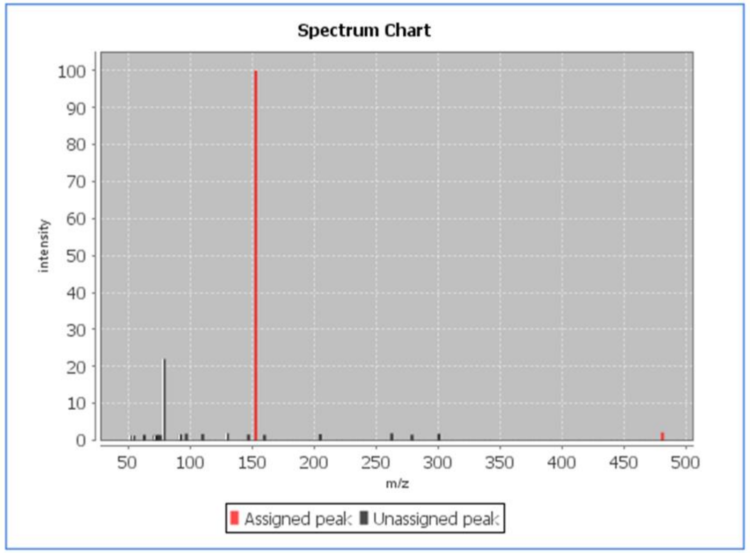

Match Lipid

LipidIon	M-Sc.	T-Sc.	Occ.	St.
LPS(22:6)-H	13.4	0.8	66.9	

Match Detail

ObsMz	Type	It.(%)	Frag.	Delta(Da)
78.9573	MS2	22.01	-	-
91.8837	MS2	1.591	-	-
92.1582	MS2	1.629	-	-
96.9682	MS2	1.832	-	-
110.261	MS2	1.719	-	-
130.0197	MS2	1.919	-	-
147.2038	MS2	1.599	-	-
152.9947	MS2	100	GP-H3O	-0.0011
160.1281	MS2	1.488	-	-
205.06	MS2	1.672	-	-
262.7444	MS2	1.934	-	-
279.1598	MS2	1.549	-	-
300.7773	MS2	1.826	-	-
481.2378	MS2	2.152	M-Serine+H2O-H	0.0017

Copy

Information

- Ionization: Negative- and positive-ion mode
- Main ion adducts used for identification: $[M\text{-}H]^-$, $[M+H]^+$ and $[M+NH_4]^+$
- Identification (fragmentation) grade: A and B
- Main ion adduct used for quantification: $[M\text{-}H]^-$

Representation of lysophosphatidylserine (LPS) identification information obtained with LipidSearch software
e.g. $[LPS(22:6)\text{-}H]^-$

Chromatogram

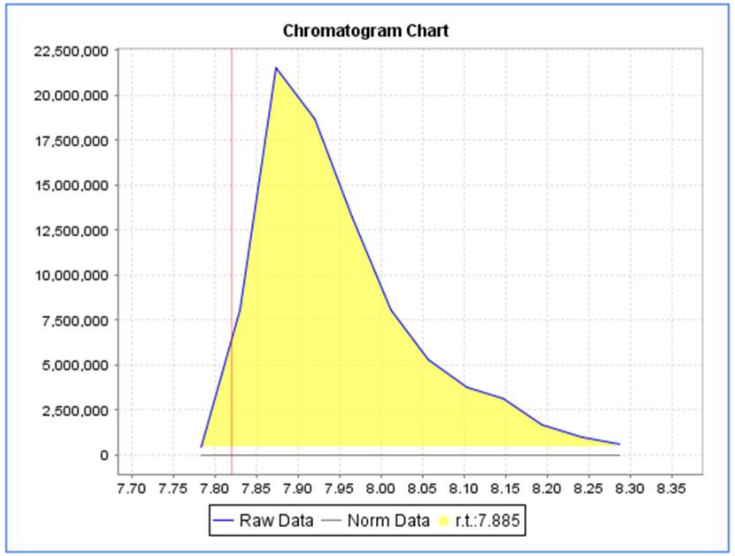

Spectrum

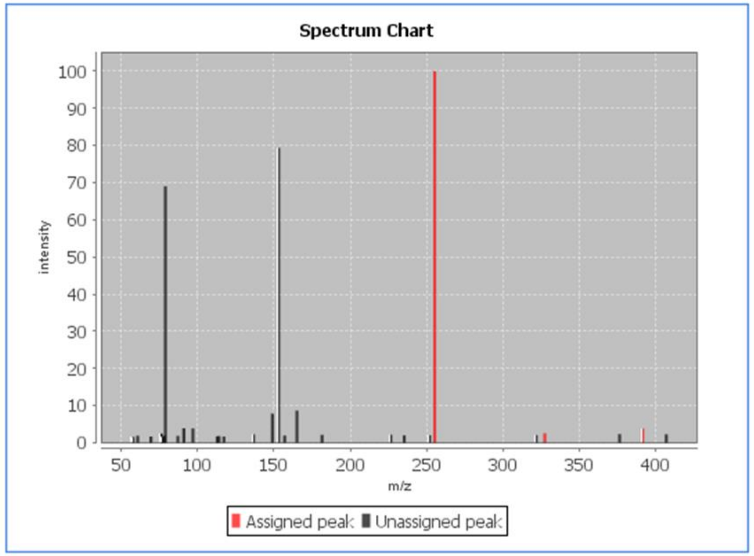

Phosphatidylserine (PS)

Information

- Ionization**:** Negative-ion mode
- Main ion adduct used for identification**:** $[M\text{-}H]^-$
- Identification (fragmentation) grade**:** A and B
- Main ion adduct used for quantification**:** $[M\text{-}H]^-$

Match Lipid

LipidIon	M-Sc.	T-Sc.	Occ.	St.
PS(16:0_22:6)-H	10	0.6	33.2	

Match Detail

ObsMz	Type	It.(%)	Frag.	Delta(Da)
149.0084	MS2	7.884	-	-
152.9945	MS2	79.355	-	-
156.9806	MS2	1.946	-	-
165.0397	MS2	8.666	-	-
181.5265	MS2	2.147	-	-
226.4443	MS2	2.178	-	-
235.3255	MS2	2.008	-	-
251.8229	MS2	2.036	-	-
255.233	MS2	100	FA(16:0)-H	0.0001
321.593	MS2	2.095	-	-
327.2328	MS2	2.593	FA(22:6)-H	-0.0001
376.2403	MS2	2.324	-	-
391.2271	MS2	3.867	LPS(16:0)-Serine-H	0.0016
406.8138	MS2	2.288	-	-

Copy

Representation of phosphatidylserine (PS) identification information obtained with LipidSearch software e.g. $[PS(38:6)\text{-}H]^-$

Chromatogram

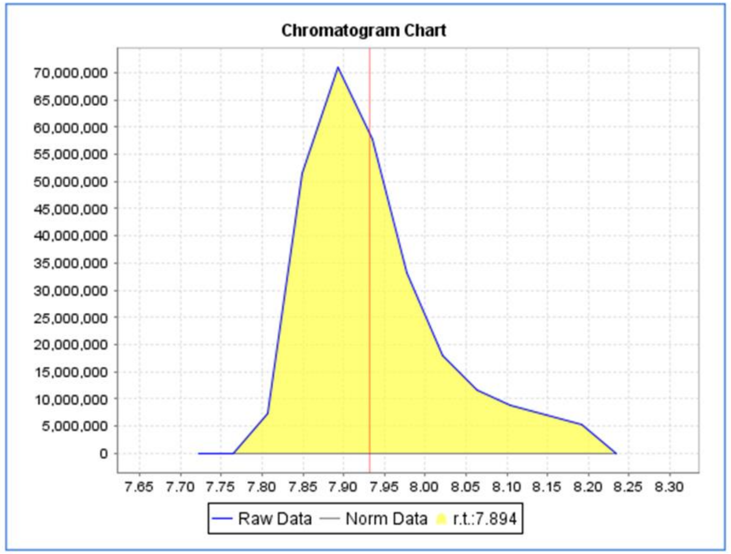

Spectrum

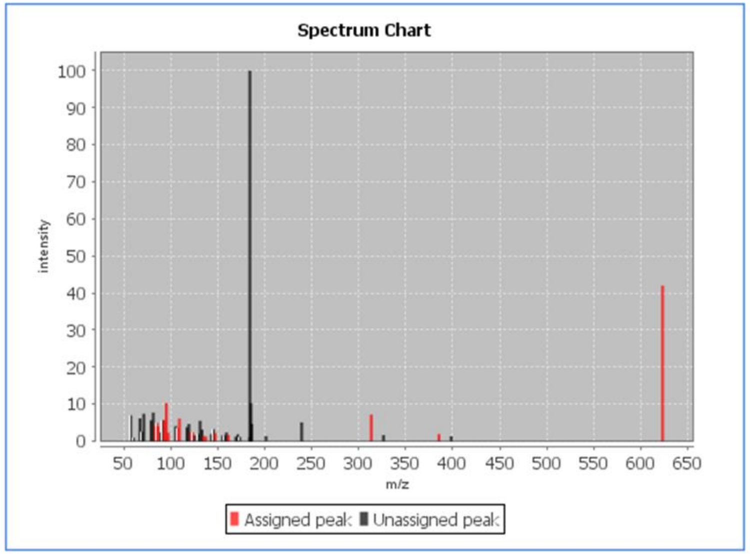

Match Lipid

	LipidIon	M-Sc.	T-Sc.	Occ.	St.
◉	**PS(16:0_22:6)+H**	**10.3**	**0.7**	**33**	⊠
○	PS(16:0_20:3)+Na	6.9	5.6	32.4	⊠
○	PS(16:1_22:5)+H	3.4	0.7	30.1	⊠
○	PS(18:1_20:5)+H	3.4	0.7	30.1	⊠
○	PS(18:2_20:4)+H	3.4	0.7	30.1	⊠
○	PS(18:3_20:3)+H	3.4	0.7	30.1	⊠
○	PS(18:4_20:2)+H	3.4	0.7	30.1	⊠
○	PS(14:0_22:3)+Na	3.4	5.6	30.1	⊠
○	PS(16:1_20:2)+Na	3.4	5.6	30.1	⊠
○	PS(18:0_18:3)+Na	3.4	5.6	30.1	⊠
○	PS(18:1_18:2)+Na	3.4	5.6	30.1	⊠

Match Detail

ObsMz	Type	It.(%)	Frag.	Delta(Da)
161.1326	MS2	1.694	C12H17	0.0002
169.1013	MS2	1.215	-	-
171.1168	MS2	1.812	-	-
173.1325	MS2	1.193	-	-
184.0052	MS2	1.106	-	-
184.0734	MS2	100	-	-
185.0766	MS2	10.281	-	-
186.0777	MS2	4.657	-	-
201.1637	MS2	1.341	-	-
239.237	MS2	5.072	-	-
313.274	MS2	7.193	MG(16:0)-OH	0.0003
326.269	MS2	1.652	-	-
385.2733	MS2	1.941	MG(22:6)-OH	-0.0004
398.2693	MS2	1.333	-	-
623.5031	MS2	41.97	NL[PS]	-0.0003

Copy

Phosphatidylserine (PS)

<u>Information</u>

- Ionization**:** Positive-ion mode
- Main ion adducts used for identification**:** $[M+H]^+$, $[M+NH_4]^+$ and $[M+Na]^+$
- Identification (fragmentation) grade**:** A and B
- Main ion adducts used for quantification**:** $[M+H]^+$ and $[M+NH_4]^+$

Representation of phosphatidylserine (PS) identification information obtained with LipidSearch software
e.g. $[PS(38:6)+H]^+$

ObsMz	Type	It.(%)	Frag.	Delta(Da)
57.0708	MS2	7.011		
60.0817	MS2	1.011		
67.055	MS2	6.142		
69.0707	MS2	2.589		
71.0863	MS2	7.392		
79.0549	MS2	5.635		
81.0706	MS2	7.758		
83.0862	MS2	4.109	C6H11	0.0006
85.1018	MS2	4.962	C6H13	0.0007
86.097	MS2	4.023	C6H13 [isoto	0.9959
88.0398	MS2	2.347		
91.0548	MS2	5.657		
93.0703	MS2	5.669	C7H9	0.0005
95.0861	MS2	10.301	C7H11	0.0005
97.1017	MS2	2.291	C7H13	0.0005
105.0703	MS2	4.069		
107.0859	MS2	3.644	C8H11	0.0004
109.1015	MS2	6.104	C8H13	0.0004
117.0701	MS2	3.778		
119.0858	MS2	4.671		
121.1015	MS2	2.482	C9H13	0.0003
123.1171	MS2	2.414	C9H15	0.0002
125.0001	MS2	1.595		
129.07	MS2	1.934		
131.0857	MS2	5.502		
133.1013	MS2	3.127		
135.1168	MS2	1.44	C10H15	0
137.1326	MS2	1.224	C10H17	0.0001
143.0856	MS2	1.908		
145.1012	MS2	3.285		
147.1169	MS2	2.214	C11H15	0
155.0104	MS2	1.53		
157.1013	MS2	1.612		
159.1168	MS2	2.321		

Chromatogram

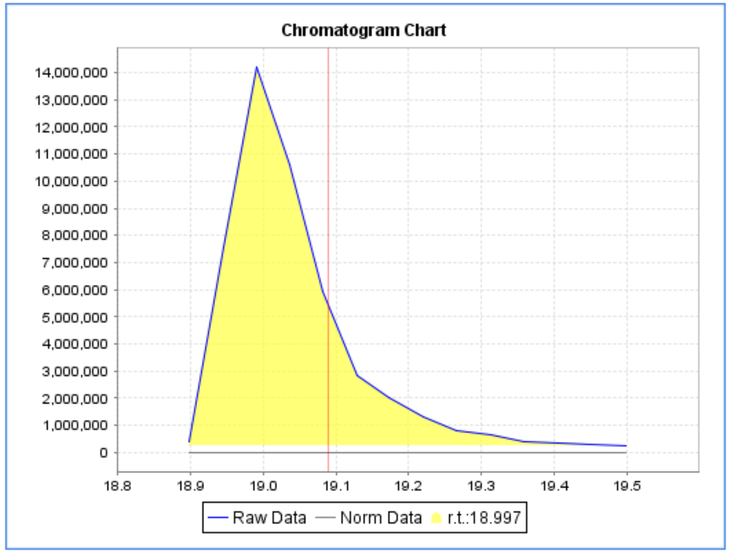

Spectrum

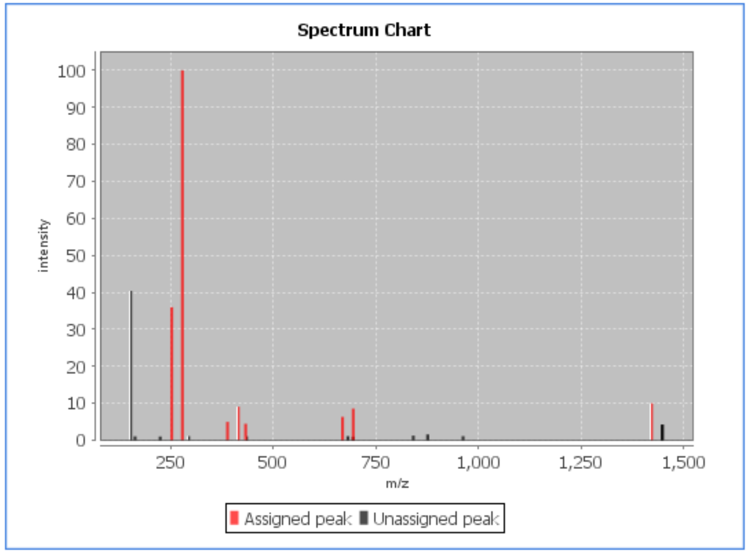

Cardiolipin (CL)

Information

- ➢ Ionization: Negative- and positive-ion mode
- ➢ Main ion adducts used for identification: $[M-H]^-$, $[M-2H]^-$ and $[M+H]^+$
- ➢ Identification (fragmentation) grade: A and B
- ➢ Main ion adduct used for quantification: $[M-H]^-$

Match Lipid

LipidIon	M-Sc.	T-Sc.	Occ.	St.
**CL(18:2_18:2_18:2_16:1)-H**	**103.7**	**0.6**	**74.1**	
CL(18:2_16:1_18:2_18:2)-H	96.3	0.6	74.1	
CL(18:2_16:1_16:1_20:3)-H	66.7	0.6	74.1	
CL(12:3_24:1_18:2_16:1)-H	59.3	0.6	74.1	
CL(22:4_14:0_18:2_16:1)-H	59.3	0.6	74.1	
CL(20:4_16:0_18:2_16:1)-H	59.3	0.6	74.1	
CL(18:3_18:1_18:2_16:1)-H	59.3	0.6	74.1	
CL(18:4_18:0_18:2_16:1)-H	59.3	0.6	74.1	
CL(22:3_14:1_18:2_16:1)-H	59.3	0.6	74.1	

Match Detail

ObsMz	Type	It.(%)	Frag.	Delta(Da)
253.2172	MS2	35.984	FA(16:1)-H	-0.0001
279.2329	MS2	100	FA(18:2)-H	-0.0001
294.3091	MS2	1.134	-	-
389.2102	MS2	4.998	LPA(16:1)-H3O	0.0003
415.2253	MS2	9.087	LPA(18:2)-H3O	-0.0003
433.236	MS2	4.458	LPA(18:2)-H	-0.0001
435.4289	MS2	1.093	-	-
669.4474	MS2	6.323	PA(18:2/16:1)-H	-0.0027
682.466	MS2	1.132	-	-
695.1216	MS2	1.011	-	-
695.4684	MS2	8.591	PA(18:2/18:2)-H	0.0026
842.0308	MS2	1.259	-	-
876.8726	MS2	1.58	-	-
963.2915	MS2	1.119	-	-
1421.9425	MS2	10.015	M-H	-0.0068

Copy

Representation of cardiolipin (CL) identification information obtained with LipidSearch software e.g. $[CL(70:7)-H]^-$

Hexosylceramide (Hex1Cer)

Chromatogram

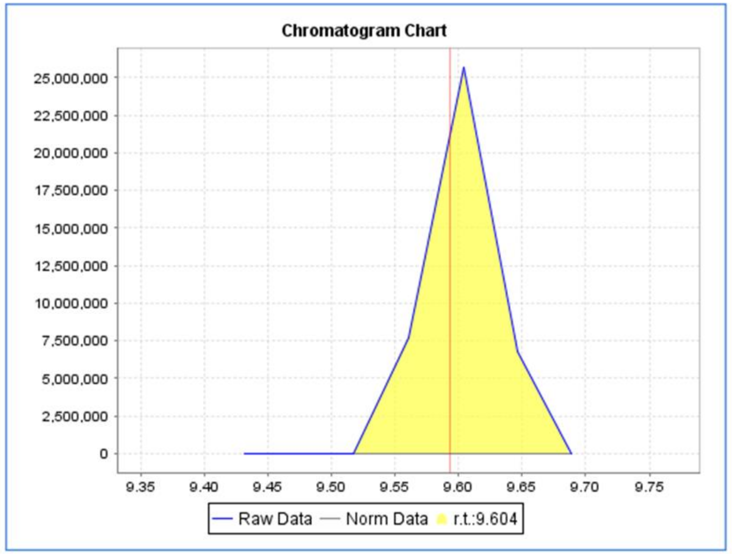

Spectrum

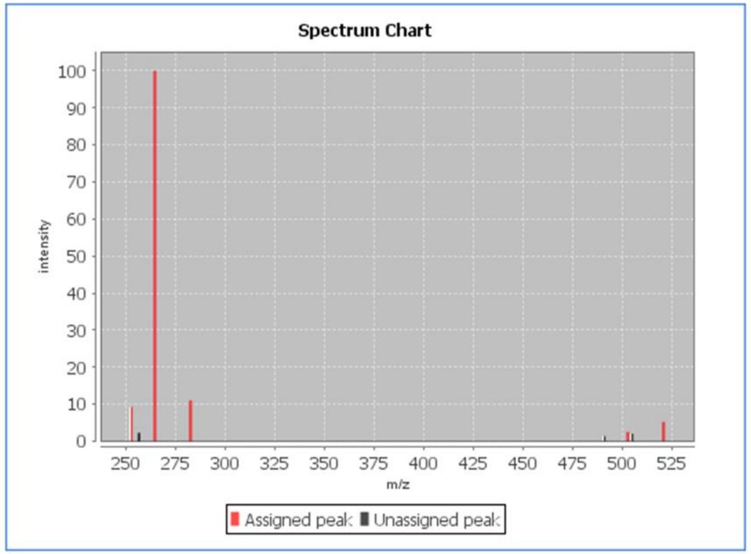

Match Lipid

LipidIon	M-Sc.	T-Sc.	Occ.	St.
◉ **Hex1Cer(d18:1_16:0)+H**	**47.9**	**0.3**	**95.7**	⊠
○ Hex1Cer(t18:0_16:0)+H-H2O	37.5	3.6	93.8	⊠
○ Hex1Cer(d17:0_17:1)+H	3.8	0.3	12.7	⊠
○ Hex1Cer(m17:1_17:0+O)+H	3.8	0.3	12.7	⊠
○ Hex1Cer(d16:0_18:1)+H	2.3	0.3	7.5	⊠
○ Hex1Cer(d17:0_17:0+O) +H-H2O	2.2	3.6	10.9	⊠

Match Detail

ObsMz	Type	It.(%)	Frag.	Delta(Da)
252.2693	MS2	9.295	SPH(d18:1)-H2O-CH2O	0.0008
256.2642	MS2	2.283	-	-
264.2694	MS2	100	SPH(d18:1)-2H2O	0.0008
282.28	MS2	11.052	SPH(d18:1)-H2O	0.0008
490.4992	MS2	1.387	-	-
502.4993	MS2	2.513	NL[G1,H2O]	0.001
504.515	MS2	2.081	-	-
520.5099	MS2	5.247	NL[G1]	0.0011

Copy

Information

- Ionization: Positive- and negative-ion mode
- Main ion adducts used for identification: $[M+H]^+$, $[M+H-H_2O]^+$, $[M+H-2H_2O]^+$ and $[M+HCOO]^-$
- Identification (fragmentation) grade: A , B and C
- Main ion adduct used for quantification: $[M+H]^+$ and $[M+HCOO]^-$

Representation of hexosylceramide (Hex1Cer) identification information obtained with LipidSearch software

e.g. $[Hex1Cer(d34:1)+H]^+$

Chromatogram

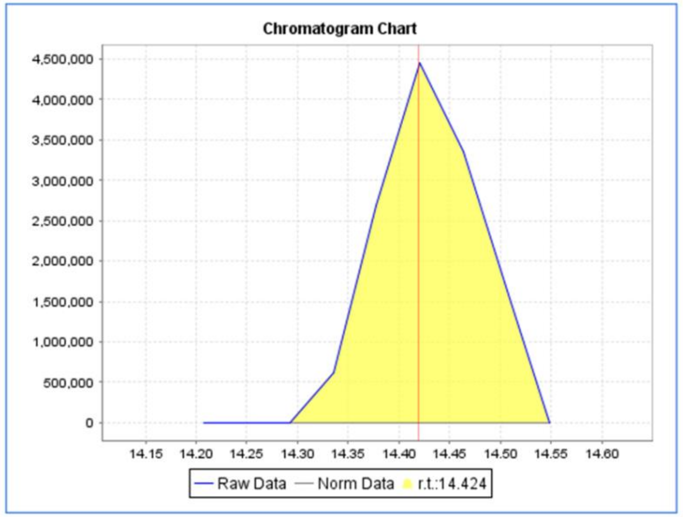

Spectrum

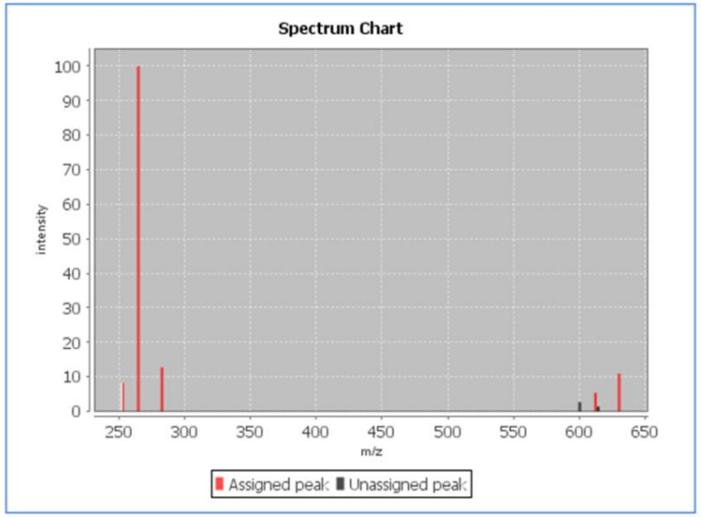

Match Lipid

LipidIon	M-Sc.	T-Sc.	Occ.	St.
**Hex2Cer(d18:1_24:1)+H**	**48.6**	**0.1**	**97.1**	
Hex2Cer(t18:0_24:1)+H-H2O	37.3	1.1	93.3	
Hex2Cer(d19:2_23:0)+H	24.7	0.1	82.3	
Hex2Cer(d18:1_24:0+O)+H-H2O	15.7	1.1	78.5	
Hex2Cer(t18:0_24:0+O)+H-2H2O	7.1	3.1	70.7	
Hex2Cer(d17:0_25:2)+H	5.2	0.1	17.4	
Hex2Cer(d16:0_26:2)+H	2.3	0.1	11.6	
Hex2Cer(d16:1_26:1)+H	2.3	0.1	11.6	
Hex2Cer(d16:2_26:0)+H	2.3	0.1	11.6	
Hex2Cer(d17:1_25:1)+H	2.3	0.1	11.6	
Hex2Cer(d18:0_24:2)+H	2.3	0.1	11.6	
Hex2Cer(d18:2_24:0)+H	2.3	0.1	11.6	
Hex2Cer(d20:0_22:2)+H	2.3	0.1	11.6	
Hex2Cer(d20:1_22:1)+H	2.3	0.1	11.6	
Hex2Cer(d20:2_22:0)+H	2.3	0.1	11.6	

Match Detail

ObsMz	Type	It.(%)	Frag.	Delta(Da)
252.2687	MS2	8.262	SPH(d18:1)-H2O-CH2(	0.0001
264.2688	MS2	100	SPH(d18:1)-2H2O	0.0002
282.2794	MS2	12.723	SPH(d18:1)-H2O	0.0002
600.6071	MS2	2.656	-	-
612.6077	MS2	5.385	NL[G2,H2O]	-0.0001
614.625	MS2	1.407	-	-
630.6184	MS2	10.962	NL[G2]	0.0001

Copy

Hexosylceramide (Hex2Cer)

Information

- Ionization: Positive- and negative-ion mode
- Main ion adducts used for identification: $[M+H]^+$, $[M+H-H_2O]^+$, $[M+H-2H_2O]^+$ and $[M+HCOO]^-$
- Identification (fragmentation) grade: A , B and C
- Main ion adduct used for quantification: $[M+H]^+$ and $[M+HCOO]^-$

Representation of hexosylceramide (Hex2Cer) identification information obtained with LipidSearch software
e.g. $[Hex2Cer(d42:2)+H]^+$

Chromatogram

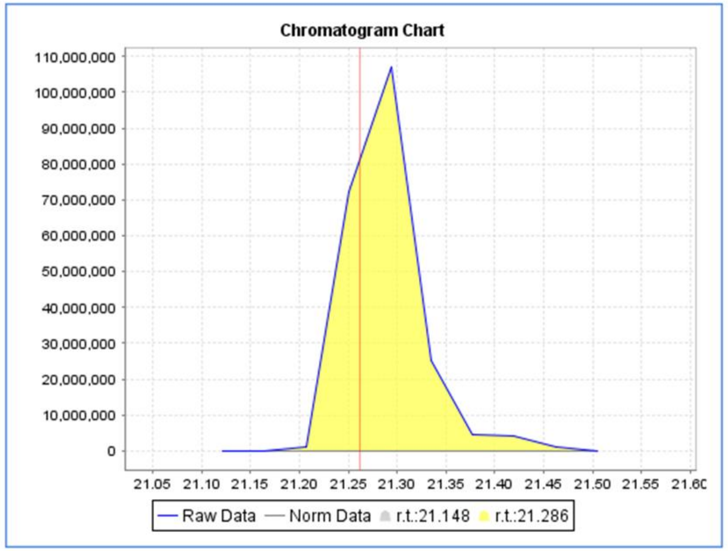

Spectrum

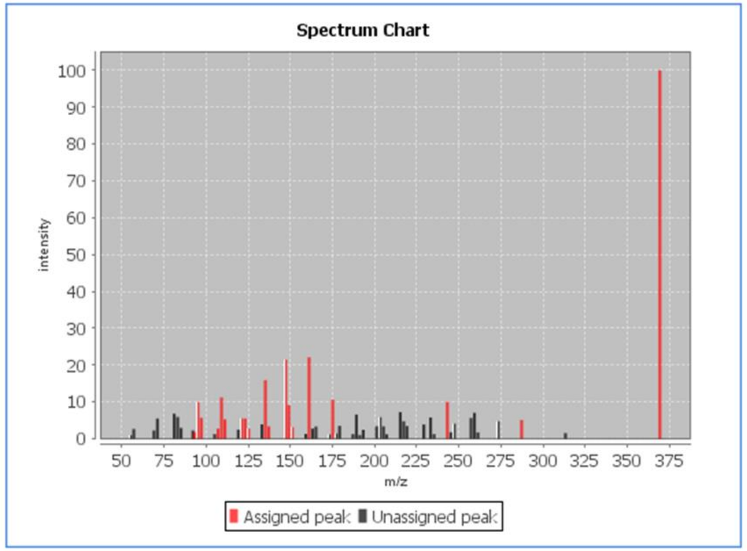

Match Lipid

LipidIon	M-Sc.	T-Sc.	Occ.	St.
ChE(20:5)+NH4	18.5	20.2	66.1	

ObsMz	Type	It.(%)	Frag.	Delta(Da)
55.0552	MS2	1.004		
57.0709	MS2	2.642		
69.0707	MS2	2.234		
71.0864	MS2	5.522		
81.0707	MS2	6.788		
83.0863	MS2	5.9		
85.102	MS2	2.932		
92.1486	MS2	2.244		
93.0706	MS2	1.757	C7H9	0.0007
95.0862	MS2	9.866	C7H11	0.0006
97.1018	MS2	5.68	C7H13	0.0007
105.0704	MS2	1.261		
107.0861	MS2	2.749	C8H11	0.0006
109.1017	MS2	11.248	C8H13	0.0005
111.1174	MS2	5.264	C8H15	0.0005
119.086	MS2	2.49		
121.1016	MS2	5.533	C9H13	0.0005
123.1172	MS2	5.516	C9H15	0.0004
125.1329	MS2	2.767	C9H17	0.0004
133.1015	MS2	3.914		
135.1171	MS2	15.943	C10H15	0.0003
137.1328	MS2	3.364	C10H17	0.0003
147.1171	MS2	21.468	C11H15	0.0003
149.1328	MS2	9.153	C11H17	0.0003
151.1485	MS2	3.143	C11H19	0.0003
159.1172	MS2	1.313		
161.1327	MS2	22.108	C12H17	0.0003
163.1484	MS2	2.78		
165.1641	MS2	3.327		
173.1328	MS2	1.165		
175.1484	MS2	10.618	C13H19	0.0003
177.1642	MS2	1.47		
179.1798	MS2	3.49		
187.1486	MS2	1.278		
189.1641	MS2	6.59		
191.1796	MS2	1.032		
193.1954	MS2	2.454		
201.1641	MS2	3.414		
203.1798	MS2	5.868		
205.1955	MS2	3.364		
207.2111	MS2	1.231		
215.1799	MS2	7.241		

Match Detail

ObsMz	Type	It.(%)	Frag.	Delta(Da)
217.1956	MS2	4.769	-	-
219.2113	MS2	3.436	-	-
229.1957	MS2	3.879	-	-
233.2269	MS2	5.77	-	-
235.2425	MS2	1.257	-	-
243.2112	MS2	10.034	C18H27	0.0005
245.2269	MS2	1.782	-	-
247.2426	MS2	4.11	-	-
257.227	MS2	5.634	-	-
259.2427	MS2	7.04	-	-
261.2585	MS2	1.757	-	-
273.2584	MS2	4.769	-	-
287.274	MS2	5.072	C21H35	0.0007
313.2901	MS2	1.542	-	-
369.3523	MS2	100	M-FA	0.0008

Copy

Cholesteryl esters and Cholesterol (ChE)

Information

- **Ionization:** Positive-ion mode
- **Main ion adducts used for identification:** $[M+NH_4]^+$, $[M+H-H_2O]^+$ and $[M+H]^+$
- **Identification (fragmentation) grade:** A , B and C
- **Main ion adducts used for quantification:**
  $[M+NH_4]^+$ in the case of cholesteryl esters
  $[M+H-H_2O]^+$ in the case of cholesterol

Representation of cholesteryl esters (ChE) identification information obtained with LipidSearch software e.g. $[ChE(20:5)+NH_4]^+$

Chromatogram

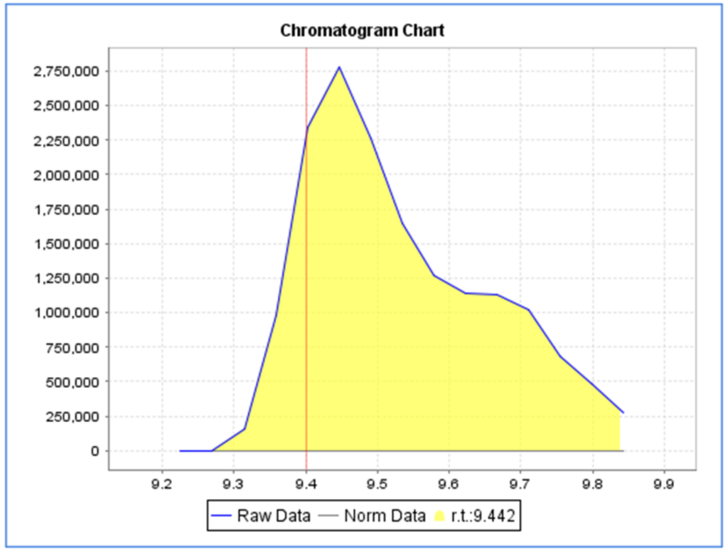

Spectrum

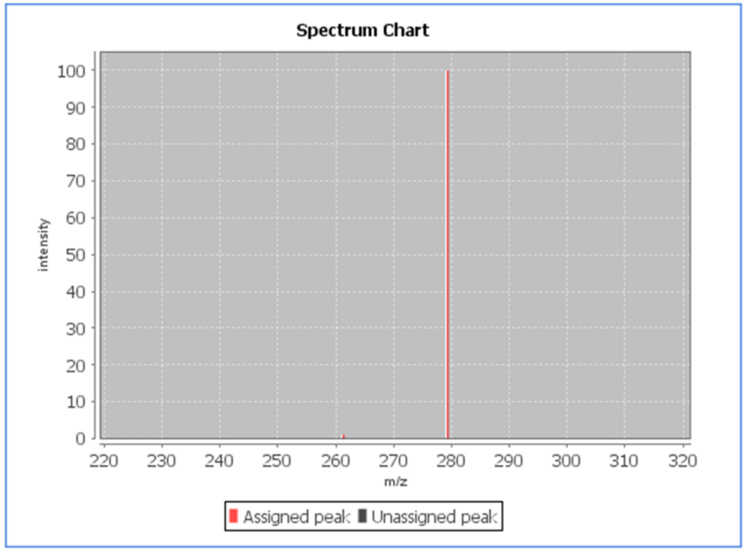

Match Lipid

LipidIon	M-Sc.	T-Sc.	Occ.	St.
FA(18:2)-H	10	6.1	100	

Match Detail

ObsMz	Type	It.(%)	Frag.	Delta(Da)
261.2226	MS2	1.046	FA(18:2)-H3O	0.0003
279.2329	MS2	100	M-H	-0.0001

Copy

Fatty acids (FA)

Information

- Ionization**:** Negative-ion mode
- Main ion adduct used for identification**:** $[M\text{-}H]^{-}$
- Identification (fragmentation) grade**:** A , B and C
- **Note**: A minimal number of free fatty acid species were identified with very low confidence (doesn't meet filtering criteria including diagnostic fragment ions, base retention time, etc.) and they were ignored for further analysis

Simple Glc series (CerG2GNAc1)

Chromatogram

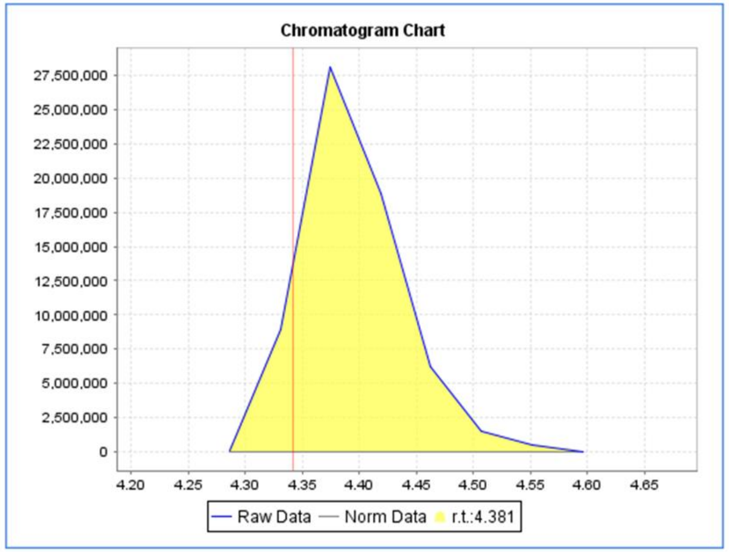

Spectrum

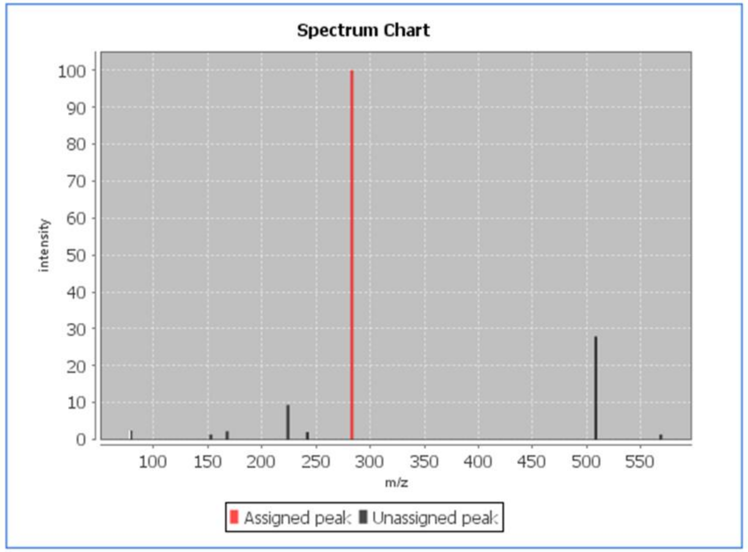

Match Lipid

LipidIon	M-Sc.	T-Sc.	Occ.	St.
CerG2GNAc1(d18:1_18:0)-H	6.8	5.3	68.1	☒

Match Detail

ObsMz	Type	It.(%)	Frag.	Delta(Da)
78.9572	MS2	2.434	-	-
152.9943	MS2	1.381	-	-
168.0418	MS2	2.267	-	-
224.0689	MS2	9.384	-	-
242.0795	MS2	1.991	-	-
283.2642	MS2	100	FA(18:0)-H	-0.0001
508.3404	MS2	27.939	-	-
568.3603	MS2	1.368	-	-

Copy

Information

- Ionization**:** Negative-ion mode
- Main ion adduct used for identification**:** $[M-H]^-$
- Identification (fragmentation) grade**:** A , B and C
- **Note**: A minimal number of CerG2GNAc1 species were identified with very low confidence (no diagnostic fragment ions) and they were ignored for further analysis

Lysodimethylphosphatidylethanolamine (LdMePE)

Chromatogram

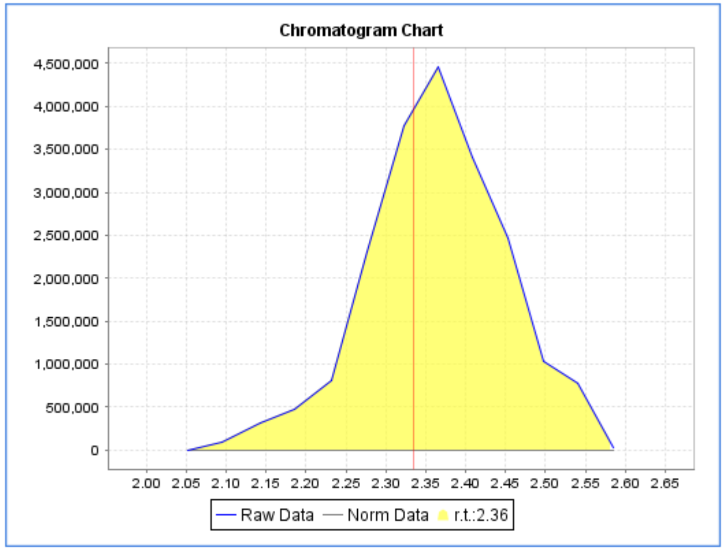

Spectrum

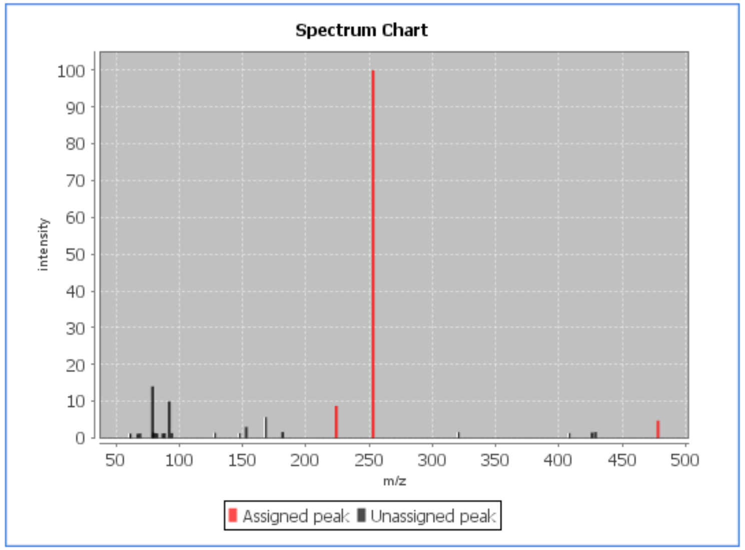

Match Lipid

	LipidIon	M-Sc.	T-Sc.	Occ.	St.
◉	**LdMePE(16:1)-H**	**13.2**	**0.1**	**66**	⊠
○	LPC(16:1)-CH3	6.1	0.1	60.6	⊠

Match Detail

ObsMz	Type	It.(%)	Frag.	Delta(Da)
88.2889	MS2	1.311	-	-
92.1381	MS2	9.991	-	-
94.2195	MS2	1.372	-	-
127.8928	MS2	1.466	-	-
147.4395	MS2	1.304	-	-
152.9945	MS2	3.023	-	-
168.0419	MS2	5.703	-	-
181.6368	MS2	1.65	-	-
224.0692	MS2	8.763	LdMeGPE-H3O	-0.0002
253.2173	MS2	100	FA(16:1)-H	0
320.3389	MS2	1.603	-	-
407.8658	MS2	1.384	-	-
426.0836	MS2	1.479	-	-
428.7619	MS2	1.662	-	-
478.2966	MS2	4.743	M-H	0.0027

Copy

Information

- Ionization**:** Negative-ion mode
- Main ion adduct used for identification**:** $[M\text{-}H]^-$
- Identification (fragmentation) grade**:** A , B and C
- **Note**: LdMePE lipid species share the same retention time and approximate equivalent abundance ratio values with identical acyl-chain length of LPC species. Possibly, in-source fragments [i.e., LPC-CH_3] and they were ignored for further analysis[5]

Chromatogram

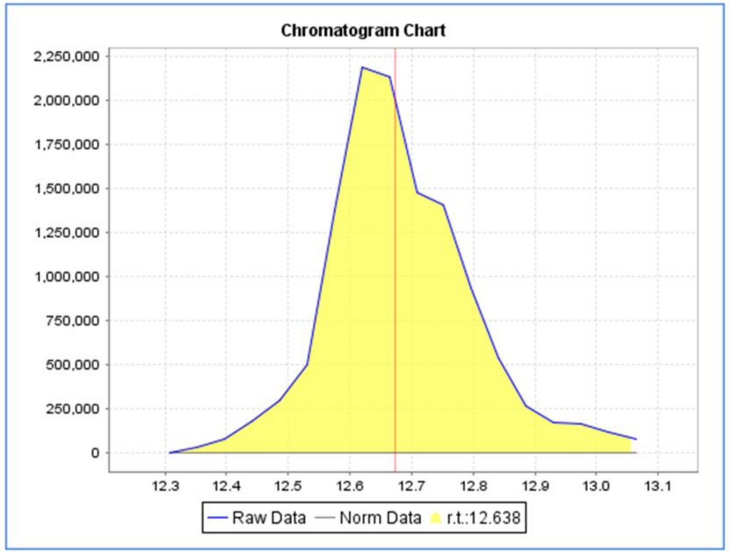

Spectrum

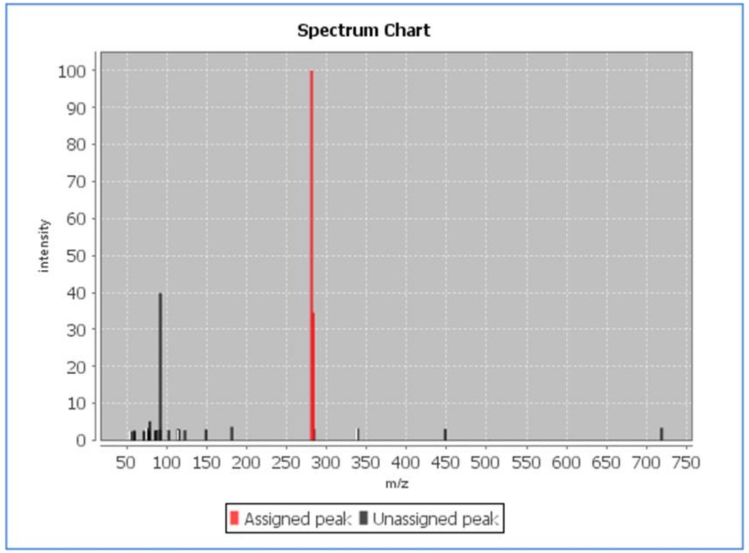

Match Lipid

LipidIon	M-Sc.	T-Sc.	Occ.	St.
dMePE(18:0_18:1)-H	11.3	0	56.6	
PC(18:0_18:1)-CH3	11.3	0.2	56.6	
PE(20:0_18:1)-H	4.2	2.3	42.1	

Match Detail

ObsMz	Type	It.(%)	Frag.	Delta(Da)
87.1767	MS2	2.799	-	-
91.7059	MS2	2.904	-	-
92.1372	MS2	39.864	-	-
102.5491	MS2	2.763	-	-
114.3476	MS2	3.185	-	-
115.1927	MS2	2.846	-	-
122.8362	MS2	2.75	-	-
149.2936	MS2	2.997	-	-
181.5578	MS2	3.7	-	-
281.2488	MS2	100	FA(18:1)-H	0.0002
283.2641	MS2	34.533	FA(18:0)-H	-0.0002
283.9593	MS2	3.062	-	-
338.8096	MS2	3.275	-	-
448.3401	MS2	3.15	-	-
719.0166	MS2	3.42	-	-

Copy

Dimethylphosphatidylethanolamine (dMePE)

Information

- Ionization**:** Negative-ion mode
- Main ion adducts used for identification**:** [M-H]$^{-}$
- Identification (fragmentation) grade**:** A , B and C
- **Note**: dMePE lipid species share the same retention time and approximate equivalent abundance ratio values with identical acyl-chain length of PC species. Possibly, in-source fragments [i.e., PC-CH_3] and they were ignored for further analysis[5]
