# Supplementary material for: Sequential lipidomic, metabolomic, and proteomic analyses of serum, liver, and heart tissue specimens from peroxisomal biogenesis factor 11α knockout mice
Source: Anal Bioanal Chem. 2022 Jan 27;414(6):2235–50. doi: 10.1007/s00216-021-03860-0 (PMC8821073; doi:10.1007/s00216-021-03860-0)
Supplement: Supplementary file 6 — Supplementary file6 (PDF 637 kb) [file 216_2021_3860_MOESM6_ESM.pdf]

Electronic Supplementary Material_6; Data file S2

Sequential lipidomic, metabolomic and proteomic analyses of serum, liver and heart tissue specimens from peroxisomal biogenesis factor 11α knockout mice

Vannuruswamy Garikapati[1,2], Claudia Colasante[2], Eveline Baumgart-Vogt[2*], Bernhard Spengler[1*]

[1]Institute of Inorganic and Analytical Chemistry, Justus Liebig University Giessen, 35392 Giessen, Germany

[2]Institute for Anatomy and Cell Biology II, Division of Medical Cell Biology, Justus Liebig University Giessen, 35392 Giessen, Germany

Proteomics raw data quality evaluation using RawMeat (v2.1) software

Wild type control and peroxisomal biogenesis factor 11α knockout mouse liver proteome

Base peak intensity from full MS scans as a function of retention time

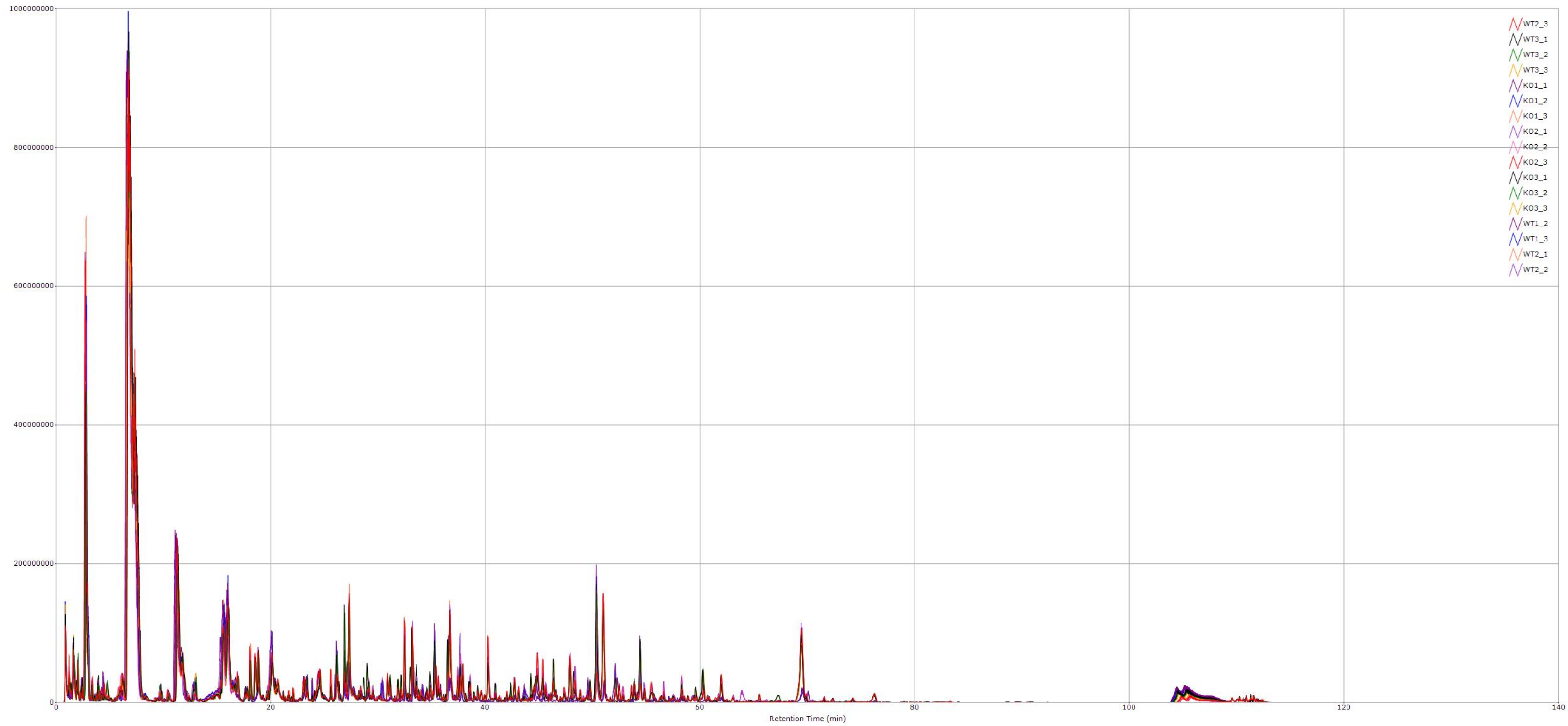

Horizontal axis (X-axis): Retention time in minutes; Vertical axis (Y-axis): Base peak intensity per full MS scans

Number of MS2 scans in between consecutive scans

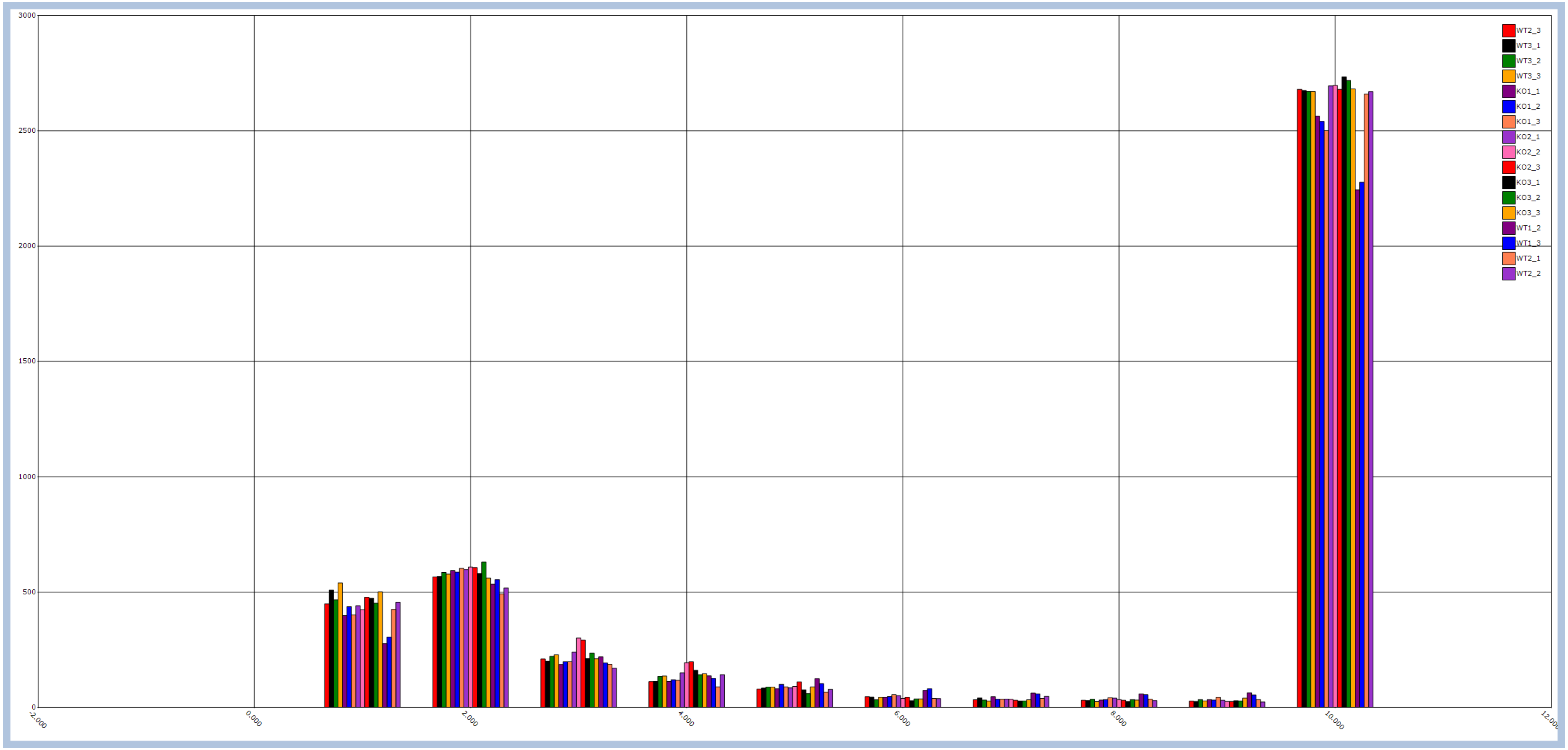

Horizontal axis (X-axis): Number of MS2 scans in between consecutive scans; Vertical axis (Y-axis): Frequency

MS2 charge distribution

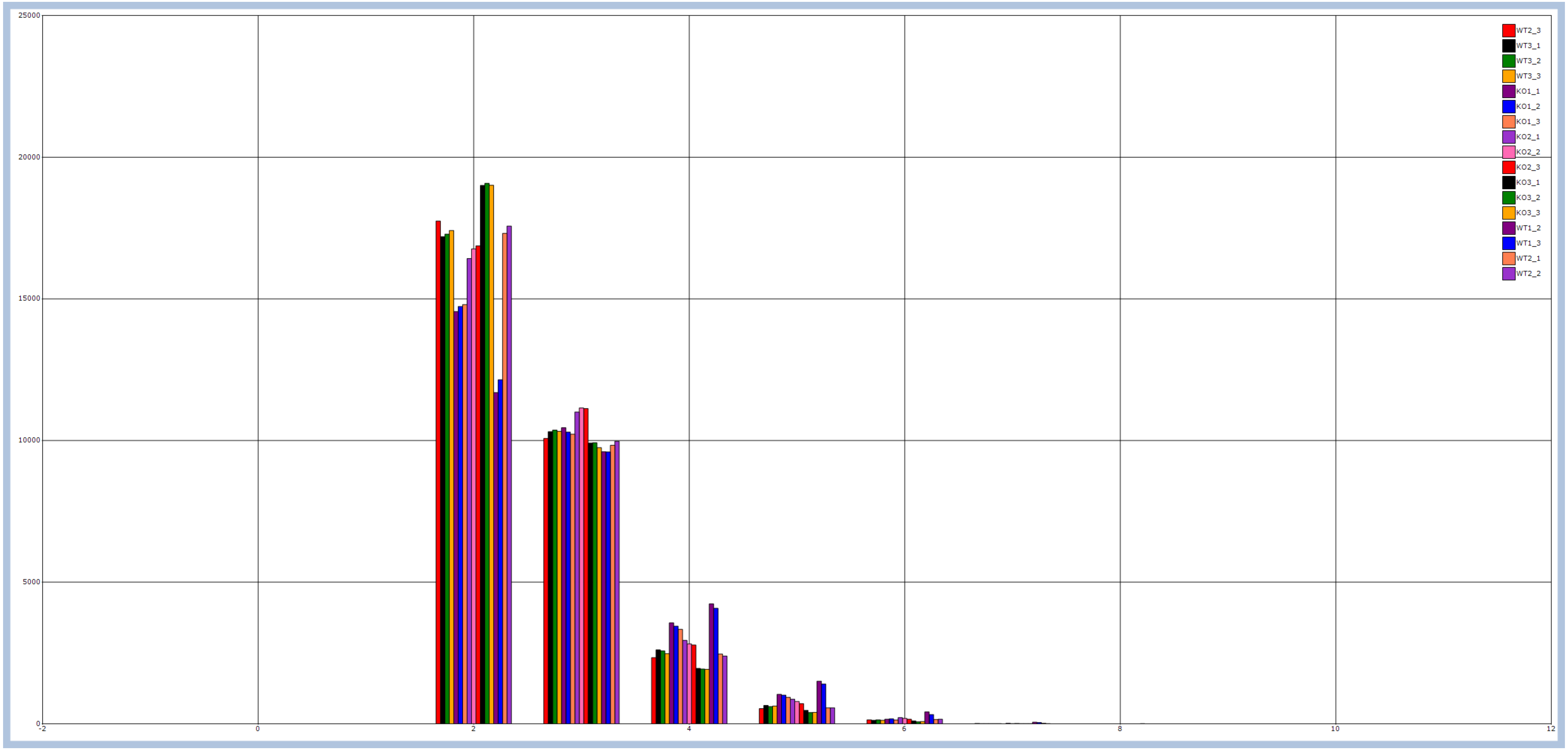

Horizontal axis (X-axis): Precursor ion charge; Vertical axis (Y-axis): Counts per charge

Proteomics raw data quality evaluation using RawMeat (v2.1) software

Wild type control and peroxisomal biogenesis factor 11α knockout mouse heart proteome

Base peak intensity from full MS scans as a function of retention time

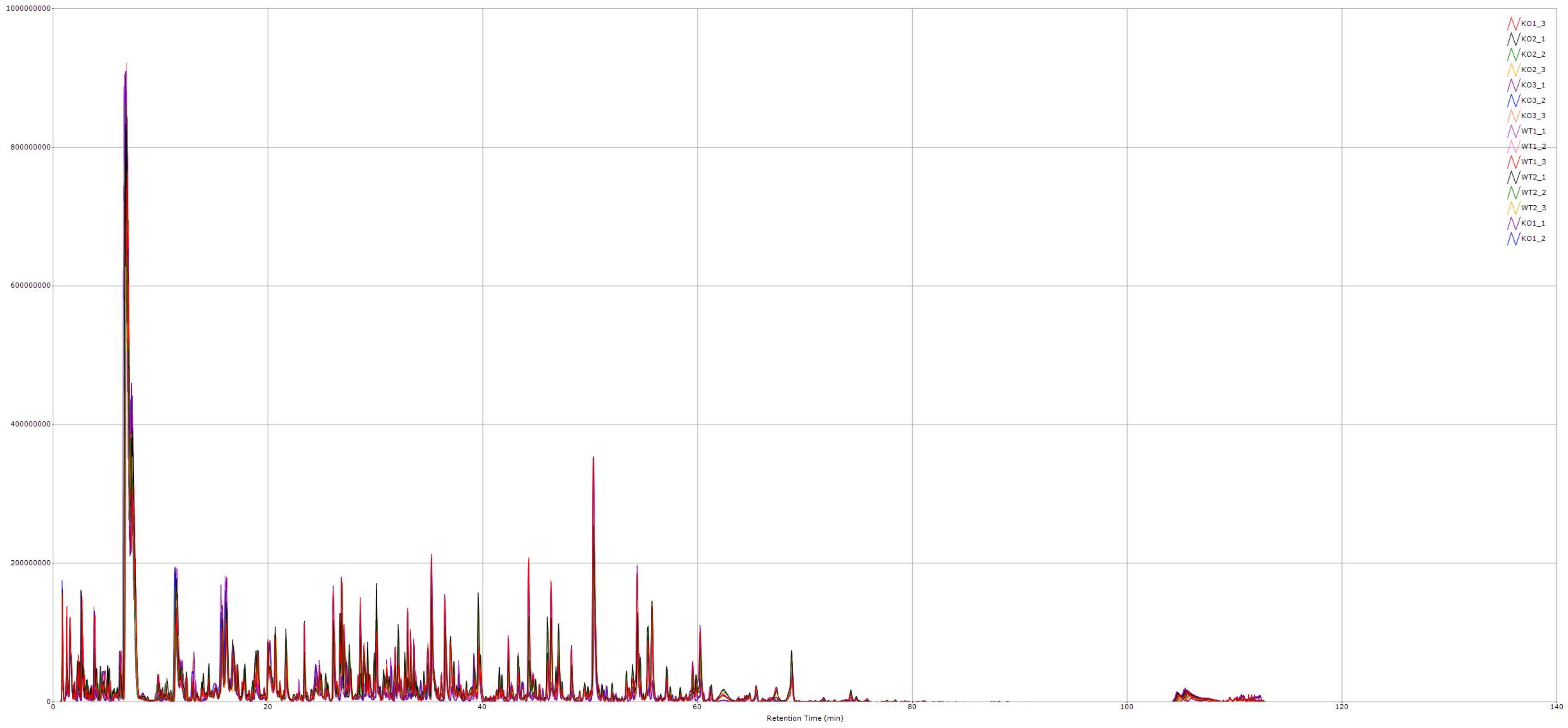

Horizontal axis (X-axis): Retention time in minutes; Vertical axis (Y-axis): Base peak intensity per full MS scans

Number of MS2 scans in between consecutive scans

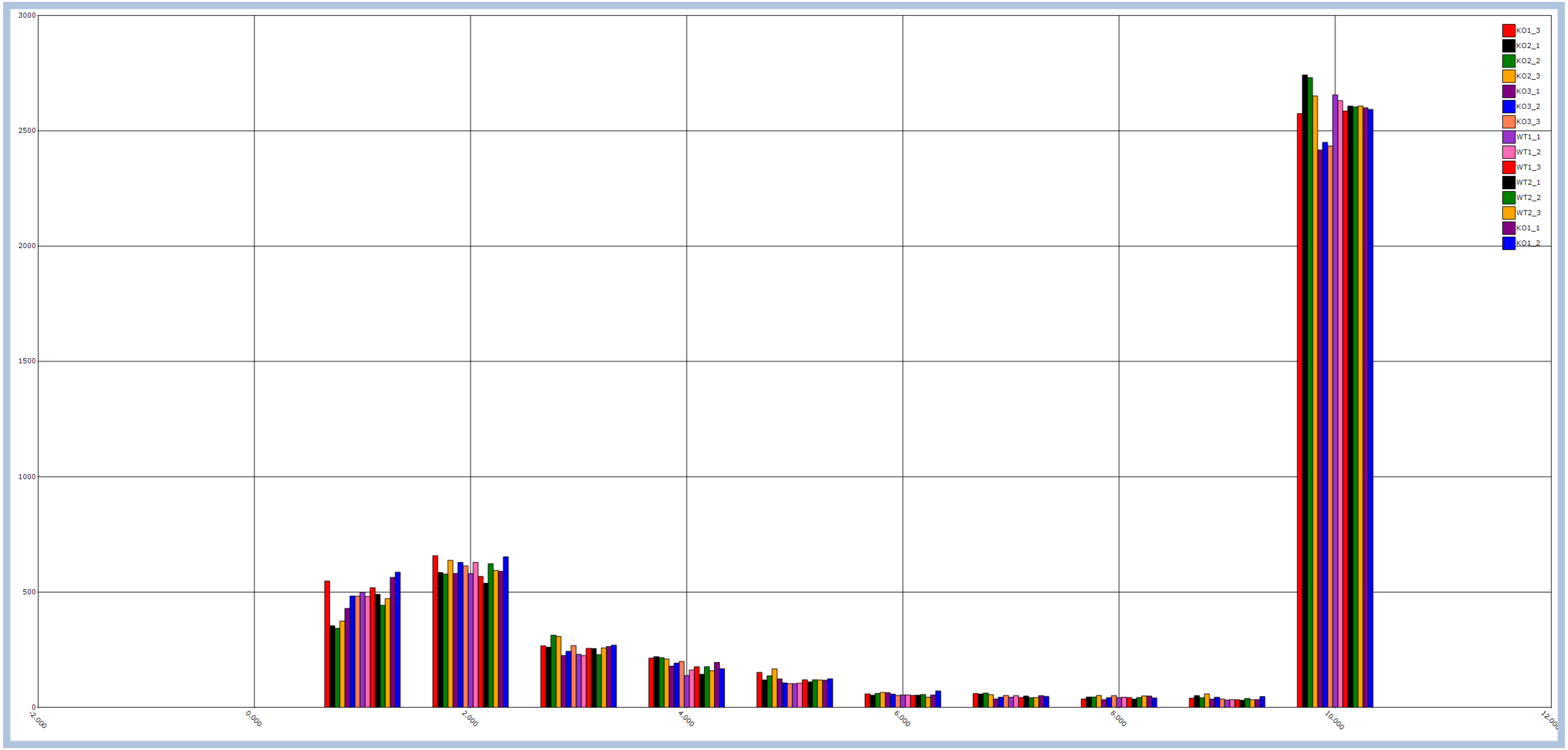

Horizontal axis (X-axis): Number of MS2 scans in between consecutive scans; Vertical axis (Y-axis): Frequency

MS2 charge distribution

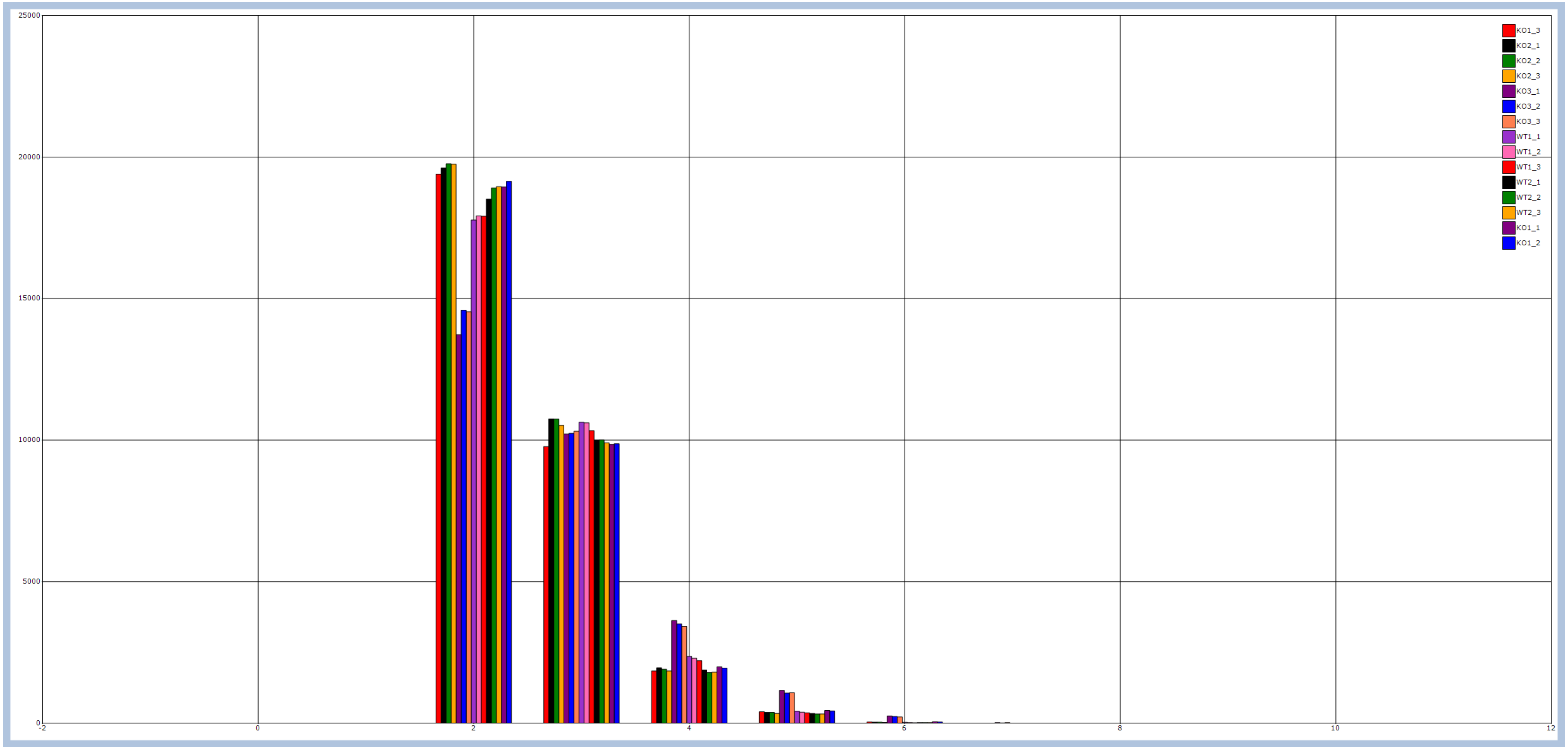

Horizontal axis (X-axis): Precursor ion charge; Vertical axis (Y-axis): Counts per charge

Principal component analysis

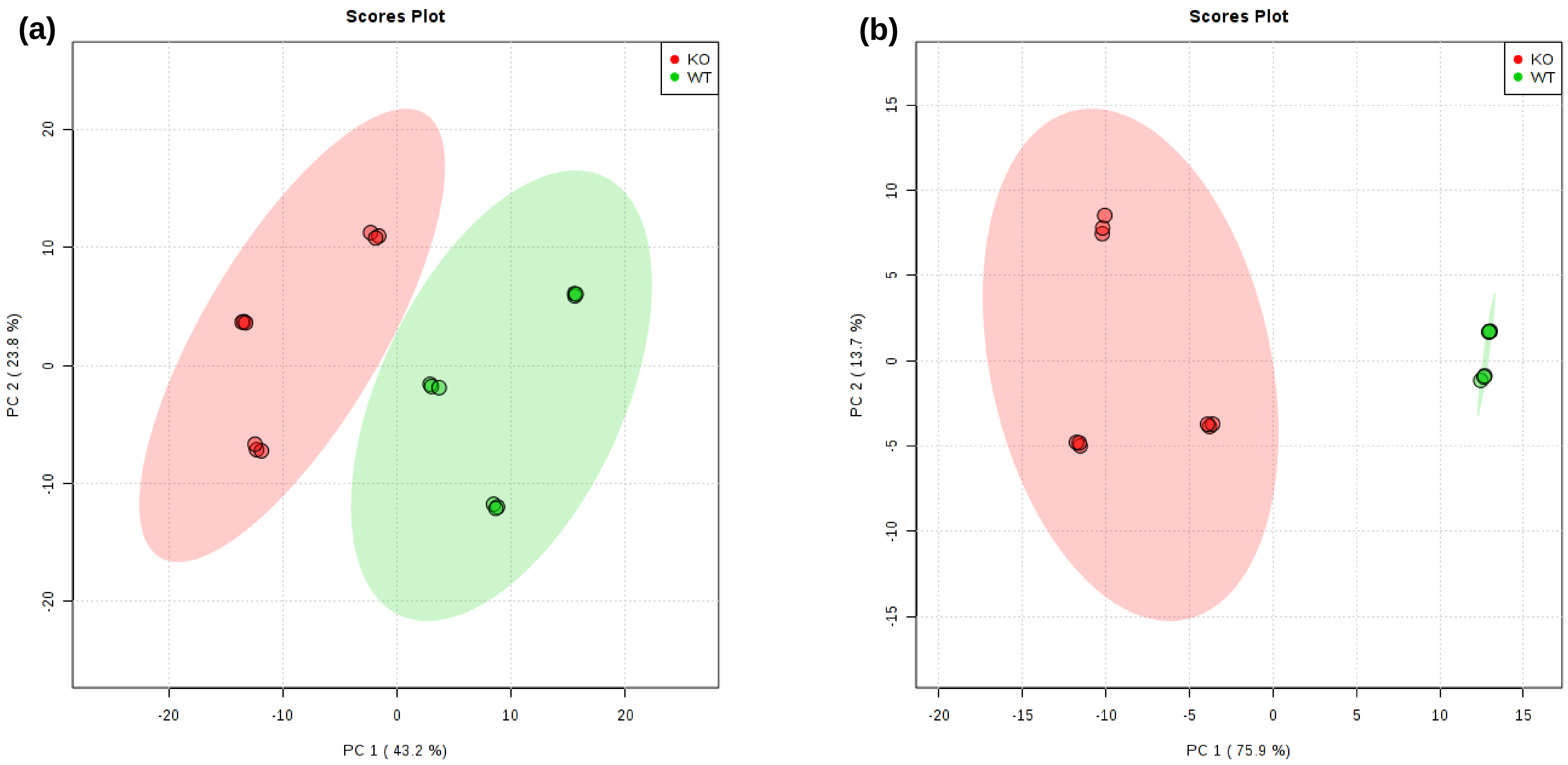

Principal component analysis of proteomics data obtained from wild type control (WT; $Pex11\alpha^{+/+}$) and peroxisomal biogenesis factor 11α knockout (KO; $Pex11\alpha^{-/-}$) mouse a) liver and b) heart tissue homogenates
