# Supplementary material for: Sequential lipidomic, metabolomic, and proteomic analyses of serum, liver, and heart tissue specimens from peroxisomal biogenesis factor 11α knockout mice
Source: Anal Bioanal Chem. 2022 Jan 27;414(6):2235–50. doi: 10.1007/s00216-021-03860-0 (PMC8821073; doi:10.1007/s00216-021-03860-0)
Supplement: Supplementary file 7 — Supplementary file7 (PDF 605 kb) [file 216_2021_3860_MOESM7_ESM.pdf]

Electronic Supplementary Material_7; Data file S3

Sequential lipidomic, metabolomic and proteomic analyses of serum, liver and heart tissue specimens from peroxisomal biogenesis factor 11α knockout mice

Vannuruswamy Garikapati[1,2], Claudia Colasante[2], Eveline Baumgart-Vogt[2*], Bernhard Spengler[1*]

[1]Institute of Inorganic and Analytical Chemistry, Justus Liebig University Giessen, 35392 Giessen, Germany

[2]Institute for Anatomy and Cell Biology II, Division of Medical Cell Biology, Justus Liebig University Giessen, 35392 Giessen, Germany

Bioinformatics analysis using PANTHER (v14.1) classification system

Wild type control and peroxisomal biogenesis factor 11α knockout mouse liver proteome

Molecular functions

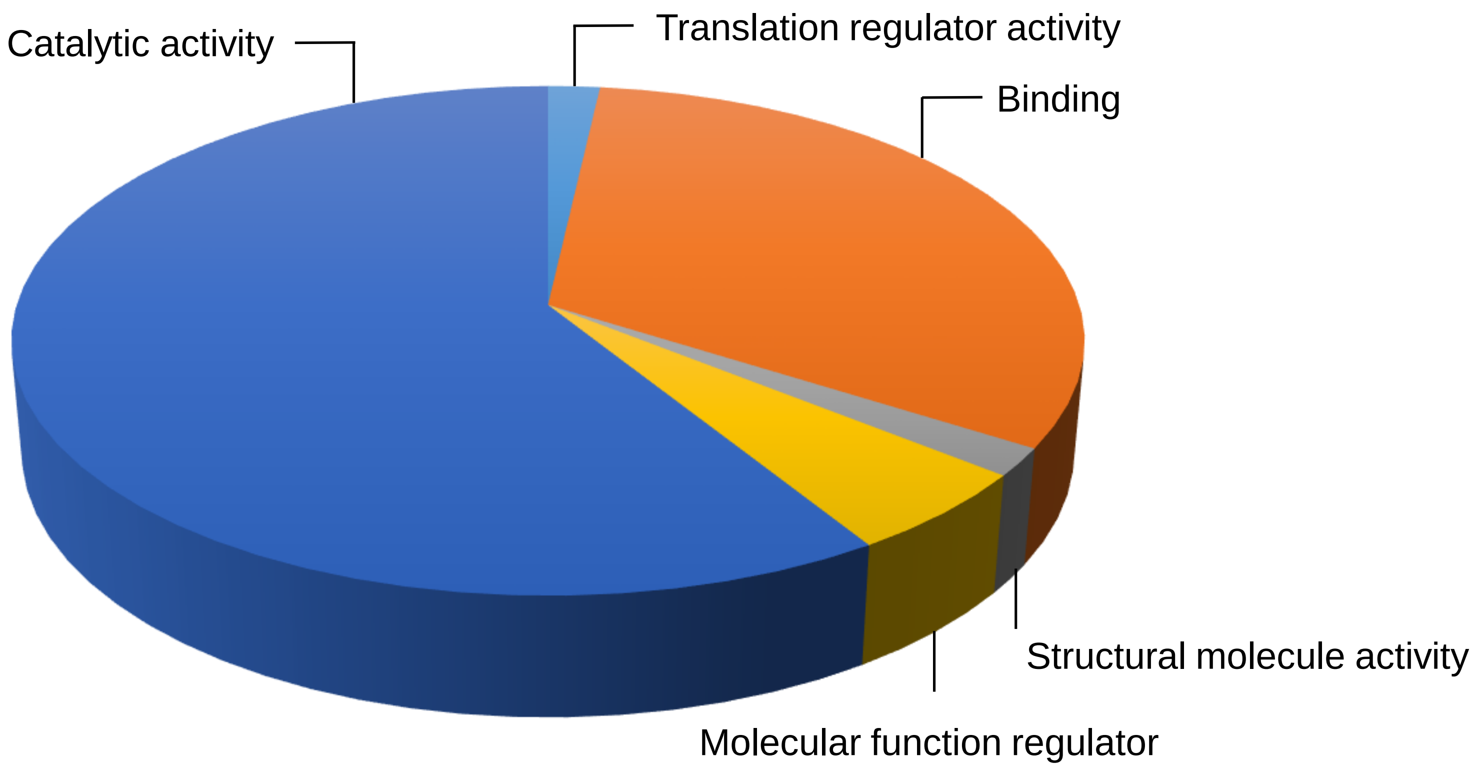

Bioinformatics analysis of the differentially abundant protein dataset (liver) depicting the molecular functions of the proteins

Cellular components

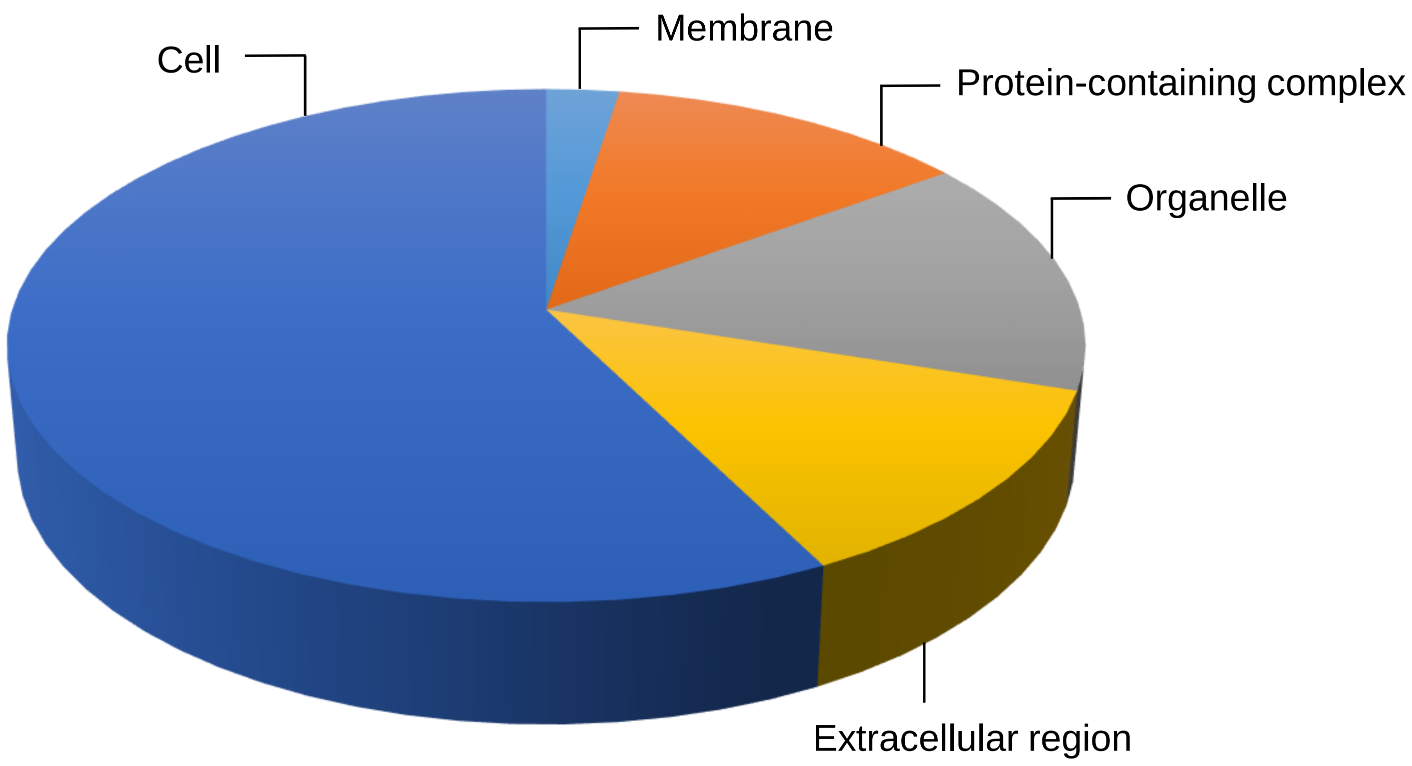

Bioinformatics analysis of the differentially abundant protein dataset (liver) depicting the cellular components of the proteins

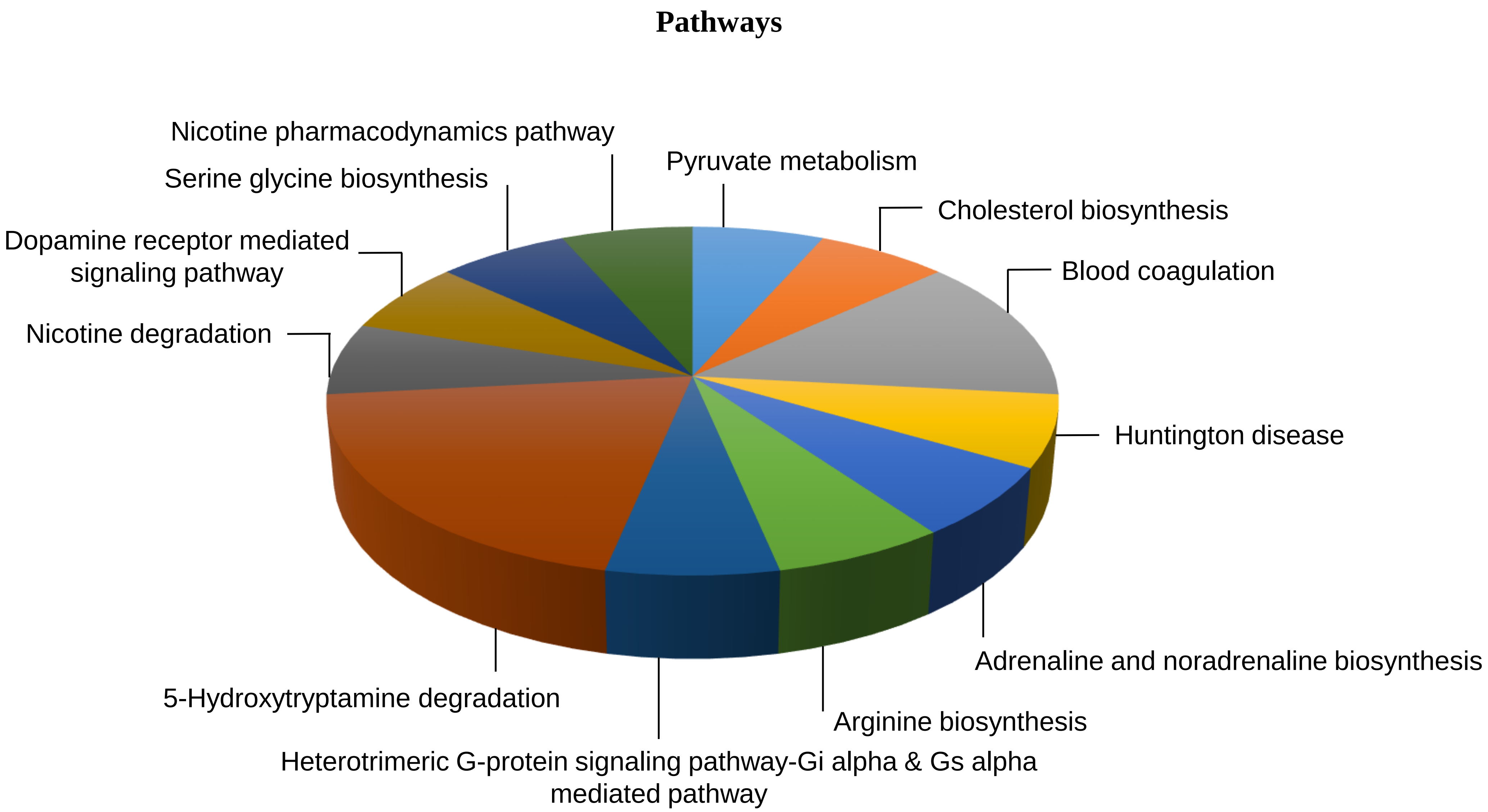

Bioinformatics analysis of the differentially abundant protein dataset (liver) depicting the pathways altered

Protein classes

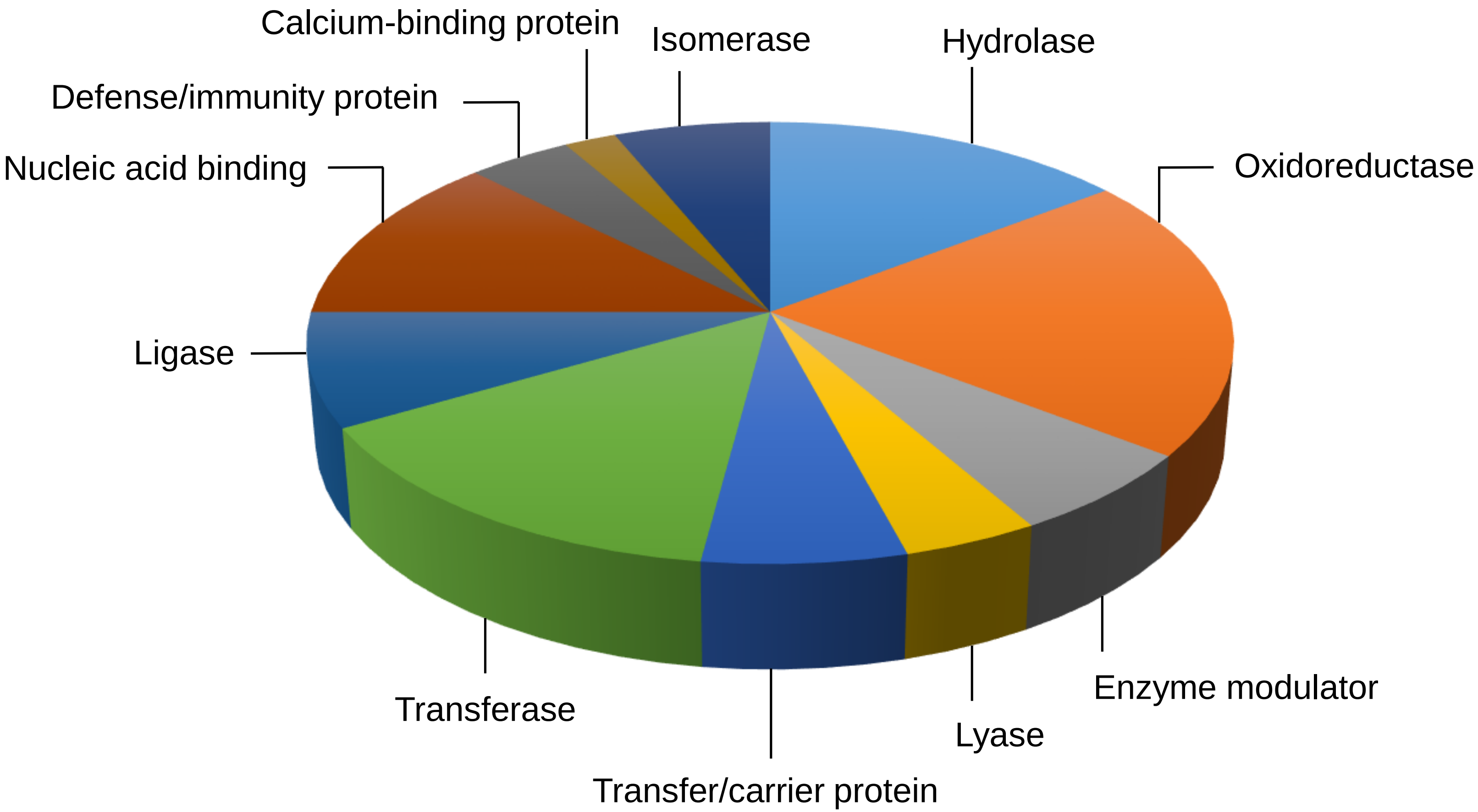

Bioinformatics analysis of the differentially abundant protein dataset (liver) depicting the protein classes

Bioinformatics analysis using PANTHER (v14.1) classification system

Wild type control and peroxisomal biogenesis factor 11α knockout mouse heart proteome

Molecular functions

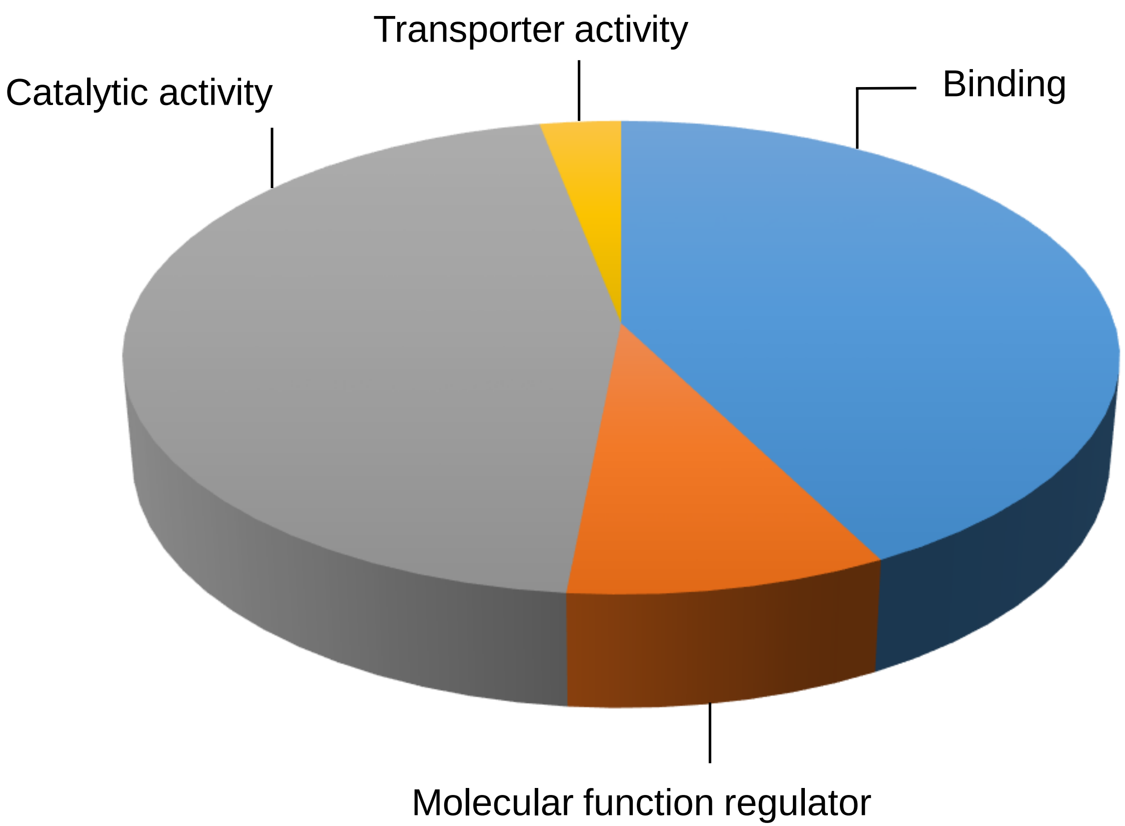

Bioinformatics analysis of the differentially abundant protein (heart) dataset depicting the molecular functions of the proteins

Cellular components

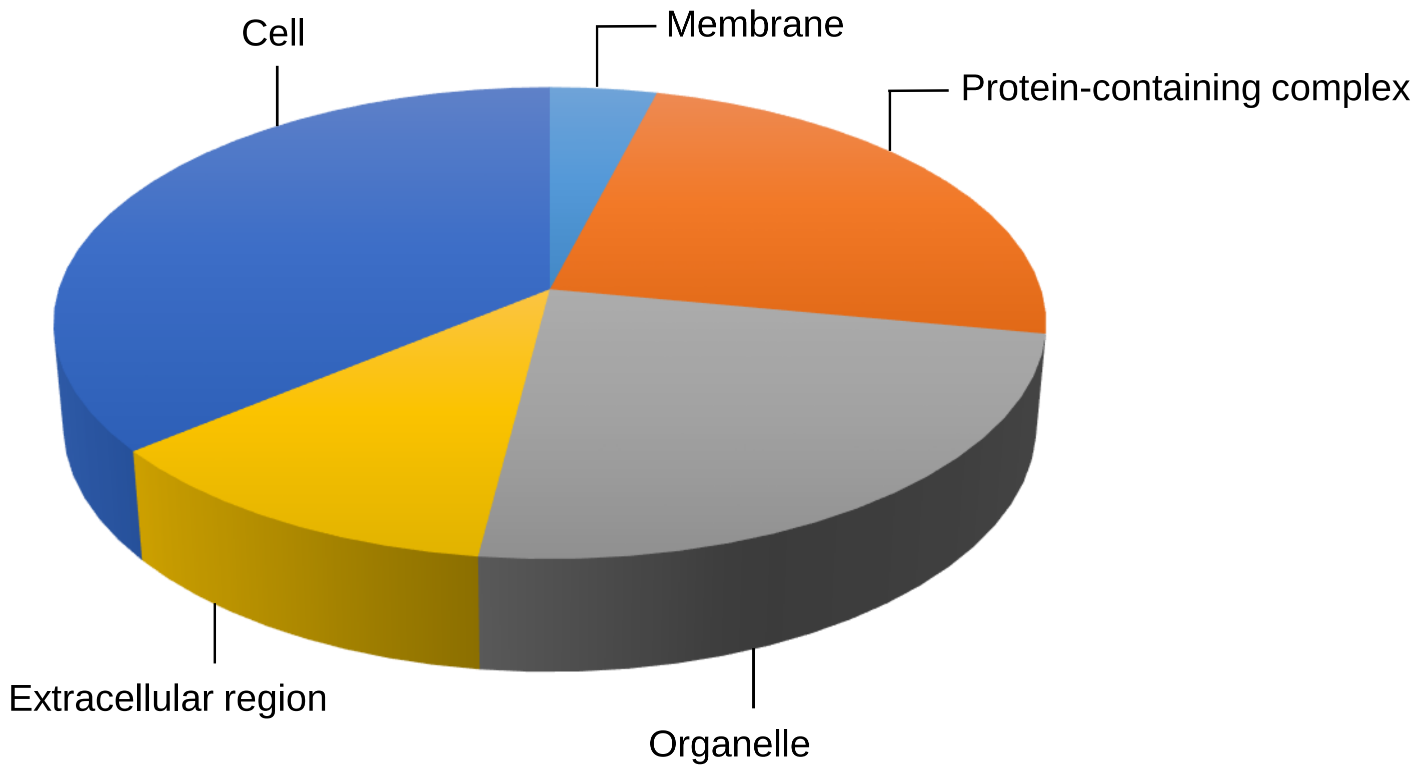

Bioinformatics analysis of the differentially abundant protein dataset (heart) depicting the cellular components of the proteins

Pathways

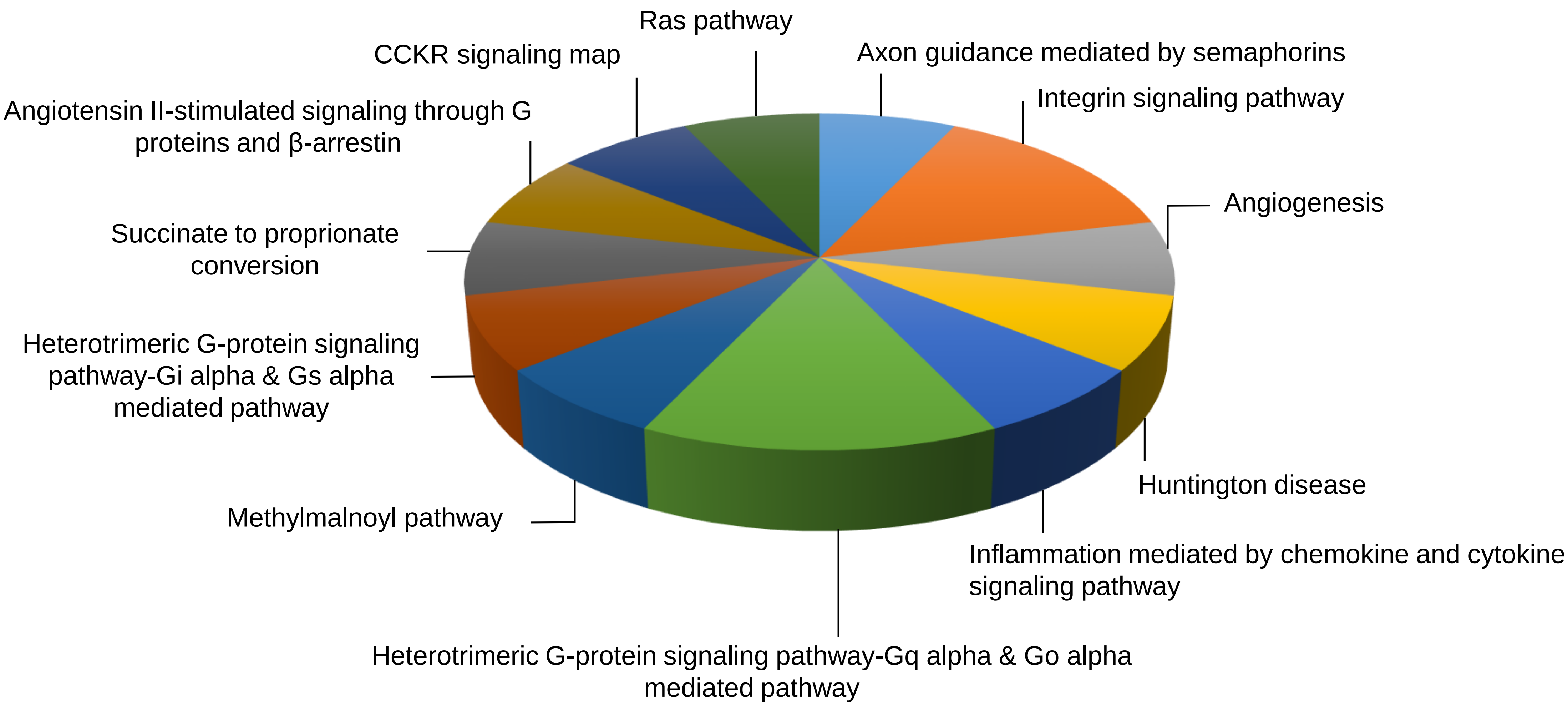

Bioinformatics analysis of the differentially abundant protein dataset (heart) depicting the pathways altered

Protein classes

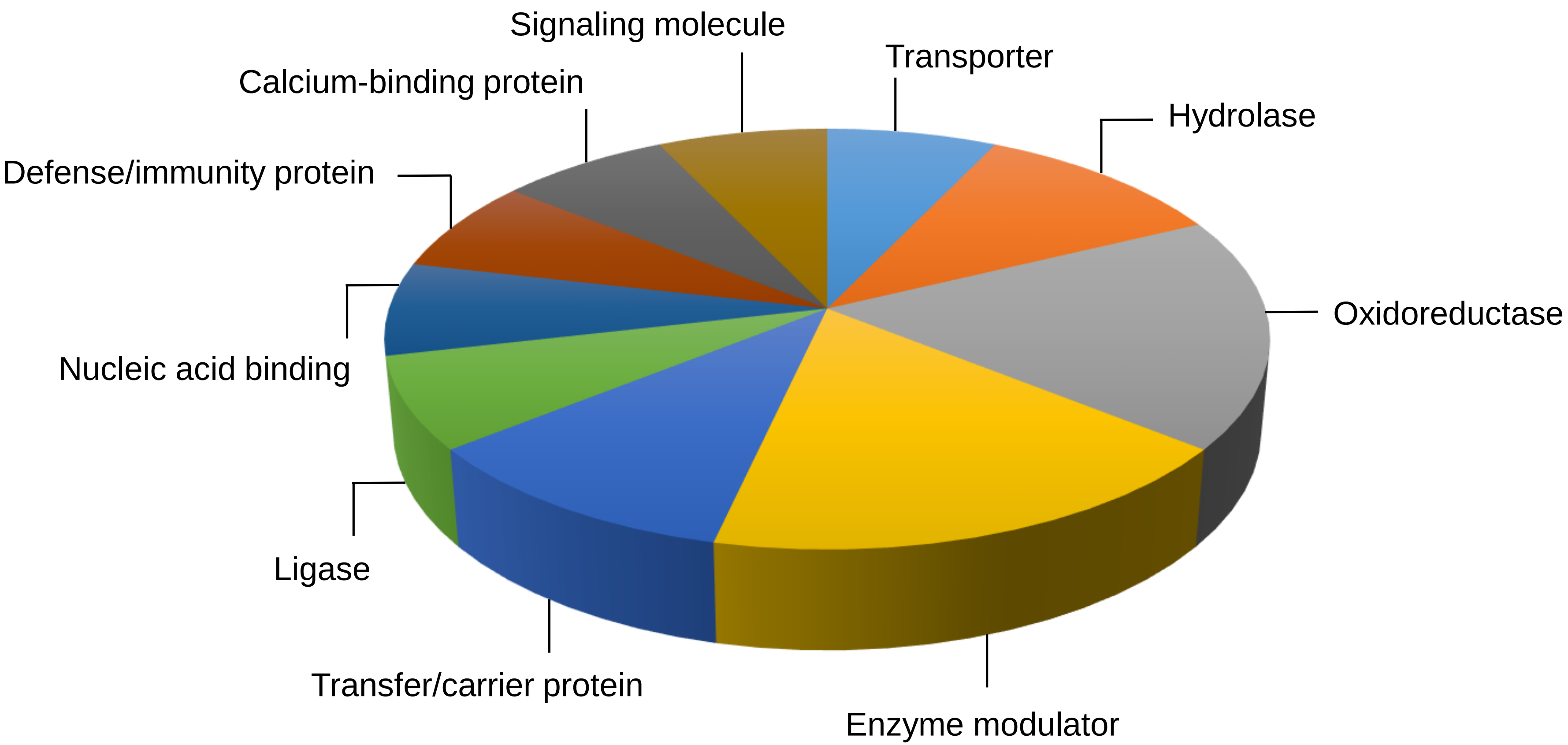

Bioinformatics analysis of the differentially abundant protein dataset (heart) depicting the protein classes

Bioinformatics analysis using Cytoscape (v3.7.2) and BiNGO (v3.0.3)

Wild type control and peroxisomal biogenesis factor 11α knockout mouse liver proteome

Molecular functions **Cellular components**

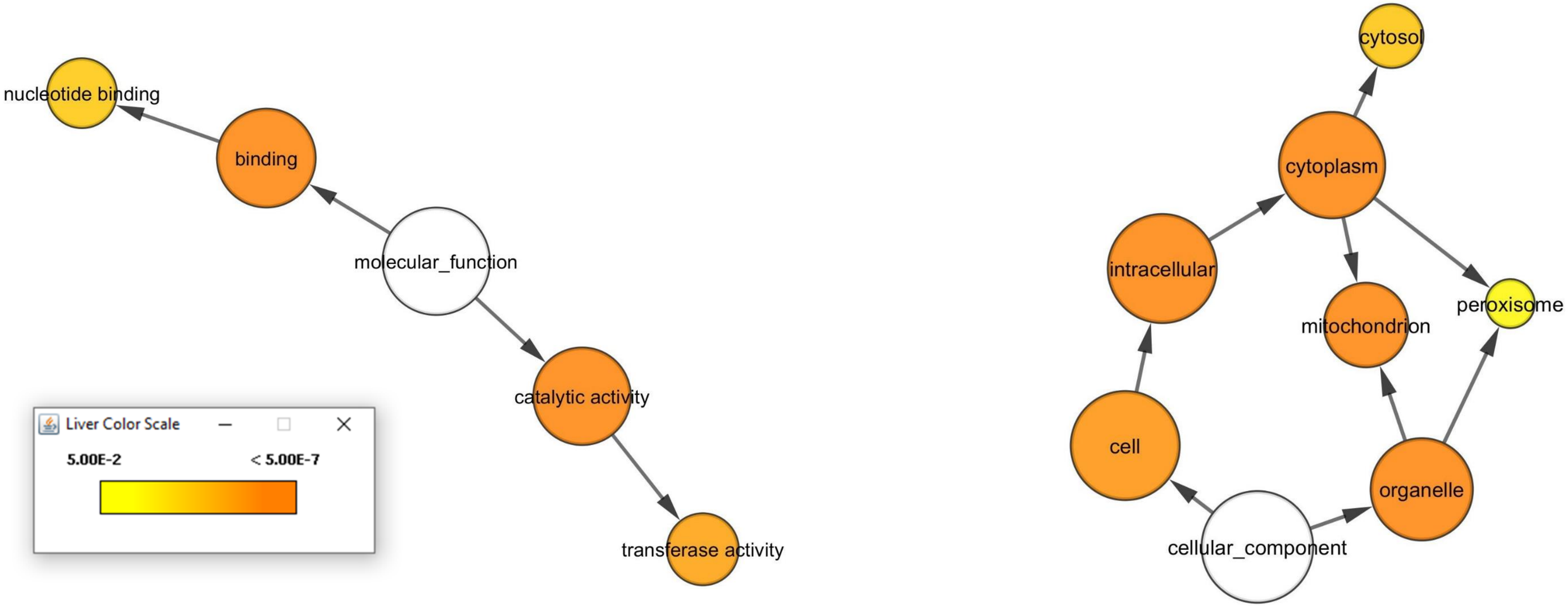

Bioinformatics analysis of the differentially abundant protein (liver) dataset depicting the molecular functions and cellular components of the proteins

Bioinformatics analysis using Cytoscape (v3.7.2) and BiNGO (v3.0.3)

Wild type control and peroxisomal biogenesis factor 11α knockout mouse heart proteome

Molecular functions **Cellular components**

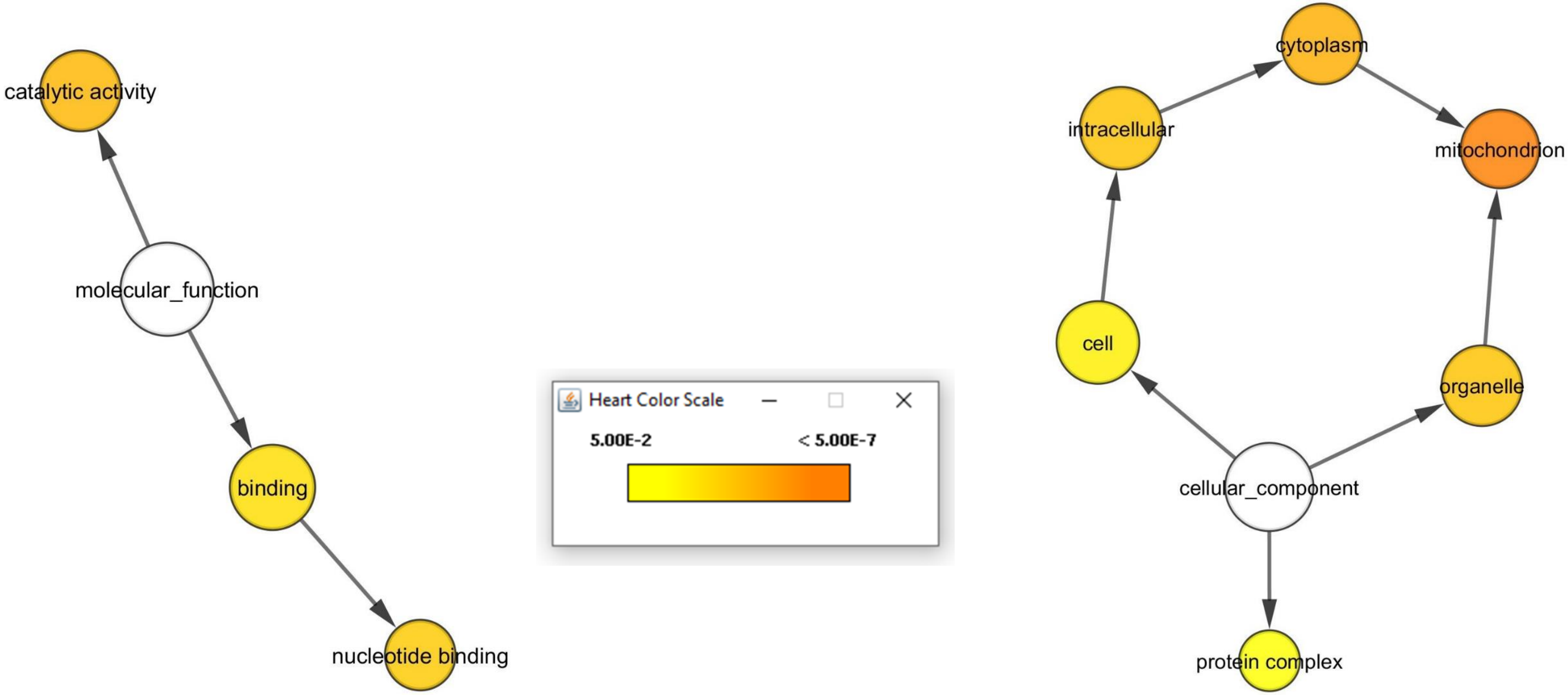

Bioinformatics analysis of the differentially abundant protein (heart) dataset depicting the molecular functions and cellular components of the proteins
